# Single-Tear Proteomics: A Feasible Approach to Precision Medicine

**DOI:** 10.3390/ijms221910750

**Published:** 2021-10-04

**Authors:** Erika Ponzini, Diletta Ami, Alessandro Duse, Carlo Santambrogio, Antonella De Palma, Dario Di Silvestre, Pierluigi Mauri, Fabio Pezzoli, Antonino Natalello, Silvia Tavazzi, Rita Grandori

**Affiliations:** 1 Materials Science Department, University of Milano-Bicocca, Via R. Cozzi 55, 20125 Milan, Italy; alessandro.duse@unimib.it (A.D.); fabio.pezzoli@unimib.it (F.P.); silvia.tavazzi@unimib.it (S.T.); 2 Department of Biotechnology and Biosciences, University of Milano-Bicocca, Piazza della Scienza 2, 20126 Milan, Italy; diletta.ami@unimib.it (D.A.); carlo.santambrogio@unimib.it (C.S.); antonino.natalello@unimib.it (A.N.); 3 Institute of Technologies in Biomedicine, National Research Council (ITB-CNR), Via Fratelli Cervi 93, 20090 Segrate, Italy; antonella.depalma@itb.cnr.it (A.D.P.); dario.disilvestre@itb.cnr.it (D.D.S.); pierluigi.mauri@itb.cnr.it (P.M.)

**Keywords:** lacrimal film, liquid biopsies, peripheral body fluids, personalized medicine, single-tear analysis, mass-spectrometry-based proteomics

## Abstract

Lacrimal fluid is an attractive source of noninvasive biomarkers, the main limitation being the small sample amounts typically collected. Advanced analytical methods to allow for proteomics profiling from a few microliters are needed to develop innovative biomarkers, with attractive perspectives of applications to precision medicine. This work describes an effective, analytical pipeline for single-tear analysis by ultrahigh-resolution, shotgun proteomics from 23 healthy human volunteers, leading to high-confidence identification of a total of 890 proteins. Highly reproducible quantification was achieved by either peak intensity, peak area, or spectral counting. Hierarchical clustering revealed a stratification of females vs. males that did not emerge from previous studies on pooled samples. Two subjects were monitored weekly over 3 weeks. The samples clustered by withdrawal time of day (morning vs. afternoon) but not by follow-up week, with elevated levels of components of the immune system in the morning samples. This study demonstrates feasibility of single-tear quantitative proteomics, envisaging contributions of this unconventional body fluid to individualized approaches in biomedicine.

## 1. Introduction

Liquid biopsies, such as blood, urine, and cerebrospinal fluid, are commonly employed to diagnose and monitor systemic diseases. Among these, tears have attracted growing interest, thanks to their relatively low complexity and easy accessibility. The tear film is formed by two distinct layers: an outer lipid layer and an inner aqueous layer containing proteins, metabolites, electrolytes, mucins, and transmembrane glycoproteins [1]. The composition of these layers reflects the pathophysiological state of the tissues underneath, as well as that of the whole body, which makes tears an attractive source of potential biomarkers for the evaluation of health and disease states [2].

Thanks to the high protein concentration (approximately ranging between 4 and 10 μg/μL in basal, open-eye tears) [3,4], tears can be easily analyzed by proteomics approaches, despite the low amount of sample that can be normally collected (around 6 μL from a single withdrawal) [5].

Protein identification and quantification in tear samples can be achieved by fast, accurate, and high-throughput approaches based on mass spectrometry (MS) [6]. Several authors have employed label-based techniques with isobaric tags [7,8,9]. Recently, label-free, quantitative proteomics approaches are gaining interest, thanks to the improved performance of the most advanced liquid chromatography (LC)-MS platforms [10]. Thus, MS-based proteomics of tear samples is a promising approach to biomarker discovery for human diseases, which can then be combined with orthogonal techniques, such as Western blotting, for validation.

Most of the studies reported in the literature approach the investigation of tear proteomes from pooled specimens [11,12,13,14,15]. The pioneering work by Li and coworkers describes proteomics analysis of a single tear, i.e., a single withdrawal from one eye, leading to the identification of 54 proteins [11]. So far, this study has represented the only example of single-tear proteomics. Analyses conducted on pooled samples have led to improved identification efficacy [12,13,14,15]. However, the pooling procedure loses the information on individual variability.

Improved single-tear analysis is required, in order to obtain patient-specific profiles of protein expression levels and their response to controlled changes in lifestyle, treatments, and time. Modern medicine has devoted increasing attention to patient stratification, based on better-defined omics profiles [16]. Such levels of molecular description can lead to a significant contribution of quantitative proteomics to precision and personalized medicine, which has been based, instead, mainly on genetic profiling [17].

Another common strategy employed to maximize the number of identifications is the addition of a fractionation step prior to LC-MS/MS analysis, such as offline strong cation exchange (SCX) [13,14] or electrophoresis [11,12,15]. These procedures, however, increase the complexity of the experimental protocol, making its application to high-throughput studies more challenging. Moreover, additional steps increase experimental error, sample losses, and analysis costs. In this specific case, greater advantages can be provided by ultrahigh-resolution MS analysis and high-performance LC separation, which were made possible by recent technological advances [18].

Vibrational spectroscopy has proved to be a complementary technique to MS-based approaches. In particular, Fourier transform infrared (FTIR) and Raman (RS) spectroscopies are commonly documented to be the primary approach for the analysis of chemicals, tissues, isolated cells, and liquid biopsies [19]. They rely on different principles, each of them having its peculiarities: FTIR measures the absolute frequencies at which a given sample absorbs radiation, whereas RS measures the relative frequencies at which it scatters photons. For this distinction, some vibrations are FTIR active, such as the ones related to heteronuclear polar bonds or to nonsymmetrical apolar bonds and some others are Raman active, e.g., differentiation between single, double, and triple carbon bonds.

MS-based proteomics, supported by vibrational spectroscopies, can now provide the accuracy and sensitivity necessary for single-tear profiling without upstream fractionation. With this aim, the present work describes a straightforward and high-performance approach to in-depth, single-tear quantitative proteomics.

## 2. Results

### 2.1. Protein Extraction

Protein extraction from tear samples was approached by methanol─chloroform precipitation. RS and FTIR techniques were employed for preliminary characterization of the different phases obtained from the extraction procedure: the unprocessed tear sample (tear), the aqueous layer (S1), the organic solvent layer (S2), and the protein pellet (P). These spectroscopic tools provided information on the content and structure of the different biomolecules in complex biological matrices.

**Figure 1:**
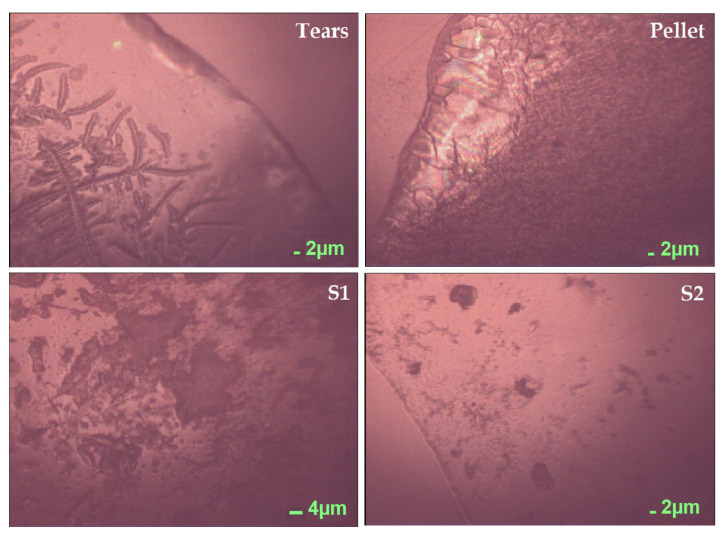
Pictures of the protein extraction phases acquired by the optical microscope integrated within the Raman instrument. Tears, unprocessed sample; S1, aqueous layer; S2, organic solvent layer; P, pellet.

The dehydration of a biological fluid in a controlled environment leads to the formation of a distinctive pattern called “ferning”. Commonly found in tears, its structure presents a clear and transparent circle surrounding the sample, denominated “coffee ring” [20], in which a major concentration of proteins can be found (Figure 1, Panel “Tears”). Internally, the dynamic of the solutes is based on salt─macromolecule interactions: crystalline nuclei and branches are mainly composed of sodium and potassium chloride, coated by macromolecular compounds, i.e., proteins and mucins that occasionally form globular structures [20]. At the nucleation stage of the crystallization process, the macromolecules compete with electrolytes on the growing crystal face of the nucleus and this process leads to the dendritic crystallization, according to the “fern” pattern [20]. Both the excess of electrolytes and the lack of macromolecular species prevent the formation of fern branches at the crystal faces of the cubic nucleus, until a sufficient coating of macromolecular material is accumulated at the apices. The extension of the main arm is then interrupted in favor of side branches that bifurcate as more organic material accumulates at the ends [20]. Ferning patterns are complex and have proven themselves useful in the differentiation of dynamics and composition of tears collected from different animals [21]. In Figure 1, the four dry patterns clearly demonstrate the different dynamics occurring, due to the variation of components in the sample. Tears display the characteristic features to be ascribed to the ferning pattern, which is clearly altered in the pellet, S1, and S2 due to the fractioning of the components. In the pellet, the coffee ring is fragmented, discontinuous, and incomplete, surrounding an amorphous pattern at the center of the sample. S1 and S2 are characterized by the dilution of molecules within the drop, leading to the heterogeneous formation of irregular dark structures, in the first, and spheroidal agglomerates, in the latter. 

**Figure 2:**
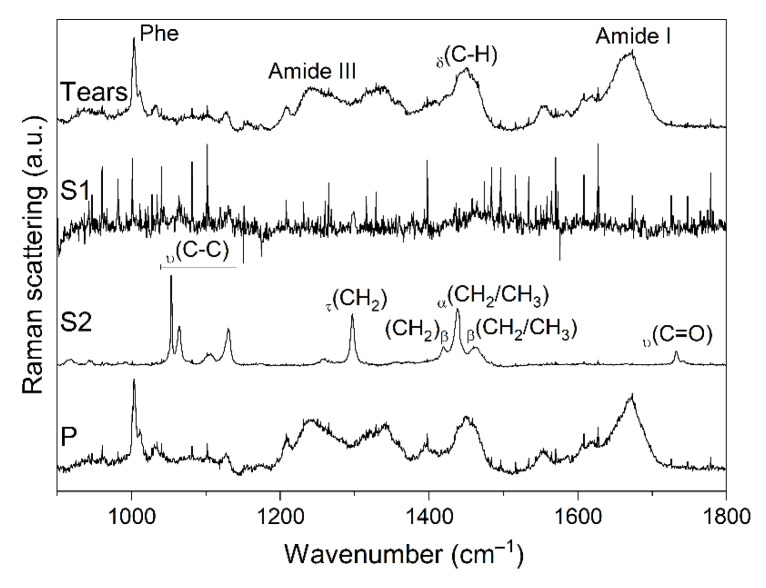
Raman (RS) spectra of protein extraction phases. Tears, unprocessed sample; S1, aqueous layer; S2, organic solvent layer; P, pellet. The assignment of selected peaks is shown, employing the annotation suggested by Czamara et al. (α, scissoring; β, bending; τ, twisting; υ, stretching) [24].

As shown in Figure 2, the Raman spectra of unprocessed tears and pellet display a similar fingerprint that can be ascribed to the main contribution of the protein component. The peaks at ~1003, 1240, 1450, and 1670 cm^−1^ may correspond, respectively, to vibrational modes of phenylalanine or symmetric C–N stretch of urea [22], amide III structures (associated to coupled C-N stretching and N-H bending), deformation of C-H bonds, and amide I structures (associated to C=O stretching) [23]. The spectrum of organic phase S2 is characterized by a high signal-to-noise ratio, identifying scissoring and twisting vibrations of the CH_2_ and CH_3_ groups and C–C stretching modes in the regions 1500–1400 cm^−1^, 1300–1250 cm^−1^, and 1200–1050 cm^−1^ [24]. In addition, a peak at ~1731 cm^−1^ derives from the stretching vibration of C=O groups [24]. Peak assessment proved more challenging for the aqueous phase S1, due to the poor signal-to-noise ratio related to the dilution of the molecules within the laser focus.

**Figure 3:**
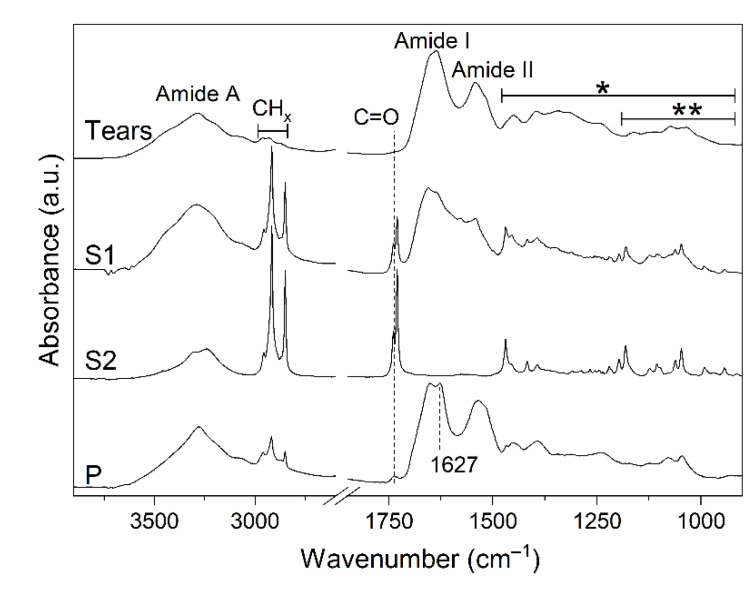
Fourier transform infrared (FTIR) spectra of protein extraction phases. Tears, unprocessed sample; S1, aqueous layer; S2 organic solvent layer; P, pellet. The wavenumber and the assignment of selected peaks are shown. * Overlapping absorption of lipids (hydrocarbon chains/head groups) and proteins; ** Overlapping absorption of carbohydrates and phosphates.

The results of the FTIR analysis are shown in Figure 3. The FTIR spectra of unprocessed tears and of the aqueous phase S1 are very complex, due to the overlapping absorption of different molecules, including proteins (Amide A, I, and II bands), lipids, and carbohydrates. The spectrum of the organic phase S2 displays, instead, features ascribable only to lipid absorption. Finally, the spectrum of the pellet indicates predominance of protein absorption. In particular, the peak at ~1627 cm^−1^ can be assigned to β-sheet structures in protein aggregates [25].

Both analyses highlight the complexity of the aqueous phase S1, which contains lipids, proteins, carbohydrates, nucleic acids, and metabolites (Figure 2 and Figure 3). On the other hand, the organic phase S2 is much simpler and contains mainly lipids. No signal ascribable to other classes of molecules can be identified. The spectra of the pellet, instead, can be interpreted by predominance of protein species, indicating good recovery and enrichment of the protein content in this fraction. The intense peak at 1627 cm^−1^ in the FTIR spectrum suggests that proteins aggregate during this process, losing their native structure. In this case, this is not an issue since the whole proteome will be digested for protein identification and quantification by shotgun proteomics.

### 2.2. LC-MS/MS Analysis

Tear fluid was collected from each volunteer by microcapillary tubes (MCT), and the protein content was isolated by methanol─chloroform precipitation. The purified proteins were reduced, alkylated, and digested by trypsin, before LC-MS/MS analysis.

With this approach, it was possible to identify 932 proteins by at least one unique peptide, at a false discovery rate (FDR) of 1.0%. A total of 42 species were keratins or keratin-associated proteins (Supplementary Material S2), which can be ascribed to possible contamination during tear collection and sample processing. These hits were filtered out, resulting in a list of 890 proteins (Supplementary Material S3). The average number of identifications per run was 579 ± 69, with a good run-to-run reproducibility (mean R^2^: 0.81 ± 0.17) on the same sample (*data not shown*).

To date, only five studies focused on proteomic investigation of healthy human tears, aiming to optimize the analytical procedure and maximize the proteome coverage [18]. The number of proteins identified in tears has remarkably increased since the first publication in 2005 [11], achieving the highest proteome coverage (1543 proteins) in a paper published in 2012 [13]. In three out of five studies, samples from different subjects were pooled together, in order to increase the sample volume to handle and the proteome coverage [13,14,15]. Nevertheless, sample identity and potentially meaningful individual variability are unavoidably lost by pooling. An alternative approach consists in the pooling of sequential tear collections from a single subject, as performed by de Souza [12], who achieved the identification of 491 proteins. Nonetheless, information on intra and interday variability of the tear proteome is lost, even in this case.

Thanks to ultrahigh-resolution MS and high-performance LC separation, the present work increases the number of identified proteins in a single-tear sample from about 50 proteins [11] to almost a thousand.

### 2.3. Analytical Power Evaluation

**Figure 4:**
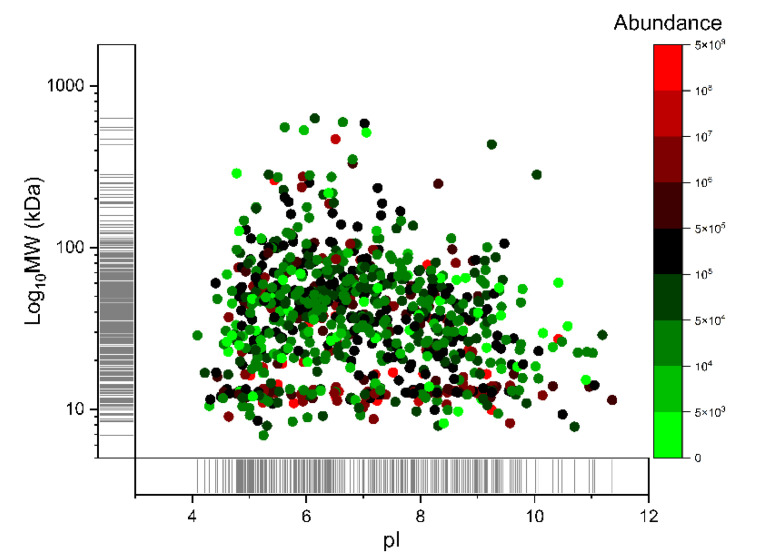
Virtual 2D map (theoretical MW vs. pI) of the identified proteins. The mean abundance of each protein is represented by the color scale reported on the right side, calculated as the average from its 3 most intense peptides, over 70 runs.

Label-free shotgun proteomics was performed on the pellet fractions of the methanol─chloroform precipitation procedure. The UniProtKB molecular weight, the calculated pI and the mean abundance of each protein were employed to generate a virtual two-dimensional (2D) map of the identified proteins (Figure 4) [26]. Differently from traditional 2D gels of tear samples, where proteins seem to cluster in a few regions, the proteins identified by MS are quite homogeneously distributed in the range 4.5–10 of pI and 50–500 kDa of MW [27]. This difference can be ascribed to the superior sensitivity and dynamic range of the shotgun approach. The highest-MW proteins are mainly represented by mucins (mucin 16, 5B, 5AC, and 7), a protein family (MUC) that have been sub-classified into secreted and transmembrane forms. The secreted mucins (for example, MUC5AC and MUC5B) form a mucous gel, which acts as a physical barrier protecting the epithelial cells of the ocular surface [28]. Mucins are also expected to play a role from a tribological viewpoint. They were reported to be one of the main components contributing to the viscosity of tears [29]. Furthermore, friction due to mechanical forces has been reported to follow from insufficient mucins or altered composition of the resident mucins at the ocular surface [30].

The highest-pI proteins are represented by histones (pI ~11), with H4 having the highest pI (11.36), followed by ribosomal proteins (pI ~10.5) that have already been found in tear fluid [13]. The presence of histones in the tear film, as well as that of proteins such as neutrophil elastase and nucleases, can be ascribed to the corneal epithelial cell shedding process, or desquamation, which is regulated by apoptotic mechanisms [31]. However, histones can also translocate from the nucleus to the extracellular space, acting as damage-associated molecular pattern molecules [32]. In addition, even though care was taken to avoid it, cell contamination could have taken place during sample collection and preparation.

The abundance of each protein has been calculated as the average intensity from its three most intense peptides. Most of the proteins display an intensity between 1 × 10^4^ and 1 × 10^7^ (87.5%), whereas only few proteins have higher intensities (3.6%), with lysozyme having the highest average intensity (4.05 × 10^9^).

**Figure 5:**
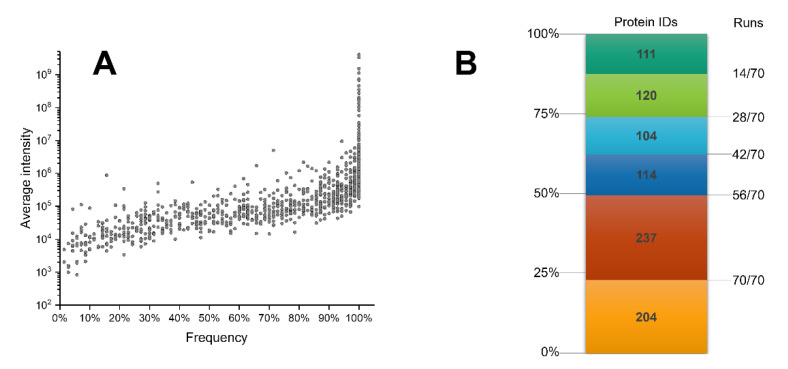
Identification sensitivity and reproducibility. (**A**) Correlation between the frequency of identification of each protein over the 70 runs and its average intensity. (**B**) Stacked, colored boxes represent the identification frequencies over the 70 runs.

The low abundance of most of the proteins could represent a limit in terms of proteome coverage. The proteins identified with lowest frequency (1 out of the 70 analyses performed) have, indeed, a very low abundance (below 1 × 10^4^). However, as shown in Figure 5, Panel A, there is no pronounced correlation between protein abundance and identification frequency. Here, a common core of 204 proteins is identified in all runs (~23% of the overall identified proteins), despite the difference in average intensity, ranging from 10E + 4 to 10E + 9. A previous study reported an overlap of 45.5% in the sets of proteins identified in three different analyses of pools of two or three individuals [15].

The 10 most abundant proteins are the same for each run, with lysozyme being the most abundant protein of the human tear proteome. Another important protein found in this list is lactotransferrin, one of the major proteins of tears, involved in the anti-inflammatory and antimicrobial processes [33], including antiviral activity. Of note, lactoferrin administration through eye drops has proved effective on SARS-CoV-2-related conjunctivitis [34]. The predominant tear proteins are directly secreted by lacrimal glands (lactotransferrin, lipocalin-1) or by lysosomes (lysozyme C). Lysozyme C, lactotransferrin, and lipocalin-1 are the most abundant and frequent proteins among the 894 proteins identified here and are secreted by the acini of the main gland. Therefore, they act as indicators of lacrimal gland function. Lower levels correlate with inflammation, lower antioxidant function, and higher predisposition towards microbial infections [35]. However, some serum proteins, like albumin, transferrin, IgG, and IgM are also found in the tear fluid, probably as a result of passive transport from the blood [3]. It is also known that cells infiltrating the conjunctiva (T and B cells, among others) secrete IgGs and cytokines under various conditions [36].

**Figure 6:**
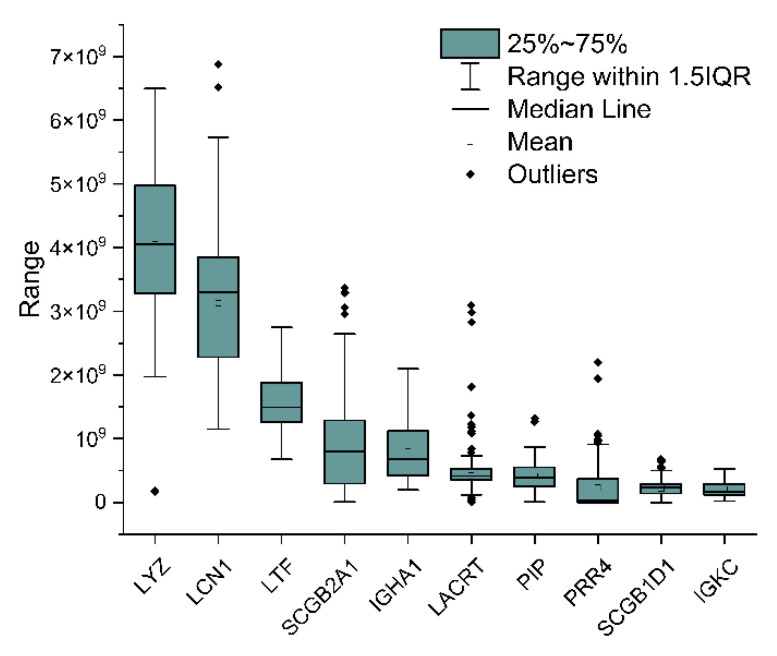
Box plot of the intensity of the 10 most abundant proteins over 70 runs. Each box represents the interquartile range between the 25th and the 75th percentile, whereas the whiskers represent the range between the 5th and the 95th percentile. The median and mean values are also reported, as a line and an empty square, respectively. Outliers are represented by black diamonds. Proteins are indicated by their gene name: LYZ, lysozyme C; LCN1, lipocalin-1; LTF, lactotransferrin; SCGB2A1, mammaglobin-B; IGHA1, immunoglobulin heavy constant alpha 1; LACRT, extracellular glycoprotein lacritin; PIP, prolactin-inducible protein; PRR2, proline-rich protein 4; SCB1D1, secretoglobin family 1D member 1; IGKC, immunoglobulin kappa constant.

The intensities of the 10 most abundant proteins are reported in Figure 6. Lysozyme and lipocalin-1 display a particularly broad range of intensities across the samples, suggesting a large intersubject variability. This variability has been reported previously in a study employing selected reaction monitoring (SRM)-MS to quantify these proteins in single-tear samples [37].

**Figure 7:**
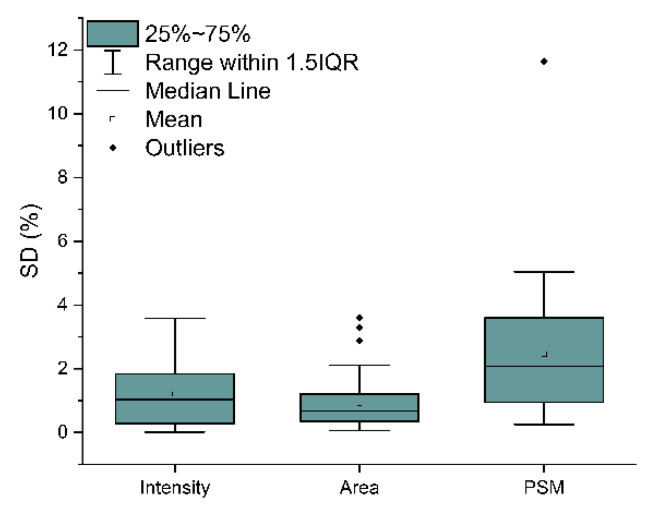
Box plot of the standard deviation obtained in the label-free quantification (LFQ) by normalized peak intensity, peak area, and peptide spectrum match (PSM).

In order to evaluate the precision correlated to the label-free quantification (LFQ) parameters, the variability obtained by intensity was compared to those calculated by peak area and peptide spectrum match (PSM), applied to the same collected tear samples. Specifically, using non-normalized values, the variability resulted in values between 4% and 5% for intensity and peak area, and 2.3% for PSM (*data not shown*). Instead, values normalized over the total signal revealed a variability between 1% and 2% for intensity, peak area, and PSM (Figure 7). These findings evidence that the three LFQ normalized parameters present a similar precision.

### 2.4. Gene ontology Analysis

**Figure 8:**
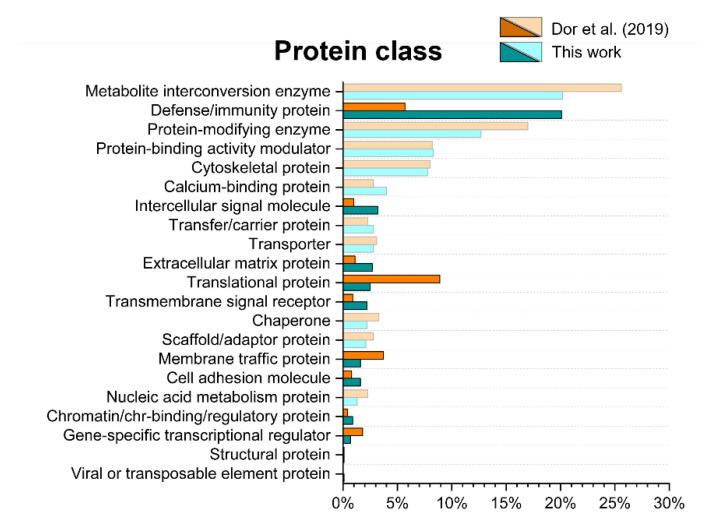
Bar charts of the protein classes obtained with the 890 proteins identified in this work, using the Protein Analysis Through Evolutionary Relationships (PANTHER) program. The results are compared with the protein list published by Dor and coworkers [15]. Protein classes displaying a log_2_(fold change) ≥|1| are highlighted by stronger saturated colors.

Using the functional annotation clustering tool from Protein Analysis Through Evolutionary Relationships (PANTHER), the 890 proteins were classified according to molecular functions, cellular components, and biological processes (Supplementary Material S4), as well as protein classes (Figure 8). The same analysis was performed on a previously published list, as reported by Dor and coworkers [15].

The categories emerging from all the Gene Ontology (GO) analyses are the same for both studies, and most of them show a similar protein distribution. However, there are some protein classes displaying a log_2_(fold change) ≥|1|. Compared to the protein list published by Dor and coworkers, the present study reports a higher amount of defense/immunity proteins, intercellular signal molecules, extracellular matrix proteins, transmembrane signal receptors, cell adhesion molecules and chromatin/chromatin-binding/regulatory proteins. On the other hand, the previously published list looks enriched in translational proteins, membrane traffic proteins and gene-specific transcriptional regulators. These results indicate that translational and transcriptional proteins are less represented in this work, whereas extracellular proteins are more represented. These differences might be due to the different collection methods employed [5]. The MCT method has been reported to produce a lower cellular contamination than Schirmer tear strips (STS) [38].

Dor and coworkers also performed a KEGG analysis, where the complement and coagulation cascades (38 proteins, *p*-value 1.7 × 10^−20^) and the glycolysis/gluconeogenesis pathway (31 proteins, *p*-value 4.6E−14) are the two most represented pathways. In the present study, the two most represented biological pathways by KEGG analysis are the lysosome pathway (41 proteins, *p*-value 1.4 × 10^−20^, Supplementary Material S5) and the complement/coagulation cascade (23 proteins, *p*-value 1.9 × 10^−11^, Supplementary Material S6). Lysosomal enzymes have been found in the lacrimal gland [39]. Lysosome activity regulates autophagy, which is in turn associated with various eye diseases, with aging being one of the most important factors [40]. The complement system is a key component of innate immunity and is activated not only under general inflammatory conditions such as infections, collagen diseases, nephritis, and liver diseases, but also in ocular diseases. The complement system has been reported to be critical in maintaining retinal integrity during aging [41,42].

### 2.5. Sample Stratification

**Figure 9:**
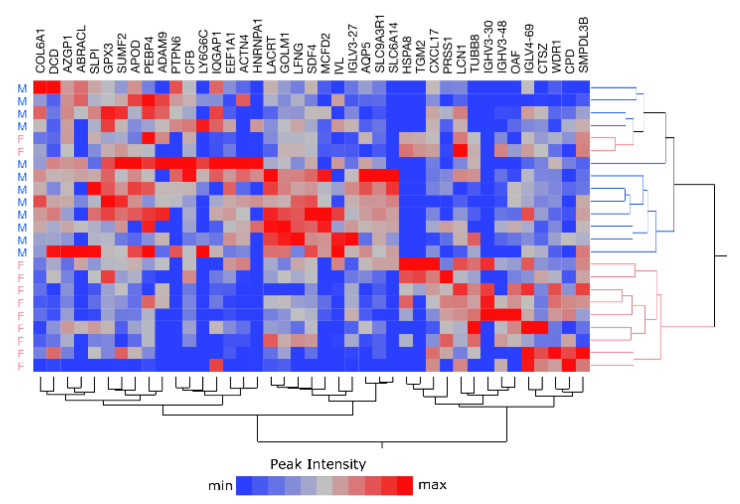
Double-hierarchical clustering analysis of 41 tear proteins selected by linear discriminant analysis (LDA) and 23 samples (pink: F, female; blue: M, male). LFQ by peak intensity is represented by a color code from blue to red, according to increasing abundances.

The analyses described so far were conducted over 23 samples, by merging all the lists of identified proteins and by averaging the relative abundances. However, the analysis of single-tear samples has the great advantage of preserving sample identity, offering the possibility to group samples according to different variables. All subjects were healthy, young, and displayed similar body mass indexes, while differing in sex: 12 males and 11 females out of 23 volunteers. All the withdrawals occurred in 2019, before the outbreak of COVID-19 pandemic in Italy (February 2020). Tear protein profiles were used to evaluate the potential subgrouping of subjects. Specifically, the average protein lists from the 23 subjects were used to perform a cluster analysis, based on the proteins identified in at least 30% of the runs. The resulting heatmap evidenced a good stratification of males vs. females (Figure 9) and the extraction of related descriptors (*p*-value ≤0.05). Of note, 35 out of 40 extracted descriptors (Supplementary Material S7) were confirmed by differential analysis based on the MaProMA platform [43].

### 2.6. Comparison of Morning vs. Afternoon Sample Collection

**Figure 10:**
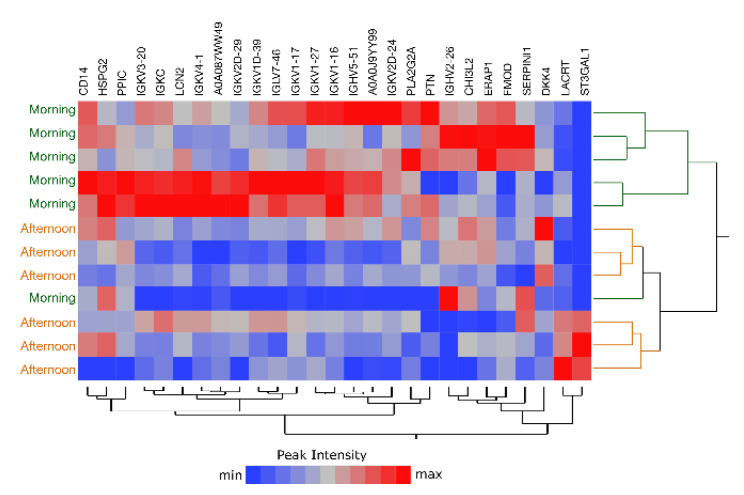
Double-hierarchical clustering analysis of 27 tear proteins selected by LDA and 12 samples (green, morning; orange, afternoon). LFQ by peak intensity is represented by a color code from blue to red, according to increasing abundances. Samples were collected weekly from two subjects over three weeks.

Single-tear analysis represents an appealing approach also for longitudinal studies, which involve monitoring given individual variables over short or long periods of time. In a proof-of-concept study, we asked two of the 23 subjects to volunteer for tear collection over 3 weeks, in the absence of treatments or habit changes. Collection was performed once a week, in the morning (10 AM) and in the afternoon (4 PM). The obtained protein lists results segregated according to the time of collection (morning vs. afternoon) (Figure 10). The 27 descriptors extracted by linear discriminant analysis (LDA) are listed in Supplementary Material S8. Twenty-five of them were confirmed by differential analysis based on the MaProMA platform.

## 3. Discussion

High-performance analytical procedures allow for reliable quantitative protein profiling from “single-tear” (5 µL) lacrimal fluid collected by the microcapillary method, without fractionation steps upstream from the LC-MS protocol. This work described a pipeline for data collection and analysis that can be of relevance for a wide array of biomedical applications. A systematic comparison of the alternative quantitation methods usually employed in the literature revealed a good equivalence of normalized peak intensity, peak area, and PSM over 70 runs. LDA and hierarchical clustering analyses revealed 41 descriptors for male vs. female stratification and 27 descriptors for morning vs. afternoon stratification. The effect of gender underscores the potential of tear as an informative biofluid and the importance of single-tear analysis in preserving sample identity. The effect of withdrawal time hints at the need for highly controlled sample-collection conditions, with variable protein profiles in afternoon vs. morning samples. Further studies will be needed to investigate the turnover of tear proteins and the role of sleep on tear proteome homeostasis. It would also be of interest to strengthen tear protein characterization and identification of group descriptors by multicentric studies. This work also suggests that proteomic and spectroscopic profiling could provide complementary information for biochemical and biophysical characterization of lacrimal fluid, possibly leading to the discovery of composite biomarkers.

Detailed stratification of individual patients by proteomics analysis is crucial to translate personalized medicine into practice. Tissue biopsies are informative, but their collection is not always simple or possible. For instance, practical and ethical issues restrict the collection of brain biopsies to post-mortem sampling. Localized biofluids, such as tears, contain contributions from different organs and tissues, making them interesting for both organ-specific and systemic diseases. In this frame, tear fluid has elicited a growing interest, thanks to its continuous accessibility, minimal storage requirements, high protein concentration, and responsiveness to both ocular and systemic conditions, particularly those linked to neurodegeneration (i.e., multiple sclerosis, Alzheimer’s disease, and Parkinson’s disease).

## 4. Materials and Methods

### 4.1. Volunteers’ Recruitment

23 volunteers (12 males and 11 females; average age of 23.7 ± 2.6 years; Supplementary Material S1) were recruited for this study. Inclusion criteria were: volunteers aged ≥18 and ≤35, any gender and ethnicity, able to express their consent. Exclusion criteria were: any systemic disease, ocular pathology, or cancer type. Written informed consent was collected from all volunteers, in accordance with the Declaration of Helsinki. The use of tears as biological material and the informed consent form were approved by the local ethics committee for research on human beings (approbation N° 0055071/19, 11 July 2019).

### 4.2. Sample Collection

Tears were collected using MCT (Hirschmann ringcaps, Hirschmann Laborgeräte GmbH & Co. KG, Eberstadt, Germany). For each volunteer, a single tear sample was taken from one eye. In order to collect only the basal fluid, no external stimulation was applied and any discomfort, such as prolonged time of collection, harsh light, etc., was avoided. Particular care was taken to avoid any damage to the conjunctiva and any eye irritation. At least 5 µL of nonstimulated tear (NST) was collected and each sample was immediately stored at −80 °C until analysis.

For two of these volunteers (both females, aged 19 and 21 years), the procedure was performed in the morning (9–10 AM) and in the afternoon (5–6 PM) on the same day, once a week for three consecutive weeks.

### 4.3. Protein Extraction

Proteins were purified from 5 µL of tear sample using the methanol─chloroform precipitation protocol, adapted from Wessel and Flügge [44]. Methanol:chloroform:H_2_O 4:1:3 was added to the sample, followed by centrifugation at 14,000× *g*. The aqueous layer on top (S1) was removed, leaving intact the protein layer at the interface, and an equivalent volume of methanol was added to the remaining solution, followed by centrifugation at 20,000× *g*. The organic solvent layer (S2) was removed, and the protein pellet (P) was lyophilized.

**Figure 11:**
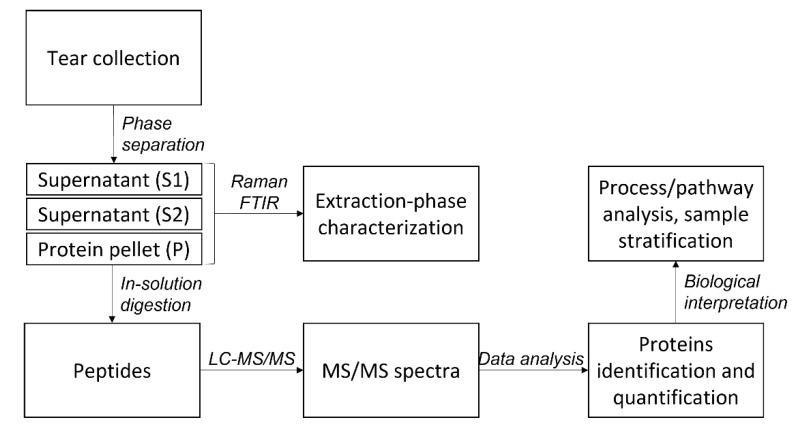
Flowchart illustrating the main steps of the analytical pipeline.

Vibrational spectroscopies were employed to compare the unprocessed tear sample with the different phases of the protein extraction. For one sample, the S1, S2, and P were lyophilized, resuspended in H_2_O:ethanol 1:1 to be analyzed by RS and FTIR, after deposition and dehydration. The resulting spectra were compared to the ones from an unprocessed tear of the same volunteer (Figure 11).

### 4.4. RS Analysis

RS investigation was carried out using a Horiba T64000 system in a single monochromator configuration. The backscattering geometry utilized a confocal microscope offering a lateral spatial resolution inferior to 1 µm. To minimize dark count rates, RS spectra were collected using a Si-based charge-coupled device, cooled by liquid nitrogen to the operating temperature of −125 degrees Celsius (148 K). The resulting spectral pitch was 2 cm^−1^. The objective providing the best signal-to-noise ratio was the Olympus M Plan Achromat-MPLN 100×/0.9. The excitation laser was a solid-state Nd-YAG, operated at a wavelength of 532 nm. A notch filter was integrated into the system to eliminate the Rayleigh component of the scattered light. The system was calibrated on a crystalline Si sample before each measurement session, providing a Raman shift value for the first-order LO-vibrational mode of 520.5 cm^−1^.

Time and range of acquisition were established after an on-tear calibration work, giving the best results in the 900–1800 cm^−1^ spectral window when 60 accumulations of 5 s were utilized at each monochromator position.

Each sample was deposited by drop-coating onto barium fluoride substrates, ensuring a straightforward preparation procedure [22]. Considering the heterogeneity within each sample, it was decided to measure recurring structures, if available, in all the examined specimens.

### 4.5. FTIR Analysis

FTIR spectra of unprocessed tears, as well as S1, S2 and pellet phases were collected in attenuated total reflection (ATR) by a Varian 670-IR spectrometer equipped with a nitrogen-cooled mercury cadmium telluride detector (Varian Australia Pty Ltd., Mulgrave, Australia). For each sample, 2 µL were deposited onto the diamond crystal of the single reflection ATR device (Specac Quest) and dried at room temperature. ATR-FTIR spectra were then collected under the following conditions: 2 cm^−1^ spectral resolution, scan speed of 25 kHz, 512 scan coadditions, and triangular apodization [25].

### 4.6. Protein Reduction, Alkylation, and Digestion

The lyophilized proteins were resuspended in ammonium bicarbonate (Merck, Darmstadt, Germany) 50 mM, at pH 8. Dithiothreitol (DTT, Merck, Darmstadt, Germany) was added to each sample to a final concentration of 10 mM. After 1 h incubation at 37 °C, 30 mM iodoacetamide (IAA, Merck, Darmstadt, Germany) was added and the samples were incubated 30 min in the dark. In-solution trypsin digestion (1:35 ratio based on the average total protein content of human tears [18], Trypsin Gold, Mass Spectrometry Grade, Promega Italia Srl, Milan, Italy) was performed upon addition of a final 50 mM DTT by an 18 h incubation at 37 °C. Digestion was stopped by formic acid (FA) to a final concentration of 1% v/v (Merck, Darmstadt, Germany).

### 4.7. LC-MS/MS Analysis

The samples were desalted by C18 ZipTips (Fisher Scientific, Waltham, MA, USA), lyophilized and resuspended in 0.1% FA (solvent A, Fisher Scientific, Waltham, MA, USA). A total of 1 µg of peptides was injected for each run and the analysis was performed twice, gaining technical duplicates. LC-MS/MS analyses were performed on an Orbitrap Fusion mass spectrometer (ThermoFisher, San Jose, CA, USA) coupled to an EASY-nLC 1000 nanoflow, high-pressure liquid chromatography (ThermoFisher, San Jose, CA, USA). Peptides were separated on a commercial analytical nanocolumn (EASY-Spray column ES803, 50 cm × 75 µm internal diameter, PepMap RSLC C18, 2 µm, ThermoFisher, San Jose, CA, USA) mounted on an EASY-Spray ion source (ThermoFisher, San Jose, CA, USA). The analytical separation had a duration of 155 min with a flow rate of 250 nL/min, using acetonitrile (ACN):H_2_O:FA 80:19.9:0.1 (solvent B, Fisher Scientific, Waltham, MA, USA) and solvent A. The LC scheme was as follows: 5% solvent B for 5 min; 5%–30% solvent B in 80 min; 30%–70% solvent B in 50 min; 70%–95% solvent B in 10 min; 95% solvent B for 10 min. The spray capillary voltage was set at 2.0 kV and the ion-transfer capillary temperature was held at 275 °C. MS scans were performed using the Orbitrap (OT) analyzer, with a resolution of 120,000 FWHM in positive ion mode. Precursor ions were selected in data-dependent mode by higher collisional dissociation (HCD) and the collision energy was set to 30%. Detection was performed by ion trap in rapid mode. Dynamic exclusion was set at 45 s, with a repeat count of 1. LC-MS resulting files were searched against the Homo sapiens UniProtKB database (UP000005640, 78,120 entries) using the software program Proteome Discoverer (version 2.3.0.523, ThermoFisher, San Jose, CA, USA). Oxidized methionine was set as a dynamic modification and carbamidomethylation of cysteines was set as a fixed modification. The precursor and fragment mass tolerances were 10 ppm and 0.6 ppm, respectively. False discovery rate (FDR) was set to 1% both at protein and peptide level. The identified proteins must contain at least 1 unique peptide sequence. All proteins were then grouped in Master proteins.

LFQ was also performed by Proteome Discoverer. Precursor ion quantification was based on the intensity values, calculated as the average of the 3 most abundant distinct peptides for each protein. To correct for experimental bias, a normalization was performed on the total peptide amount. Lists of the most abundant proteins for all the experiments were obtained by ordering the proteins according to their intensity.

Three different parameters were considered for LFQ: intensity, peak area and PSM. Absolute and normalized values were obtained by Proteome Discoverer.

### 4.8. Process and Pathway Analysis

Pathway analysis was performed by KEGG Mapper, a collection of tools for KEGG mapping [45], and by Database for Annotation, Visualization and Integrated Discovery (DAVID) [46,47]. The web-accessible program PANTHER was used to perform GO classification of the identified proteins [48].

### 4.9. Statistical Analysis

The PSM, peak area and intensity values of the identified proteins were normalized using a total signal normalization method and compared using a LFQ, as previously reported [49]. Specifically, the data matrix dimensionality (*n* total = 24; *n* = 12 per condition) was reduced by LDA. A pairwise comparison was performed and only proteins with F ratio ≥4 and uncorrected *p*-value ≤0.05 were retained. Finally, proteins selected by LDA (here referred to as descriptors) were processed by hierarchical clustering applying the Ward’s method and the Euclidean distance metric. Data processing was performed using JMP 15.1 SAS software.

## Data Availability

Proteomic data are available on the ProteomeXchange Consortium repository at the site: ftp://massive.ucsd.edu/MSV000087703/ (accssed on 10 September 2021).

## Supplementary Materials

The following are available online at https://www.mdpi.com/article/10.3390/ijms221910750/s1
