# Supplementary material for: Single-Tear Proteomics: A Feasible Approach to Precision Medicine
Source: Int J Mol Sci. 2021 Oct 4;22(19):10750. doi: 10.3390/ijms221910750 (PMC8509675; doi:10.3390/ijms221910750)
Supplement: Supplementary file 1 [file ijms-22-10750-s001.zip › ijms-1396135-supplementary.pdf]

Subject ID	Gender	Age
Subject 01	Male	26
Subject 02	Male	25
Subject 03	Female	23
Subject 04	Male	26
Subject 05	Male	23
Subject 06	Male	21
Subject 07	Female	21
Subject 08	Female	19
Subject 09	Female	19
Subject 10	Female	29
Subject 11	Male	25
Subject 12	Male	29
Subject 13	Female	21
Subject 14	Male	25
Subject 15	Male	23
Subject 16	Female	25
Subject 17	Male	25
Subject 18	Male	25
Subject 19	Female	23

Subject 20	Male	23
Subject 21	Female	24
Subject 22	Female	24
Subject 23	Female	22

Supporting information S1. Subject IDs, gender, and age. Subjects 09 and Subject 13, highlighted in red, performed the sample collection in the morning (9-10 AM) and in the afternoon (5-6 PM) on the same day, once a week for three consecutive weeks.

Supporting information S2. List of keratins and keratin-associated proteins, identified over 70 runs.

Accession	Description	calc. pI	MW [kDa]
P04264	Keratin, type II cytoskeletal 1 OS=Homo sapiens OX=9606 GN=KRT1 PE=1 SV=6	8.12	66
P13645	Keratin, type I cytoskeletal 10 OS=Homo sapiens OX=9606 GN=KRT10 PE=1 SV=6	5.21	58.8
P35527	Keratin, type I cytoskeletal 9 OS=Homo sapiens OX=9606 GN=KRT9 PE=1 SV=3	5.24	62
P35908	Keratin, type II cytoskeletal 2 epidermal OS=Homo sapiens OX=9606 GN=KRT2 PE=1 SV=2	8	65.4
P13647	Keratin, type II cytoskeletal 5 OS=Homo sapiens OX=9606 GN=KRT5 PE=1 SV=3	7.74	62.3
P02538	Keratin, type II cytoskeletal 6A OS=Homo sapiens OX=9606 GN=KRT6A PE=1 SV=3	8	60
P04259	Keratin, type II cytoskeletal 6B OS=Homo sapiens OX=9606 GN=KRT6B PE=1 SV=5	8	60
P02533	Keratin, type I cytoskeletal 14 OS=Homo sapiens OX=9606 GN=KRT14 PE=1 SV=4	5.16	51.5
P08779	Keratin, type I cytoskeletal 16 OS=Homo sapiens OX=9606 GN=KRT16 PE=1 SV=4	5.05	51.2
P13646	Keratin, type I cytoskeletal 13 OS=Homo sapiens OX=9606 GN=KRT13 PE=1 SV=4	4.96	49.6
P19013	Keratin, type II cytoskeletal 4 OS=Homo sapiens OX=9606 GN=KRT4 PE=1 SV=5	6.61	56.1
O95678	Keratin, type II cytoskeletal 75 OS=Homo sapiens OX=9606 GN=KRT75 PE=1 SV=2	7.74	59.5
Q01546	Keratin, type II cytoskeletal 2 oral OS=Homo sapiens OX=9606 GN=KRT76 PE=1 SV=2	8.12	65.8
P12035	Keratin, type II cytoskeletal 3 OS=Homo sapiens OX=9606 GN=KRT3 PE=1 SV=3	6.48	64.4
Q04695	Keratin, type I cytoskeletal 17 OS=Homo sapiens OX=9606 GN=KRT17 PE=1 SV=2	5.02	48.1
P19012	Keratin, type I cytoskeletal 15 OS=Homo sapiens OX=9606 GN=KRT15 PE=1 SV=3	4.77	49.2
P08727	Keratin, type I cytoskeletal 19 OS=Homo sapiens OX=9606 GN=KRT19 PE=1 SV=4	5.14	44.1
Q7Z794	Keratin, type II cytoskeletal 1b OS=Homo sapiens OX=9606 GN=KRT77 PE=1 SV=3	5.99	61.9
F8W1S1	Keratin, type II cytoskeletal 74 OS=Homo sapiens OX=9606 GN=KRT74 PE=1 SV=1	7.44	59.4
P05787	Keratin, type II cytoskeletal 8 OS=Homo sapiens OX=9606 GN=KRT8 PE=1 SV=7	5.59	53.7
Q5T749	Keratinocyte proline-rich protein OS=Homo sapiens OX=9606 GN=KPRP PE=1 SV=1	8.27	64.1
O43790	Keratin, type II cuticular Hb6 OS=Homo sapiens OX=9606 GN=KRT86 PE=1 SV=1	5.66	53.5
P78386	Keratin, type II cuticular Hb5 OS=Homo sapiens OX=9606 GN=KRT85 PE=1 SV=1	6.55	55.8
P08729	Keratin, type II cytoskeletal 7 OS=Homo sapiens OX=9606 GN=KRT7 PE=1 SV=5	5.48	51.4
P78385	Keratin, type II cuticular Hb3 OS=Homo sapiens OX=9606 GN=KRT83 PE=1 SV=2	5.64	54.2
Q3SY84	Keratin, type II cytoskeletal 71 OS=Homo sapiens OX=9606 GN=KRT71 PE=1 SV=3	6.61	57.3
Q14533	Keratin, type II cuticular Hb1 OS=Homo sapiens OX=9606 GN=KRT81 PE=1 SV=3	5.47	54.9
Q15323	Keratin, type I cuticular Ha1 OS=Homo sapiens OX=9606 GN=KRT31 PE=1 SV=3	4.88	47.2
Q14525	Keratin, type I cuticular Ha3-II OS=Homo sapiens OX=9606 GN=KRT33B PE=1 SV=3	4.84	46.2
O76009	Keratin, type I cuticular Ha3-I OS=Homo sapiens OX=9606 GN=KRT33A PE=2 SV=2	4.82	45.9
A6NCN2	Putative keratin-87 protein OS=Homo sapiens OX=9606 GN=KRT87P PE=5 SV=4	5.8	29.1
Q8N1N4	Keratin, type II cytoskeletal 78 OS=Homo sapiens OX=9606 GN=KRT78 PE=1 SV=2	6.02	56.8
Q6KB66	Keratin, type II cytoskeletal 80 OS=Homo sapiens OX=9606 GN=KRT80 PE=1 SV=2	5.67	50.5
A0A140TA69	Keratin, type I cuticular Ha4 OS=Homo sapiens OX=9606 GN=KRT34 PE=3 SV=1	5.06	49.4
Q92764	Keratin, type I cuticular Ha5 OS=Homo sapiens OX=9606 GN=KRT35 PE=1 SV=5	4.91	50.3
Q9NSB4	Keratin, type II cuticular Hb2 OS=Homo sapiens OX=9606 GN=KRT82 PE=1 SV=3	6.74	56.6
O76013	Keratin, type I cuticular Ha6 OS=Homo sapiens OX=9606 GN=KRT36 PE=1 SV=1	4.94	52.2
Q14532	Keratin, type I cuticular Ha2 OS=Homo sapiens OX=9606 GN=KRT32 PE=1 SV=3	4.84	50.3
F5H1T9	Keratin-associated protein 2-1 OS=Homo sapiens OX=9606 GN=KRTAP2-1 PE=4 SV=1	7.81	13
Q8IUC1	Keratin-associated protein 11-1 OS=Homo sapiens OX=9606 GN=KRTAP11-1 PE=1 SV=1	7.9	17.1
A0A1B0GVI3	Keratin, type I cytoskeletal 10 (Fragment) OS=Homo sapiens OX=9606 GN=KRT10 PE=1 SV=1	8.44	10
Q9BYR7	Keratin-associated protein 3-2 OS=Homo sapiens OX=9606 GN=KRTAP3-2 PE=1 SV=1	5.69	10.4

Supporting information S3. List of 890 proteins (keratins and keratin-associated proteins were filtered out), identified over 70 runs

,

Accession	Description	calc. pI	MW [kDa]
P02788	Lactotransferrin OS=Homo sapiens OX=9606 GN=LTF PE=1 SV=6	8.12	78.1
P31025	Lipocalin-1 OS=Homo sapiens OX=9606 GN=LCN1 PE=1 SV=1	5.58	19.2
O75556	Mammaglobin-B OS=Homo sapiens OX=9606 GN=SCGB2A1 PE=1 SV=1	5.78	10.9
P61626	Lysozyme C OS=Homo sapiens OX=9606 GN=LYZ PE=1 SV=1	9.16	16.5
P12273	Prolactin-inducible protein OS=Homo sapiens OX=9606 GN=PIP PE=1 SV=1	8.05	16.6
F8VV32	1,4-beta-N-acetylmuramidase C OS=Homo sapiens OX=9606 GN=LYZ PE=1 SV=1	9.07	11.5
A0A0B4J259	1,4-beta-N-acetylmuramidase C OS=Homo sapiens OX=9606 GN=LYZ PE=1 SV=1	8.69	15.3
P01876	Immunoglobulin heavy constant alpha 1 OS=Homo sapiens OX=9606 GN=IGHA1 PE=1 SV=2	6.51	37.6
A0A0G2JMB2	Immunoglobulin heavy constant alpha 2 (Fragment) OS=Homo sapiens OX=9606 GN=IGHA2 PE=1 SV=1	6.1	36.5
P01833	Polymeric immunoglobulin receptor OS=Homo sapiens OX=9606 GN=PIGR PE=1 SV=4	5.74	83.2
P01036	Cystatin-S OS=Homo sapiens OX=9606 GN=CST4 PE=1 SV=3	5.02	16.2
P01877	Immunoglobulin heavy constant alpha 2 OS=Homo sapiens OX=9606 GN=IGHA2 PE=1 SV=4	6.27	36.6
P98160	Basement membrane-specific heparan sulfate proteoglycan core protein OS=Homo sapiens OX=9606 GN=HSPG2 PE=1 SV=4	6.51	468.5
P01834	Immunoglobulin kappa constant OS=Homo sapiens OX=9606 GN=IGKC PE=1 SV=2	6.52	11.8
P02768	Albumin OS=Homo sapiens OX=9606 GN=ALB PE=1 SV=2	6.28	69.3
P25311	Zinc-alpha-2-glycoprotein OS=Homo sapiens OX=9606 GN=AZGP1 PE=1 SV=2	6.05	34.2
P01024	Complement C3 OS=Homo sapiens OX=9606 GN=C3 PE=1 SV=2	6.4	187
O95968	Secretoglobin family 1D member 1 OS=Homo sapiens OX=9606 GN=SCGB1D1 PE=1 SV=1	9.25	9.9
P01037	Cystatin-SN OS=Homo sapiens OX=9606 GN=CST1 PE=1 SV=3	7.21	16.4
C9JEV0	Zinc-alpha-2-glycoprotein OS=Homo sapiens OX=9606 GN=AZGP1 PE=1 SV=1	5.73	26.3
Q99935	Opiorphin prepropeptide OS=Homo sapiens OX=9606 GN=OPRPN PE=1 SV=2	10.42	27.2
A0A0J9YXF8	Proline-rich protein 4 OS=Homo sapiens OX=9606 GN=PRR4 PE=1 SV=1	7.52	16.9
Q9UGM3	Deleted in malignant brain tumors 1 protein OS=Homo sapiens OX=9606 GN=DMBT1 PE=1 SV=2	5.44	260.6
Q9GZZ8	Extracellular glycoprotein lacritin OS=Homo sapiens OX=9606 GN=LACRT PE=1 SV=1	5.5	14.2
P01591	Immunoglobulin J chain OS=Homo sapiens OX=9606 GN=JCHAIN PE=1 SV=4	5.24	18.1
P0DOY3	Immunoglobulin lambda constant 3 OS=Homo sapiens OX=9606 GN=IGLC3 PE=1 SV=1	7.24	11.3
P00450	Ceruloplasmin OS=Homo sapiens OX=9606 GN=CP PE=1 SV=1	5.72	122.1
P22079	Lactoperoxidase OS=Homo sapiens OX=9606 GN=LPO PE=1 SV=2	8.62	80.2
P09228	Cystatin-SA OS=Homo sapiens OX=9606 GN=CST2 PE=1 SV=1	4.93	16.4

E9PFZ2	Ceruloplasmin OS=Homo sapiens OX=9606 GN=CP PE=1 SV=1	5.77	108.8
A0A0B4J231	Immunoglobulin lambda-like polypeptide 5 OS=Homo sapiens OX=9606 GN=IGLL5 PE=1 SV=1	8.84	23.1
P10909	Clusterin OS=Homo sapiens OX=9606 GN=CLU PE=1 SV=1	6.27	52.5
Q13421	Mesothelin OS=Homo sapiens OX=9606 GN=MSLN PE=1 SV=2	6.38	68.9
P01871	Immunoglobulin heavy constant mu OS=Homo sapiens OX=9606 GN=IGHM PE=1 SV=4	6.77	49.4
Q08380	Galectin-3-binding protein OS=Homo sapiens OX=9606 GN=LGALS3BP PE=1 SV=1	5.27	65.3
A0A5H1ZRQ7	Immunoglobulin lambda constant 7 (Fragment) OS=Homo sapiens OX=9606 GN=IGLC7 PE=1 SV=1	8.29	11.3
P07602	Prosaposin OS=Homo sapiens OX=9606 GN=PSAP PE=1 SV=2	5.17	58.1
P06396	Gelsolin OS=Homo sapiens OX=9606 GN=GSN PE=1 SV=1	6.28	85.6
P20061	Transcobalamin-1 OS=Homo sapiens OX=9606 GN=TCN1 PE=1 SV=2	5.03	48.2
A0A3B3IRX2	Phospholipase A2 OS=Homo sapiens OX=9606 GN=PLA2G2A PE=1 SV=1	9.06	13
P55058	Phospholipid transfer protein OS=Homo sapiens OX=9606 GN=PLTP PE=1 SV=1	7.01	54.7
P80303	Nucleobindin-2 OS=Homo sapiens OX=9606 GN=NUCB2 PE=1 SV=3	5.12	50.2
A0A0C4DGN4	Zymogen granule protein 16 homolog B OS=Homo sapiens OX=9606 GN=ZG16B PE=1 SV=1	5.95	19.6
P07858	Cathepsin B OS=Homo sapiens OX=9606 GN=CTSB PE=1 SV=3	6.3	37.8
P00738	Haptoglobin OS=Homo sapiens OX=9606 GN=HP PE=1 SV=1	6.58	45.2
A0A2Q2TTZ9	Immunoglobulin kappa variable 1-33 OS=Homo sapiens OX=9606 GN=IGKV1D-33 PE=1 SV=1	5.34	11.8
P80188	Neutrophil gelatinase-associated lipocalin OS=Homo sapiens OX=9606 GN=LCN2 PE=1 SV=2	8.91	22.6
P02787	Serotransferrin OS=Homo sapiens OX=9606 GN=TF PE=1 SV=3	7.12	77
P03973	Antileukoproteinase OS=Homo sapiens OX=9606 GN=SLPI PE=1 SV=2	8.75	14.3
P60709	Actin, cytoplasmic 1 OS=Homo sapiens OX=9606 GN=ACTB PE=1 SV=1	5.48	41.7
A0A4W8ZXM2	Immunoglobulin heavy variable 3-72 OS=Homo sapiens OX=9606 GN=IGHV3-72 PE=1 SV=1	7.97	11.2
A0A0B4J2B5	Immunoglobulin heavy variable 3/OR16-9 (non-functional) (Fragment) OS=Homo sapiens OX=9606 GN=IGHV3OR16-9 PE=1 SV=1	8.46	10.7
Q8N474	Secreted frizzled-related protein 1 OS=Homo sapiens OX=9606 GN=SFRP1 PE=1 SV=1	8.85	35.4
Q14515	SPARC-like protein 1 OS=Homo sapiens OX=9606 GN=SPARCL1 PE=1 SV=2	4.81	75.2
A0A1B0GVD5	Cathepsin D OS=Homo sapiens OX=9606 GN=CTSD PE=1 SV=1	6.54	44.2
Q14697	Neutral alpha-glucosidase AB OS=Homo sapiens OX=9606 GN=GANAB PE=1 SV=3	6.14	106.8
P01011	Alpha-1-antichymotrypsin OS=Homo sapiens OX=9606 GN=SERPINA3 PE=1 SV=2	5.52	47.6
Q02809	Procollagen-lysine,2-oxoglutarate 5-dioxygenase 1 OS=Homo sapiens OX=9606 GN=PLOD1 PE=1 SV=2	6.95	83.5
Q13217	DnaJ homolog subfamily C member 3 OS=Homo sapiens OX=9606 GN=DNAJC3 PE=1 SV=1	6.15	57.5
H3BMA1	Mesothelin (Fragment) OS=Homo sapiens OX=9606 GN=MSLN PE=1 SV=2	5.97	37.6
O00391	Sulfhydryl oxidase 1 OS=Homo sapiens OX=9606 GN=QSOX1 PE=1 SV=3	8.92	82.5

Q15782	Chitinase-3-like protein 2 OS=Homo sapiens OX=9606 GN=CHI3L2 PE=1 SV=1	7.53	43.5
Q02818	Nucleobindin-1 OS=Homo sapiens OX=9606 GN=NUCB1 PE=1 SV=4	5.25	53.8
P0DUB6	Alpha-amylase 1A OS=Homo sapiens OX=9606 GN=AMY1A PE=1 SV=1	6.93	57.7
P61769	Beta-2-microglobulin OS=Homo sapiens OX=9606 GN=B2M PE=1 SV=1	6.52	13.7
H0YI00	Extracellular glycoprotein lacritin (Fragment) OS=Homo sapiens OX=9606 GN=LACRT PE=1 SV=1	7.18	8.7
P01034	Cystatin-C OS=Homo sapiens OX=9606 GN=CST3 PE=1 SV=1	8.75	15.8
P08571	Monocyte differentiation antigen CD14 OS=Homo sapiens OX=9606 GN=CD14 PE=1 SV=2	6.23	40.1
P68133	Actin, alpha skeletal muscle OS=Homo sapiens OX=9606 GN=ACTA1 PE=1 SV=1	5.39	42
Q8NBJ4	Golgi membrane protein 1 OS=Homo sapiens OX=9606 GN=GOLM1 PE=1 SV=1	4.97	45.3
P01009	Alpha-1-antitrypsin OS=Homo sapiens OX=9606 GN=SERPINA1 PE=1 SV=3	5.59	46.7
P01780	Immunoglobulin heavy variable 3-7 OS=Homo sapiens OX=9606 GN=IGHV3-7 PE=1 SV=2	6.57	12.9
P01619	Immunoglobulin kappa variable 3-20 OS=Homo sapiens OX=9606 GN=IGKV3-20 PE=1 SV=2	4.96	12.5
A0A0J9YXB8	Prosaposin (Fragment) OS=Homo sapiens OX=9606 GN=PSAP PE=1 SV=1	4.88	18.8
Q92743	Serine protease HTRA1 OS=Homo sapiens OX=9606 GN=HTRA1 PE=1 SV=1	7.83	51.3
P04083	Annexin A1 OS=Homo sapiens OX=9606 GN=ANXA1 PE=1 SV=2	7.02	38.7
Q96S96	Phosphatidylethanolamine-binding protein 4 OS=Homo sapiens OX=9606 GN=PEBP4 PE=1 SV=3	6.54	25.7
P01782	Immunoglobulin heavy variable 3-9 OS=Homo sapiens OX=9606 GN=IGHV3-9 PE=1 SV=2	7.08	12.9
A0A024R6I7	Alpha-1-antitrypsin OS=Homo sapiens OX=9606 GN=SERPINA1 PE=1 SV=1	5.59	46.7
A0A0J9YY99	Ig-like domain-containing protein (Fragment) OS=Homo sapiens OX=9606 PE=1 SV=1	8.92	13
P06733	Alpha-enolase OS=Homo sapiens OX=9606 GN=ENO1 PE=1 SV=2	7.39	47.1
A0A5H1ZRS2	Immunoglobulin kappa variable 2D-28 OS=Homo sapiens OX=9606 GN=IGKV2D-28 PE=1 SV=1	5.24	11
P01033	Metalloproteinase inhibitor 1 OS=Homo sapiens OX=9606 GN=TIMP1 PE=1 SV=1	8.1	23.2
A0A0B4J1V1	Immunoglobulin heavy variable 3-21 OS=Homo sapiens OX=9606 GN=IGHV3-21 PE=1 SV=1	8.28	12.8
P01762	Immunoglobulin heavy variable 3-11 OS=Homo sapiens OX=9606 GN=IGHV3-11 PE=1 SV=2	8.6	12.9
P01857	Immunoglobulin heavy constant gamma 1 OS=Homo sapiens OX=9606 GN=IGHG1 PE=1 SV=1	8.19	36.1
P15924	Desmoplakin OS=Homo sapiens OX=9606 GN=DSP PE=1 SV=3	6.81	331.6
P07355	Annexin A2 OS=Homo sapiens OX=9606 GN=ANXA2 PE=1 SV=2	7.75	38.6
Q99574	Neuroserpin OS=Homo sapiens OX=9606 GN=SERPINI1 PE=1 SV=1	4.91	46.4
D6RHI9	Ribonuclease T2 (Fragment) OS=Homo sapiens OX=9606 GN=RNASET2 PE=1 SV=8	6.76	29.1
A0A5F9UP49	45 kDa calcium-binding protein OS=Homo sapiens OX=9606 GN=SDF4 PE=1 SV=1	4.86	40.9

A0A0B4J1X5	Immunoglobulin heavy variable 3-74 OS=Homo sapiens OX=9606 GN=IGHV3-74 PE=3 SV=1	8.66	12.8
P28799	Progranulin OS=Homo sapiens OX=9606 GN=GRN PE=1 SV=2	6.83	63.5
O43490	Prominin-1 OS=Homo sapiens OX=9606 GN=PROM1 PE=1 SV=1	7.27	97.1
P07237	Protein disulfide-isomerase OS=Homo sapiens OX=9606 GN=P4HB PE=1 SV=3	4.87	57.1
P06331	Immunoglobulin heavy variable 4-34 OS=Homo sapiens OX=9606 GN=IGHV4-34 PE=1 SV=2	9.33	13.8
P04406	Glyceraldehyde-3-phosphate dehydrogenase OS=Homo sapiens OX=9606 GN=GAPDH PE=1 SV=3	8.46	36
P01763	Immunoglobulin heavy variable 3-48 OS=Homo sapiens OX=9606 GN=IGHV3-48 PE=1 SV=2	5.36	12.8
P11021	Endoplasmic reticulum chaperone BiP OS=Homo sapiens OX=9606 GN=HSPA5 PE=1 SV=2	5.16	72.3
K7ELL7	Glucosidase 2 subunit beta OS=Homo sapiens OX=9606 GN=PRKCSH PE=1 SV=1	4.41	60.2
P01768	Immunoglobulin heavy variable 3-30 OS=Homo sapiens OX=9606 GN=IGHV3-30 PE=1 SV=2	8.92	12.9
O43852	Calumenin OS=Homo sapiens OX=9606 GN=CALU PE=1 SV=2	4.64	37.1
P01767	Immunoglobulin heavy variable 3-53 OS=Homo sapiens OX=9606 GN=IGHV3-53 PE=1 SV=2	8.28	12.8
P01824	Immunoglobulin heavy variable 4-39 OS=Homo sapiens OX=9606 GN=IGHV4-39 PE=1 SV=2	9.26	13.9
P02750	Leucine-rich alpha-2-glycoprotein OS=Homo sapiens OX=9606 GN=LRG1 PE=1 SV=2	6.95	38.2
A0A5H1ZRS9	Immunoglobulin kappa variable 2D-29 OS=Homo sapiens OX=9606 GN=IGKV2D-29 PE=1 SV=1	5.94	11.2
P06702	Protein S100-A9 OS=Homo sapiens OX=9606 GN=S100A9 PE=1 SV=1	6.13	13.2
P04792	Heat shock protein beta-1 OS=Homo sapiens OX=9606 GN=HSPB1 PE=1 SV=2	6.4	22.8
A0A0C4DH25	Immunoglobulin kappa variable 3D-20 OS=Homo sapiens OX=9606 GN=IGKV3D-20 PE=3 SV=1	4.59	12.5
P12259	Coagulation factor V OS=Homo sapiens OX=9606 GN=F5 PE=1 SV=4	6.05	251.5
A0A0C4DH38	Immunoglobulin heavy variable 5-51 OS=Homo sapiens OX=9606 GN=IGHV5-51 PE=3 SV=1	8.27	12.7
P34096	Ribonuclease 4 OS=Homo sapiens OX=9606 GN=RNASE4 PE=1 SV=3	9.03	16.8
A0A0B4J1V0	Immunoglobulin heavy variable 3-15 OS=Homo sapiens OX=9606 GN=IGHV3-15 PE=3 SV=1	8.62	12.9
H0YLF3	Beta-2-microglobulin (Fragment) OS=Homo sapiens OX=9606 GN=B2M PE=1 SV=1	5.15	8.5
P27487	Dipeptidyl peptidase 4 OS=Homo sapiens OX=9606 GN=DPP4 PE=1 SV=2	6.04	88.2
Q08431	Lactadherin OS=Homo sapiens OX=9606 GN=MFGE8 PE=1 SV=3	8.15	43.1
A0A075B6K4	Immunoglobulin lambda variable 3-10 OS=Homo sapiens OX=9606 GN=IGLV3-10 PE=3 SV=2	4.83	12.4
A0A075B6R2	Immunoglobulin heavy variable 4-4 OS=Homo sapiens OX=9606 GN=IGHV4-4 PE=3 SV=2	9.6	12.8
P01764	Immunoglobulin heavy variable 3-23 OS=Homo sapiens OX=9606 GN=IGHV3-23 PE=1 SV=2	8.28	12.6
A0A0C4DH31	Immunoglobulin heavy variable 1-18 OS=Homo sapiens OX=9606 GN=IGHV1-18 PE=3 SV=1	8.84	12.8

Q6MZM9	Proline-rich protein 27 OS=Homo sapiens OX=9606 GN=PRR27 PE=2 SV=1	4.87	22.7
A0A0J9YXX1	Immunoglobulin heavy variable 5-10-1 OS=Homo sapiens OX=9606 GN=IGHV5-10-1 PE=3 SV=1	8.28	12.8
O14773	Tripeptidyl-peptidase 1 OS=Homo sapiens OX=9606 GN=TPP1 PE=1 SV=2	6.48	61.2
P03950	Angiogenin OS=Homo sapiens OX=9606 GN=ANG PE=1 SV=1	9.64	16.5
A2NJV5	Immunoglobulin kappa variable 2-29 OS=Homo sapiens OX=9606 GN=IGKV2-29 PE=3 SV=2	7.28	13.1
P06312	Immunoglobulin kappa variable 4-1 OS=Homo sapiens OX=9606 GN=IGKV4-1 PE=1 SV=1	5.25	13.4
P09211	Glutathione S-transferase P OS=Homo sapiens OX=9606 GN=GSTP1 PE=1 SV=2	5.64	23.3
P29508	Serpin B3 OS=Homo sapiens OX=9606 GN=SERPINB3 PE=1 SV=2	6.81	44.5
Q13162	Peroxiredoxin-4 OS=Homo sapiens OX=9606 GN=PRDX4 PE=1 SV=1	6.29	30.5
P14923	Junction plakoglobin OS=Homo sapiens OX=9606 GN=JUP PE=1 SV=3	6.14	81.7
A0A087WSY4	Immunoglobulin heavy variable 4-30-2 OS=Homo sapiens OX=9606 GN=IGHV4-30-2 PE=3 SV=1	9.67	13
D6RF35	Gc-globulin OS=Homo sapiens OX=9606 GN=GC PE=1 SV=1	5.52	53
P14618	Pyruvate kinase PKM OS=Homo sapiens OX=9606 GN=PKM PE=1 SV=4	7.84	57.9
A0A075B7B8	Immunoglobulin heavy variable 3/OR16-12 (non-functional) (Fragment) OS=Homo sapiens OX=9606 GN=IGHV3OR16-12 PE=1 SV=1	6.51	12.9
P06310	Immunoglobulin kappa variable 2-30 OS=Homo sapiens OX=9606 GN=IGKV2-30 PE=3 SV=2	8.51	13.2
A0A087WW87	Immunoglobulin kappa variable 2-40 OS=Homo sapiens OX=9606 GN=IGKV2-40 PE=3 SV=2	4.61	13.3
A0A0G2JIW1	Heat shock 70 kDa protein 1B OS=Homo sapiens OX=9606 GN=HSPA1B PE=1 SV=1	5.66	70.1
Q8NCL4	Polypeptide N-acetylgalactosaminyltransferase 6 OS=Homo sapiens OX=9606 GN=GALNT6 PE=2 SV=2	8.18	71.1
Q8WVQ1	Soluble calcium-activated nucleotidase 1 OS=Homo sapiens OX=9606 GN=CANT1 PE=1 SV=1	6.09	44.8
A0A075B6S6	Immunoglobulin kappa variable 2D-30 OS=Homo sapiens OX=9606 GN=IGKV2D-30 PE=3 SV=1	7.99	13.2
A0A075B6K5	Immunoglobulin lambda variable 3-9 OS=Homo sapiens OX=9606 GN=IGLV3-9 PE=3 SV=1	7.39	12.3
P23083	Immunoglobulin heavy variable 1-2 OS=Homo sapiens OX=9606 GN=IGHV1-2 PE=1 SV=2	9.13	13.1
P80748	Immunoglobulin lambda variable 3-21 OS=Homo sapiens OX=9606 GN=IGLV3-21 PE=1 SV=2	5.29	12.4
A0A4W9A917	Immunoglobulin heavy constant gamma 3 (Fragment) OS=Homo sapiens OX=9606 GN=IGHG3 PE=1 SV=1	7.9	41.3
P22352	Glutathione peroxidase 3 OS=Homo sapiens OX=9606 GN=GPX3 PE=1 SV=2	8.13	25.5
A0A0C4DH73	Immunoglobulin kappa variable 1-12 OS=Homo sapiens OX=9606 GN=IGKV1-12 PE=3 SV=1	8.31	12.6
P23284	Peptidyl-prolyl cis-trans isomerase B OS=Homo sapiens OX=9606 GN=PPIB PE=1 SV=2	9.41	23.7
Q14118	Dystroglycan OS=Homo sapiens OX=9606 GN=DAG1 PE=1 SV=2	8.56	97.4
A0A087WT59	Transthyretin OS=Homo sapiens OX=9606 GN=TTR PE=1 SV=2	5.45	12.6

P07686	Beta-hexosaminidase subunit beta OS=Homo sapiens OX=9606 GN=HEXB PE=1 SV=3	6.76	63.1
A0A0B4J1U7	Immunoglobulin heavy variable 6-1 OS=Homo sapiens OX=9606 GN=IGHV6-1 PE=3 SV=1	9.2	13.5
A0A286YFJ8	Immunoglobulin heavy constant gamma 4 (Fragment) OS=Homo sapiens OX=9606 GN=IGHG4 PE=1 SV=1	6.24	43.8
A0A0J9YVY3	Immunoglobulin heavy variable 7-4-1 OS=Homo sapiens OX=9606 GN=IGHV7-4-1 PE=3 SV=1	8.63	12.8
A0A0B4J1X8	Immunoglobulin heavy variable 3-43 OS=Homo sapiens OX=9606 GN=IGHV3-43 PE=3 SV=1	5.41	13.1
P01766	Immunoglobulin heavy variable 3-13 OS=Homo sapiens OX=9606 GN=IGHV3-13 PE=1 SV=2	7.08	12.5
Q86X29	Lipolysis-stimulated lipoprotein receptor OS=Homo sapiens OX=9606 GN=LSR PE=1 SV=4	7.97	71.4
P00751	Complement factor B OS=Homo sapiens OX=9606 GN=CFB PE=1 SV=2	7.06	85.5
P63104	14-3-3 protein zeta/delta OS=Homo sapiens OX=9606 GN=YWHAZ PE=1 SV=1	4.79	27.7
P04433	Immunoglobulin kappa variable 3-11 OS=Homo sapiens OX=9606 GN=IGKV3-11 PE=1 SV=1	4.96	12.6
Q92896	Golgi apparatus protein 1 OS=Homo sapiens OX=9606 GN=GLG1 PE=1 SV=2	6.9	134.5
P30740	Leukocyte elastase inhibitor OS=Homo sapiens OX=9606 GN=SERPINB1 PE=1 SV=1	6.28	42.7
Q9BWS9	Chitinase domain-containing protein 1 OS=Homo sapiens OX=9606 GN=CHID1 PE=1 SV=1	8.63	44.9
Q5D862	Filaggrin-2 OS=Homo sapiens OX=9606 GN=FLG2 PE=1 SV=1	8.31	247.9
P68104	Elongation factor 1-alpha 1 OS=Homo sapiens OX=9606 GN=EEF1A1 PE=1 SV=1	9.01	50.1
P01743	Immunoglobulin heavy variable 1-46 OS=Homo sapiens OX=9606 GN=IGHV1-46 PE=1 SV=2	8.92	12.9
P04432	Immunoglobulin kappa variable 1D-39 OS=Homo sapiens OX=9606 GN=IGKV1D-39 PE=3 SV=2	8.66	12.7
A0A0C4DH67	Immunoglobulin kappa variable 1-8 OS=Homo sapiens OX=9606 GN=IGKV1-8 PE=3 SV=1	9.01	12.5
P49788	Retinoic acid receptor responder protein 1 OS=Homo sapiens OX=9606 GN=RARRES1 PE=1 SV=2	8.51	33.3
P05109	Protein S100-A8 OS=Homo sapiens OX=9606 GN=S100A8 PE=1 SV=1	7.03	10.8
A0A286YEY4	Immunoglobulin heavy constant gamma 2 (Fragment) OS=Homo sapiens OX=9606 GN=IGHG2 PE=1 SV=1	6.52	43.8
O60888	Protein CutA OS=Homo sapiens OX=9606 GN=CUTA PE=1 SV=2	5.5	19.1
Q9Y4L1	Hypoxia up-regulated protein 1 OS=Homo sapiens OX=9606 GN=HYOU1 PE=1 SV=1	5.22	111.3
P60174	Triosephosphate isomerase OS=Homo sapiens OX=9606 GN=TPI1 PE=1 SV=4	6.9	26.7
A0A075B6S5	Immunoglobulin kappa variable 1-27 OS=Homo sapiens OX=9606 GN=IGKV1-27 PE=3 SV=1	8.29	12.7
Q562R1	Beta-actin-like protein 2 OS=Homo sapiens OX=9606 GN=ACTBL2 PE=1 SV=2	5.59	42
P36955	Pigment epithelium-derived factor OS=Homo sapiens OX=9606 GN=SERPINF1 PE=1 SV=4	6.38	46.3

P48594	Serpin B4 OS=Homo sapiens OX=9606 GN=SERPINB4 PE=1 SV=2	6.21	44.8
P19021	Peptidyl-glycine alpha-amidating monooxygenase OS=Homo sapiens OX=9606 GN=PAM PE=1 SV=2	6.42	108.3
Q02413	Desmoglein-1 OS=Homo sapiens OX=9606 GN=DSG1 PE=1 SV=2	5.03	113.7
P02814	Submaxillary gland androgen-regulated protein 3B OS=Homo sapiens OX=9606 GN=SMR3B PE=1 SV=2	9.57	8.2
P01599	Immunoglobulin kappa variable 1-17 OS=Homo sapiens OX=9606 GN=IGKV1-17 PE=1 SV=2	8.68	12.8
O95395	Beta-1,3-galactosyl-O-glycosyl-glycoprotein beta-1,6-N-acetylglucosaminyltransferase 3 OS=Homo sapiens OX=9606 GN=GCNT3 PE=2 SV=1	8.25	50.8
A0A0C4DH32	Immunoglobulin heavy variable 3-20 OS=Homo sapiens OX=9606 GN=IGHV3-20 PE=3 SV=2	7.87	12.7
P07711	Procathepsin L OS=Homo sapiens OX=9606 GN=CTSL PE=1 SV=2	5.45	37.5
P08582	Melanotransferrin OS=Homo sapiens OX=9606 GN=MELTF PE=1 SV=2	5.94	80.2
P07996	Thrombospondin-1 OS=Homo sapiens OX=9606 GN=THBS1 PE=1 SV=2	4.94	129.3
P01701	Immunoglobulin lambda variable 1-51 OS=Homo sapiens OX=9606 GN=IGLV1-51 PE=1 SV=2	7.03	12.2
A0A075B6R9	Probable non-functional immunoglobulin kappa variable 2D-24 OS=Homo sapiens OX=9606 GN=IGKV2D-24 PE=1 SV=1	8.87	13.1
Q9H173	Nucleotide exchange factor SIL1 OS=Homo sapiens OX=9606 GN=SIL1 PE=1 SV=1	5.36	52.1
P43251	Biotinidase OS=Homo sapiens OX=9606 GN=BTD PE=1 SV=2	6.25	61.1
Q15293	Reticulocalbin-1 OS=Homo sapiens OX=9606 GN=RCN1 PE=1 SV=1	5	38.9
Q6UXB2	C-X-C motif chemokine 17 OS=Homo sapiens OX=9606 GN=CXCL17 PE=1 SV=1	10.96	13.8
P01602	Immunoglobulin kappa variable 1-5 OS=Homo sapiens OX=9606 GN=IGKV1-5 PE=1 SV=2	8.28	12.8
A0A1B0GUE3	Acid ceramidase OS=Homo sapiens OX=9606 GN=ASAH1 PE=1 SV=1	6.79	37.4
P53634	Dipeptidyl peptidase 1 OS=Homo sapiens OX=9606 GN=CTSC PE=1 SV=2	6.99	51.8
A0A0B4J2H0	Immunoglobulin heavy variable 1-69D OS=Homo sapiens OX=9606 GN=IGHV1-69D PE=1 SV=1	8.47	12.7
P31944	Caspase-14 OS=Homo sapiens OX=9606 GN=CASP14 PE=1 SV=2	5.58	27.7
P0DP01	Immunoglobulin heavy variable 1-8 OS=Homo sapiens OX=9606 GN=IGHV1-8 PE=3 SV=1	9.17	13
P81605	Dermcidin OS=Homo sapiens OX=9606 GN=DCD PE=1 SV=2	6.54	11.3
A0A0C4DH29	Immunoglobulin heavy variable 1-3 OS=Homo sapiens OX=9606 GN=IGHV1-3 PE=3 SV=1	9.55	13
P08758	Annexin A5 OS=Homo sapiens OX=9606 GN=ANXA5 PE=1 SV=2	5.05	35.9
O43707	Alpha-actinin-4 OS=Homo sapiens OX=9606 GN=ACTN4 PE=1 SV=2	5.44	104.8
O75094	Slit homolog 3 protein OS=Homo sapiens OX=9606 GN=SLIT3 PE=2 SV=3	7.65	167.6
A0A087WSY6	Immunoglobulin kappa variable 3D-15 OS=Homo sapiens OX=9606 GN=IGKV3D-15 PE=3 SV=6	5.19	12.5
P25774	Cathepsin S OS=Homo sapiens OX=9606 GN=CTSS PE=1 SV=3	8.34	37.5
A0A087WU43	Cadherin-1 OS=Homo sapiens OX=9606 GN=CDH1 PE=1 SV=1	4.94	71.2
P15311	Ezrin OS=Homo sapiens OX=9606 GN=EZR PE=1 SV=4	6.27	69.4

A0A075B6H7	Probable non-functional immunoglobulin kappa variable 3-7 OS=Homo sapiens OX=9606 GN=IGKV3-7 PE=1 SV=1	5.25	12.8
A0A087X0S5	Collagen alpha-1(VI) chain OS=Homo sapiens OX=9606 GN=COL6A1 PE=1 SV=1	5.43	108.3
Q9UBT3	Dickkopf-related protein 4 OS=Homo sapiens OX=9606 GN=DKK4 PE=1 SV=1	8.38	24.9
O75888	Tumor necrosis factor ligand superfamily member 13 OS=Homo sapiens OX=9606 GN=TNFSF13 PE=1 SV=1	9.63	27.4
Q01459	Di-N-acetylchitobiase OS=Homo sapiens OX=9606 GN=CTBS PE=1 SV=1	6.64	43.7
P62805	Histone H4 OS=Homo sapiens OX=9606 GN=H4C1 PE=1 SV=2	11.36	11.4
P31947	14-3-3 protein sigma OS=Homo sapiens OX=9606 GN=SFN PE=1 SV=1	4.74	27.8
P02647	Apolipoprotein A-I OS=Homo sapiens OX=9606 GN=APOA1 PE=1 SV=1	5.76	30.8
P68371	Tubulin beta-4B chain OS=Homo sapiens OX=9606 GN=TUBB4B PE=1 SV=1	4.89	49.8
A0A075B6I9	Immunoglobulin lambda variable 7-46 OS=Homo sapiens OX=9606 GN=IGLV7-46 PE=3 SV=4	7.2	12.5
Q5QNW6	Histone H2B type 2-F OS=Homo sapiens OX=9606 GN=H2BC18 PE=1 SV=3	10.32	13.9
P48723	Heat shock 70 kDa protein 13 OS=Homo sapiens OX=9606 GN=HSPA13 PE=1 SV=1	5.76	51.9
P05164	Myeloperoxidase OS=Homo sapiens OX=9606 GN=MPO PE=1 SV=1	8.97	83.8
Q06830	Peroxiredoxin-1 OS=Homo sapiens OX=9606 GN=PRDX1 PE=1 SV=1	8.13	22.1
Q86YZ3	Hornerin OS=Homo sapiens OX=9606 GN=HRNR PE=1 SV=2	10.04	282.2
A8MTF8	Protein FAM3B OS=Homo sapiens OX=9606 GN=FAM3B PE=1 SV=2	8.73	28.3
P08603	Complement factor H OS=Homo sapiens OX=9606 GN=CFH PE=1 SV=4	6.61	139
Q11201	CMP-N-acetylneuraminate-beta-galactosamide-alpha-2,3-sialyltransferase 1 OS=Homo sapiens OX=9606 GN=ST3GAL1 PE=2 SV=1	9.09	39.1
Q16651	Prostasin OS=Homo sapiens OX=9606 GN=PRSS8 PE=1 SV=1	5.85	36.4
A0A075B6Q5	Immunoglobulin heavy variable 3-64 OS=Homo sapiens OX=9606 GN=IGHV3-64 PE=3 SV=1	7.85	12.9
P05090	Apolipoprotein D OS=Homo sapiens OX=9606 GN=APOD PE=1 SV=1	5.15	21.3
Q9NZ08	Endoplasmic reticulum aminopeptidase 1 OS=Homo sapiens OX=9606 GN=ERAP1 PE=1 SV=3	6.46	107.2
P28325	Cystatin-D OS=Homo sapiens OX=9606 GN=CST5 PE=1 SV=1	7.17	16.1
P06865	Beta-hexosaminidase subunit alpha OS=Homo sapiens OX=9606 GN=HEXA PE=1 SV=2	5.16	60.7
P04211	Immunoglobulin lambda variable 7-43 OS=Homo sapiens OX=9606 GN=IGLV7-43 PE=3 SV=2	7.03	12.4
Q7Z7M9	Polypeptide N-acetylgalactosaminyltransferase 5 OS=Homo sapiens OX=9606 GN=GALNT5 PE=1 SV=1	9.47	106.2
P00338	L-lactate dehydrogenase A chain OS=Homo sapiens OX=9606 GN=LDHA PE=1 SV=2	8.27	36.7
P11717	Cation-independent mannose-6-phosphate receptor OS=Homo sapiens OX=9606 GN=IGF2R PE=1 SV=3	5.94	274.2
A0A0C4DH34	Immunoglobulin heavy variable 4-28 OS=Homo sapiens OX=9606 GN=IGHV4-28 PE=3 SV=1	9.29	13.1
P01709	Immunoglobulin lambda variable 2-8 OS=Homo sapiens OX=9606 GN=IGLV2-8 PE=1 SV=2	5.91	12.4

P01714	Immunoglobulin lambda variable 3-19 OS=Homo sapiens OX=9606 GN=IGLV3-19 PE=1 SV=2	4.96	12
P04430	Immunoglobulin kappa variable 1-16 OS=Homo sapiens OX=9606 GN=IGKV1-16 PE=1 SV=2	8.16	12.6
P59665	Neutrophil defensin 1 OS=Homo sapiens OX=9606 GN=DEFA1 PE=1 SV=1	6.99	10.2
O00462	Beta-mannosidase OS=Homo sapiens OX=9606 GN=MANBA PE=1 SV=3	5.52	100.8
E9PKE3	Heat shock cognate 71 kDa protein OS=Homo sapiens OX=9606 GN=HSPA8 PE=1 SV=1	5.52	68.8
Q86UD1	Out at first protein homolog OS=Homo sapiens OX=9606 GN=OAF PE=2 SV=1	6.84	30.7
P00558	Phosphoglycerate kinase 1 OS=Homo sapiens OX=9606 GN=PGK1 PE=1 SV=3	8.1	44.6
P01717	Immunoglobulin lambda variable 3-25 OS=Homo sapiens OX=9606 GN=IGLV3-25 PE=1 SV=2	4.5	12
P07437	Tubulin beta chain OS=Homo sapiens OX=9606 GN=TUBB PE=1 SV=2	4.89	49.6
O75882	Attractin OS=Homo sapiens OX=9606 GN=ATRN PE=1 SV=2	7.31	158.4
P04350	Tubulin beta-4A chain OS=Homo sapiens OX=9606 GN=TUBB4A PE=1 SV=2	4.88	49.6
Q13885	Tubulin beta-2A chain OS=Homo sapiens OX=9606 GN=TUBB2A PE=1 SV=1	4.89	49.9
P30838	Aldehyde dehydrogenase, dimeric NADP-preferring OS=Homo sapiens OX=9606 GN=ALDH3A1 PE=1 SV=3	6.54	50.4
G3V3E8	Epididymal secretory protein E1 OS=Homo sapiens OX=9606 GN=NPC2 PE=1 SV=1	8.44	19.2
P01700	Immunoglobulin lambda variable 1-47 OS=Homo sapiens OX=9606 GN=IGLV1-47 PE=1 SV=2	5.91	12.3
Q86SF2	N-acetylgalactosaminyltransferase 7 OS=Homo sapiens OX=9606 GN=GALNT7 PE=1 SV=1	7.11	75.3
O00299	Chloride intracellular channel protein 1 OS=Homo sapiens OX=9606 GN=CLIC1 PE=1 SV=4	5.17	26.9
P12814	Alpha-actinin-1 OS=Homo sapiens OX=9606 GN=ACTN1 PE=1 SV=2	5.41	103
Q9UBR2	Cathepsin Z OS=Homo sapiens OX=9606 GN=CTSZ PE=1 SV=1	7.11	33.8
Q04609	Glutamate carboxypeptidase 2 OS=Homo sapiens OX=9606 GN=FOLH1 PE=1 SV=1	6.98	84.3
P36952	Serpin B5 OS=Homo sapiens OX=9606 GN=SERPINB5 PE=1 SV=2	6.05	42.1
A0A0B4J1V2	Immunoglobulin heavy variable 2-26 OS=Homo sapiens OX=9606 GN=IGHV2-26 PE=3 SV=1	8.29	13.2
Q8NES3	Beta-1,3-N-acetylglucosaminyltransferase lunatic fringe OS=Homo sapiens OX=9606 GN=LFNG PE=1 SV=2	9.17	41.7
A0A286YFF7	Palmitoyl-protein hydrolase 1 OS=Homo sapiens OX=9606 GN=PPT1 PE=1 SV=1	6.52	37.1
P62937	Peptidyl-prolyl cis-trans isomerase A OS=Homo sapiens OX=9606 GN=PPIA PE=1 SV=2	7.81	18
E9PK54	Heat shock cognate 71 kDa protein (Fragment) OS=Homo sapiens OX=9606 GN=HSPA8 PE=1 SV=8	7.33	19.9
P15144	Aminopeptidase N OS=Homo sapiens OX=9606 GN=ANPEP PE=1 SV=4	5.48	109.5

A0A0A0MS15	Immunoglobulin heavy variable 3-49 OS=Homo sapiens OX=9606 GN=IGHV3-49 PE=3 SV=1	8.62	13
P31151	Protein S100-A7 OS=Homo sapiens OX=9606 GN=S100A7 PE=1 SV=4	6.77	11.5
Q06828	Fibromodulin OS=Homo sapiens OX=9606 GN=FMOD PE=1 SV=2	6.04	43.2
P31946	14-3-3 protein beta/alpha OS=Homo sapiens OX=9606 GN=YWHAB PE=1 SV=3	4.83	28.1
A0A0C4DH55	Immunoglobulin kappa variable 3D-7 OS=Homo sapiens OX=9606 GN=IGKV3D-7 PE=3 SV=5	5.94	13.1
Q9UNW1	Multiple inositol polyphosphate phosphatase 1 OS=Homo sapiens OX=9606 GN=MINPP1 PE=1 SV=1	7.81	55
P62258	14-3-3 protein epsilon OS=Homo sapiens OX=9606 GN=YWHAE PE=1 SV=1	4.74	29.2
F5H5D3	Tubulin alpha chain OS=Homo sapiens OX=9606 GN=TUBA1C PE=1 SV=1	5.07	57.7
P15907	Beta-galactoside alpha-2,6-sialyltransferase 1 OS=Homo sapiens OX=9606 GN=ST6GAL1 PE=1 SV=1	9.01	46.6
P30101	Protein disulfide-isomerase A3 OS=Homo sapiens OX=9606 GN=PDIA3 PE=1 SV=4	6.35	56.7
P10599	Thioredoxin OS=Homo sapiens OX=9606 GN=TXN PE=1 SV=3	4.92	11.7
A0A075B7D0	Immunoglobulin heavy variable 1/OR15-1 (non-functional) (Fragment) OS=Homo sapiens OX=9606 GN=IGHV1OR15-1 PE=1 SV=1	9.13	13
P26038	Moesin OS=Homo sapiens OX=9606 GN=MSN PE=1 SV=3	6.4	67.8
J3QTR3	Ubiquitin-40S ribosomal protein S27a (Fragment) OS=Homo sapiens OX=9606 GN=RPS27A PE=1 SV=1	10.14	12.2
P26447	Protein S100-A4 OS=Homo sapiens OX=9606 GN=S100A4 PE=1 SV=1	6.11	11.7
Q99519	Sialidase-1 OS=Homo sapiens OX=9606 GN=NEU1 PE=1 SV=1	5.88	45.4
A0A0C4DH35	Probable non-functional immunoglobulin heavy variable 3-35 OS=Homo sapiens OX=9606 GN=IGHV3-35 PE=1 SV=1	7.88	12.8
A0A1B0GVW0	ATPase H(+)-transporting lysosomal accessory protein 2 OS=Homo sapiens OX=9606 GN=ATP6AP2 PE=1 SV=1	5.54	38.4
G5EA09	Syndecan binding protein (Syntenin), isoform CRA_a OS=Homo sapiens OX=9606 GN=SDCBP PE=1 SV=1	8.51	34.8
P04004	Vitronectin OS=Homo sapiens OX=9606 GN=VTN PE=1 SV=1	5.8	54.3
P20930	Filaggrin OS=Homo sapiens OX=9606 GN=FLG PE=1 SV=3	9.25	434.9
P15291	Beta-1,4-galactosyltransferase 1 OS=Homo sapiens OX=9606 GN=B4GALT1 PE=1 SV=5	8.65	43.9
Q01469	Fatty acid-binding protein 5 OS=Homo sapiens OX=9606 GN=FABP5 PE=1 SV=3	7.01	15.2
P22531	Small proline-rich protein 2E OS=Homo sapiens OX=9606 GN=SPRR2E PE=2 SV=2	8.31	7.9
Q9UJJ9	N-acetylglucosamine-1-phosphotransferase subunit gamma OS=Homo sapiens OX=9606 GN=GNPTG PE=1 SV=1	6.95	34
P31949	Protein S100-A11 OS=Homo sapiens OX=9606 GN=S100A11 PE=1 SV=2	7.12	11.7
Q6PCB0	von Willebrand factor A domain-containing protein 1 OS=Homo sapiens OX=9606 GN=VWA1 PE=1 SV=1	7.68	46.8
Q8NBJ7	Inactive C-alpha-formylglycine-generating enzyme 2 OS=Homo sapiens OX=9606 GN=SUMF2 PE=1 SV=2	8	33.8
A0A0B4J1V6	Immunoglobulin heavy variable 3-73 OS=Homo sapiens OX=9606 GN=IGHV3-73 PE=3 SV=1	9.17	12.8
E9PK25	Cofilin, non-muscle isoform OS=Homo sapiens OX=9606 GN=CFL1 PE=1 SV=1	8.34	22.7

P04066	Tissue alpha-L-fucosidase OS=Homo sapiens OX=9606 GN=FUCA1 PE=1 SV=4	6.84	53.7
P01706	Immunoglobulin lambda variable 2-11 OS=Homo sapiens OX=9606 GN=IGLV2-11 PE=1 SV=2	7.24	12.6
P52209	6-phosphogluconate dehydrogenase, decarboxylating OS=Homo sapiens OX=9606 GN=PGD PE=1 SV=3	7.23	53.1
P17931	Galectin-3 OS=Homo sapiens OX=9606 GN=LGALS3 PE=1 SV=5	8.56	26.1
Q14393	Growth arrest-specific protein 6 OS=Homo sapiens OX=9606 GN=GAS6 PE=1 SV=3	5.69	74.9
Q7L7L0	Histone H2A type 3 OS=Homo sapiens OX=9606 GN=H2AW PE=1 SV=3	11.05	14.1
P04075	Fructose-bisphosphate aldolase A OS=Homo sapiens OX=9606 GN=ALDOA PE=1 SV=2	8.09	39.4
P01715	Immunoglobulin lambda variable 3-1 OS=Homo sapiens OX=9606 GN=IGLV3-1 PE=1 SV=2	4.94	12.3
Q13438	Protein OS-9 OS=Homo sapiens OX=9606 GN=OS9 PE=1 SV=1	4.87	75.5
A0A0J9YVU5	Immunoglobulin heavy variable 2-70 (Fragment) OS=Homo sapiens OX=9606 GN=IGHV2-70 PE=1 SV=1	8.48	13.4
P61981	14-3-3 protein gamma OS=Homo sapiens OX=9606 GN=YWHAG PE=1 SV=2	4.89	28.3
P01721	Immunoglobulin lambda variable 6-57 OS=Homo sapiens OX=9606 GN=IGLV6-57 PE=1 SV=2	4.78	12.6
P01023	Alpha-2-macroglobulin OS=Homo sapiens OX=9606 GN=A2M PE=1 SV=3	6.46	163.2
A0A0J9YX35	Immunoglobulin heavy variable 3-64D OS=Homo sapiens OX=9606 GN=IGHV3-64D PE=3 SV=1	7.85	12.8
P07737	Profilin-1 OS=Homo sapiens OX=9606 GN=PFN1 PE=1 SV=2	8.27	15
P32119	Peroxiredoxin-2 OS=Homo sapiens OX=9606 GN=PRDX2 PE=1 SV=5	5.97	21.9
P68366	Tubulin alpha-4A chain OS=Homo sapiens OX=9606 GN=TUBA4A PE=1 SV=1	5.06	49.9
O43505	Beta-1,4-glucuronyltransferase 1 OS=Homo sapiens OX=9606 GN=B4GAT1 PE=1 SV=1	7.2	47.1
A0A075B7D8	Immunoglobulin heavy variable 3/OR15-7 (pseudogene) (Fragment) OS=Homo sapiens OX=9606 GN=IGHV3OR15-7 PE=1 SV=1	8.28	13.1
P27348	14-3-3 protein theta OS=Homo sapiens OX=9606 GN=YWHAQ PE=1 SV=1	4.78	27.7
Q13232	Nucleoside diphosphate kinase 3 OS=Homo sapiens OX=9606 GN=NME3 PE=1 SV=2	7.84	19
A0A0C4DH24	Immunoglobulin kappa variable 6-21 OS=Homo sapiens OX=9606 GN=IGKV6-21 PE=3 SV=1	7.28	12.4
P05186	Alkaline phosphatase, tissue-nonspecific isozyme OS=Homo sapiens OX=9606 GN=ALPL PE=1 SV=4	6.67	57.3
P35579	Myosin-9 OS=Homo sapiens OX=9606 GN=MYH9 PE=1 SV=4	5.6	226.4
Q08188	Protein-glutamine gamma-glutamyltransferase E OS=Homo sapiens OX=9606 GN=TGM3 PE=1 SV=4	5.86	76.6
P98088	Mucin-5AC OS=Homo sapiens OX=9606 GN=MUC5AC PE=1 SV=4	7.02	585.2
O95841	Angiopoietin-related protein 1 OS=Homo sapiens OX=9606 GN=ANGPTL1 PE=2 SV=1	8.21	56.7
P22528	Cornifin-B OS=Homo sapiens OX=9606 GN=SPRR1B PE=1 SV=2	8.48	9.9

Q04917	14-3-3 protein eta OS=Homo sapiens OX=9606 GN=YWHAH PE=1 SV=4	4.84	28.2
P00352	Retinal dehydrogenase 1 OS=Homo sapiens OX=9606 GN=ALDH1A1 PE=1 SV=2	6.73	54.8
P09668	Pro-cathepsin H OS=Homo sapiens OX=9606 GN=CTSH PE=1 SV=4	8.07	37.4
Q96DR8	Mucin-like protein 1 OS=Homo sapiens OX=9606 GN=MUCL1 PE=1 SV=1	4.64	9
Q9HAT2	Sialate O-acetylesterase OS=Homo sapiens OX=9606 GN=SIAE PE=1 SV=1	7.33	58.3
Q9BUF5	Tubulin beta-6 chain OS=Homo sapiens OX=9606 GN=TUBB6 PE=1 SV=1	4.88	49.8
Q9NRA1	Platelet-derived growth factor C OS=Homo sapiens OX=9606 GN=PDGFC PE=1 SV=2	6.14	39
P37802	Transgelin-2 OS=Homo sapiens OX=9606 GN=TAGLN2 PE=1 SV=3	8.25	22.4
P30044	Peroxiredoxin-5, mitochondrial OS=Homo sapiens OX=9606 GN=PRDX5 PE=1 SV=4	8.7	22.1
P45877	Peptidyl-prolyl cis-trans isomerase C OS=Homo sapiens OX=9606 GN=PPIC PE=1 SV=1	8.4	22.7
O75976	Carboxypeptidase D OS=Homo sapiens OX=9606 GN=CPD PE=1 SV=2	6.05	152.8
Q9UBC9	Small proline-rich protein 3 OS=Homo sapiens OX=9606 GN=SPRR3 PE=1 SV=2	8.57	18.1
P13796	Plastin-2 OS=Homo sapiens OX=9606 GN=LCP1 PE=1 SV=6	5.43	70.2
P01703	Immunoglobulin lambda variable 1-40 OS=Homo sapiens OX=9606 GN=IGLV1-40 PE=1 SV=2	5.74	12.3
A0A5K1VW95	Malate dehydrogenase OS=Homo sapiens OX=9606 GN=MDH1 PE=1 SV=1	7.3	38.6
P13639	Elongation factor 2 OS=Homo sapiens OX=9606 GN=EEF2 PE=1 SV=4	6.83	95.3
P04632	Calpain small subunit 1 OS=Homo sapiens OX=9606 GN=CAPNS1 PE=1 SV=1	5.2	28.3
A0A075B6J9	Immunoglobulin lambda variable 2-18 OS=Homo sapiens OX=9606 GN=IGLV2-18 PE=3 SV=2	4.82	12.4
Q9UBX1	Cathepsin F OS=Homo sapiens OX=9606 GN=CTSF PE=1 SV=1	8.22	53.3
Q99954	Submaxillary gland androgen-regulated protein 3A OS=Homo sapiens OX=9606 GN=SMR3A PE=1 SV=2	9.57	14
B7Z6Z4	Myosin light polypeptide 6 OS=Homo sapiens OX=9606 GN=MYL6 PE=1 SV=1	5.08	26.7
A6QRJ1	V-type proton ATPase subunit S1 (Fragment) OS=Homo sapiens OX=9606 GN=ATP6AP1 PE=1 SV=1	5.08	24.9
Q96BQ1	Protein FAM3D OS=Homo sapiens OX=9606 GN=FAM3D PE=1 SV=1	9.33	24.9
Q08554	Desmocollin-1 OS=Homo sapiens OX=9606 GN=DSC1 PE=1 SV=2	5.43	99.9
A0A0B4J2D9	Immunoglobulin kappa variable 1D-13 OS=Homo sapiens OX=9606 GN=IGKV1D-13 PE=3 SV=1	7.84	12.6
P15814	Immunoglobulin lambda-like polypeptide 1 OS=Homo sapiens OX=9606 GN=IGLL1 PE=1 SV=1	10.07	22.9
P14625	Endoplasmin OS=Homo sapiens OX=9606 GN=HSP90B1 PE=1 SV=1	4.84	92.4
Q9Y646	Carboxypeptidase Q OS=Homo sapiens OX=9606 GN=CPQ PE=1 SV=1	6.18	51.9
B1AP13	Complement decay-accelerating factor OS=Homo sapiens OX=9606 GN=CD55 PE=1 SV=1	8.82	49.3
O43405	Cochlin OS=Homo sapiens OX=9606 GN=COCH PE=1 SV=1	7.96	59.4
P49257	Protein ERGIC-53 OS=Homo sapiens OX=9606 GN=LMAN1 PE=1 SV=2	6.77	57.5
P35321	Cornifin-A OS=Homo sapiens OX=9606 GN=SPRR1A PE=1 SV=2	8.48	9.9
P05089	Arginase-1 OS=Homo sapiens OX=9606 GN=ARG1 PE=1 SV=2	7.21	34.7

O00592	Podocalyxin OS=Homo sapiens OX=9606 GN=PODXL PE=1 SV=2	5.49	58.6
Q8N612	FTS and Hook-interacting protein OS=Homo sapiens OX=9606 GN=FAM160A2 PE=1 SV=3	6.77	105.5
Q9GZM7	Tubulointerstitial nephritis antigen-like OS=Homo sapiens OX=9606 GN=TINAGL1 PE=1 SV=1	6.99	52.4
P01008	Antithrombin-III OS=Homo sapiens OX=9606 GN=SERPINC1 PE=1 SV=1	6.71	52.6
Q99538	Legumain OS=Homo sapiens OX=9606 GN=LGMN PE=1 SV=1	6.55	49.4
C9JEU5	Fibrinogen gamma chain OS=Homo sapiens OX=9606 GN=FGG PE=1 SV=1	6.09	50.3
Q96P63	Serpin B12 OS=Homo sapiens OX=9606 GN=SERPINB12 PE=1 SV=1	5.53	46.2
Q86SQ4	Adhesion G-protein coupled receptor G6 OS=Homo sapiens OX=9606 GN=ADGRG6 PE=1 SV=3	7.87	136.6
P17900	Ganglioside GM2 activator OS=Homo sapiens OX=9606 GN=GM2A PE=1 SV=4	5.31	20.8
P40394	All-trans-retinol dehydrogenase [NAD(+)] ADH7 OS=Homo sapiens OX=9606 GN=ADH7 PE=1 SV=2	7.85	41.5
Q8WUM4	Programmed cell death 6-interacting protein OS=Homo sapiens OX=9606 GN=PDCD6IP PE=1 SV=1	6.52	96
A0A0B4J1Y8	Immunoglobulin lambda variable 9-49 OS=Homo sapiens OX=9606 GN=IGLV9-49 PE=1 SV=1	7.28	13
A0A0A0MS14	Immunoglobulin heavy variable 1-45 OS=Homo sapiens OX=9606 GN=IGHV1-45 PE=3 SV=1	9.1	13.5
P21926	CD9 antigen OS=Homo sapiens OX=9606 GN=CD9 PE=1 SV=4	7.15	25.4
P47929	Galectin-7 OS=Homo sapiens OX=9606 GN=LGALS7 PE=1 SV=2	7.62	15.1
H0YCV9	CD44 antigen (Fragment) OS=Homo sapiens OX=9606 GN=CD44 PE=1 SV=2	5.3	30.6
Q6ZVX7	F-box only protein 50 OS=Homo sapiens OX=9606 GN=NCCRP1 PE=1 SV=1	6.62	30.8
Q7Z406	Myosin-14 OS=Homo sapiens OX=9606 GN=MYH14 PE=1 SV=2	5.6	227.7
P29401	Transketolase OS=Homo sapiens OX=9606 GN=TKT PE=1 SV=3	7.66	67.8
Q14508	WAP four-disulfide core domain protein 2 OS=Homo sapiens OX=9606 GN=WFDC2 PE=1 SV=2	4.84	13
P30086	Phosphatidylethanolamine-binding protein 1 OS=Homo sapiens OX=9606 GN=PEBP1 PE=1 SV=3	7.53	21
Q9UBS3	DnaJ homolog subfamily B member 9 OS=Homo sapiens OX=9606 GN=DNAJB9 PE=1 SV=1	8.27	25.5
P07900	Heat shock protein HSP 90-alpha OS=Homo sapiens OX=9606 GN=HSP90AA1 PE=1 SV=5	5.02	84.6
Q13835	Plakophilin-1 OS=Homo sapiens OX=9606 GN=PKP1 PE=1 SV=2	9.13	82.8
P02675	Fibrinogen beta chain OS=Homo sapiens OX=9606 GN=FGB PE=1 SV=2	8.27	55.9
P01718	Immunoglobulin lambda variable 3-27 OS=Homo sapiens OX=9606 GN=IGLV3-27 PE=1 SV=2	5.01	12.2
P02749	Beta-2-glycoprotein 1 OS=Homo sapiens OX=9606 GN=APOH PE=1 SV=3	7.97	38.3
P02763	Alpha-1-acid glycoprotein 1 OS=Homo sapiens OX=9606 GN=ORM1 PE=1 SV=1	5.02	23.5
P02790	Hemopexin OS=Homo sapiens OX=9606 GN=HPX PE=1 SV=2	7.02	51.6
A0A0A0MSQ0	Plastin-3 OS=Homo sapiens OX=9606 GN=PLS3 PE=1 SV=1	5.94	69.3
A0A0C4DH33	Immunoglobulin heavy variable 1-24 OS=Homo sapiens OX=9606 GN=IGHV1-24 PE=3 SV=1	5.16	12.8
Q9UBG3	Cornulin OS=Homo sapiens OX=9606 GN=CRNN PE=1 SV=1	6.1	53.5

O75223	Gamma-glutamylcyclotransferase OS=Homo sapiens OX=9606 GN=GGCT PE=1 SV=1	5.14	21
A0A0G2JSC0	Immunoglobulin lambda variable 5-45 (Fragment) OS=Homo sapiens OX=9606 GN=IGLV5-45 PE=1 SV=1	7.87	13.2
Q9NQ84	G-protein coupled receptor family C group 5 member C OS=Homo sapiens OX=9606 GN=GPRC5C PE=1 SV=2	8.43	48.2
A0A075B7B6	Immunoglobulin heavy variable 4/OR15-8 (non-functional) (Fragment) OS=Homo sapiens OX=9606 GN=IGHV4OR15-8 PE=4 SV=1	9.6	13
Q16706	Alpha-mannosidase 2 OS=Homo sapiens OX=9606 GN=MAN2A1 PE=1 SV=2	7.58	131.1
P55064	Aquaporin-5 OS=Homo sapiens OX=9606 GN=AQP5 PE=1 SV=1	8.62	28.3
O75503	Ceroid-lipofuscinosis neuronal protein 5 OS=Homo sapiens OX=9606 GN=CLN5 PE=1 SV=2	7.4	41.5
Q9NZT1	Calmodulin-like protein 5 OS=Homo sapiens OX=9606 GN=CALML5 PE=1 SV=2	4.44	15.9
J3KSD8	Bleomycin hydrolase (Fragment) OS=Homo sapiens OX=9606 GN=BLMH PE=1 SV=8	7.46	30.2
O60911	Cathepsin L2 OS=Homo sapiens OX=9606 GN=CTSV PE=1 SV=2	8.76	37.3
P30041	Peroxiredoxin-6 OS=Homo sapiens OX=9606 GN=PRDX6 PE=1 SV=3	6.38	25
P16035	Metalloproteinase inhibitor 2 OS=Homo sapiens OX=9606 GN=TIMP2 PE=1 SV=2	7.49	24.4
Q14764	Major vault protein OS=Homo sapiens OX=9606 GN=MVP PE=1 SV=4	5.48	99.3
P18669	Phosphoglycerate mutase 1 OS=Homo sapiens OX=9606 GN=PGAM1 PE=1 SV=2	7.18	28.8
A0A0A0MT36	Immunoglobulin kappa variable 6D-21 OS=Homo sapiens OX=9606 GN=IGKV6D-21 PE=3 SV=1	7.28	12.3
P15289	Arylsulfatase A OS=Homo sapiens OX=9606 GN=ARSA PE=1 SV=3	6.07	53.6
H0Y4S8	Dyslexia-associated protein KIAA0319-like protein (Fragment) OS=Homo sapiens OX=9606 GN=KIAA0319L PE=1 SV=1	5.66	33.1
P02671	Fibrinogen alpha chain OS=Homo sapiens OX=9606 GN=FGA PE=1 SV=2	6.01	94.9
Q6UWP8	Suprabasin OS=Homo sapiens OX=9606 GN=SBSN PE=1 SV=2	7.01	60.5
P01601	Immunoglobulin kappa variable 1D-16 OS=Homo sapiens OX=9606 GN=IGKV1D-16 PE=3 SV=2	7.74	12.7
P01704	Immunoglobulin lambda variable 2-14 OS=Homo sapiens OX=9606 GN=IGLV2-14 PE=1 SV=2	6.49	12.6
H7BZJ3	Protein disulfide-isomerase A3 (Fragment) OS=Homo sapiens OX=9606 GN=PDIA3 PE=1 SV=1	7.3	13.5
P06737	Glycogen phosphorylase, liver form OS=Homo sapiens OX=9606 GN=PYGL PE=1 SV=4	7.17	97.1
P05155	Plasma protease C1 inhibitor OS=Homo sapiens OX=9606 GN=SERPING1 PE=1 SV=2	6.55	55.1
P04217	Alpha-1B-glycoprotein OS=Homo sapiens OX=9606 GN=A1BG PE=1 SV=4	5.86	54.2
A0A075B6I0	Immunoglobulin lambda variable 8-61 OS=Homo sapiens OX=9606 GN=IGLV8-61 PE=3 SV=7	4.55	12.8
P22314	Ubiquitin-like modifier-activating enzyme 1 OS=Homo sapiens OX=9606 GN=UBA1 PE=1 SV=3	5.76	117.8
P43234	Cathepsin O OS=Homo sapiens OX=9606 GN=CTSO PE=2 SV=1	7.42	35.9
Q10472	Polypeptide N-acetylgalactosaminyltransferase 1 OS=Homo sapiens OX=9606 GN=GALNT1 PE=1 SV=1	7.72	64.2

A0A0G2JRQ6	Ig-like domain-containing protein (Fragment) OS=Homo sapiens OX=9606 PE=1 SV=4	8.29	12.7
Q13449	Limbic system-associated membrane protein OS=Homo sapiens OX=9606 GN=LSAMP PE=1 SV=2	6.98	37.4
O95967	EGF-containing fibulin-like extracellular matrix protein 2 OS=Homo sapiens OX=9606 GN=EFEMP2 PE=1 SV=3	4.94	49.4
Q96EU7	C1GALT1-specific chaperone 1 OS=Homo sapiens OX=9606 GN=C1GALT1C1 PE=1 SV=1	6.84	36.4
A0A0A0MTJ2	Sparc/osteonectin, cwcv and kazal-like domains proteoglycan (Testican) 3, isoform CRA_b OS=Homo sapiens OX=9606 GN=SPOCK3 PE=1 SV=1	5.02	39.4
A0A2U3TZL5	CD59 glycoprotein (Fragment) OS=Homo sapiens OX=9606 GN=CD59 PE=1 SV=1	6.25	13.3
O75874	Isocitrate dehydrogenase [NADP] cytoplasmic OS=Homo sapiens OX=9606 GN=IDH1 PE=1 SV=2	7.01	46.6
I3L116	Brain-specific serine protease 4 OS=Homo sapiens OX=9606 GN=PRSS22 PE=1 SV=1	7.31	21.6
P50395	Rab GDP dissociation inhibitor beta OS=Homo sapiens OX=9606 GN=GDI2 PE=1 SV=2	6.47	50.6
P15309	Prostatic acid phosphatase OS=Homo sapiens OX=9606 GN=ACP3 PE=1 SV=3	6.24	44.5
P04040	Catalase OS=Homo sapiens OX=9606 GN=CAT PE=1 SV=3	7.39	59.7
D6RA82	Annexin OS=Homo sapiens OX=9606 GN=ANXA3 PE=1 SV=1	5.94	32.1
P00390	Glutathione reductase, mitochondrial OS=Homo sapiens OX=9606 GN=GSR PE=1 SV=2	8.5	56.2
P06744	Glucose-6-phosphate isomerase OS=Homo sapiens OX=9606 GN=GPI PE=1 SV=4	8.32	63.1
K7EKI8	Periplakin OS=Homo sapiens OX=9606 GN=PPL PE=1 SV=1	5.62	204.4
P31941	DNA dC->dU-editing enzyme APOBEC-3A OS=Homo sapiens OX=9606 GN=APOBEC3A PE=1 SV=3	6.84	23
P40121	Macrophage-capping protein OS=Homo sapiens OX=9606 GN=CAPG PE=1 SV=2	6.19	38.5
P01699	Immunoglobulin lambda variable 1-44 OS=Homo sapiens OX=9606 GN=IGLV1-44 PE=1 SV=2	5.17	12.2
P15328	Folate receptor alpha OS=Homo sapiens OX=9606 GN=FOLR1 PE=1 SV=3	7.97	29.8
P49862	Kallikrein-7 OS=Homo sapiens OX=9606 GN=KLK7 PE=1 SV=1	8.47	27.5
P08670	Vimentin OS=Homo sapiens OX=9606 GN=VIM PE=1 SV=4	5.12	53.6
P13489	Ribonuclease inhibitor OS=Homo sapiens OX=9606 GN=RNH1 PE=1 SV=2	4.82	49.9
Q02487	Desmocollin-2 OS=Homo sapiens OX=9606 GN=DSC2 PE=1 SV=1	5.34	99.9
P27482	Calmodulin-like protein 3 OS=Homo sapiens OX=9606 GN=CALML3 PE=1 SV=2	4.42	16.9
Q9BXJ1	Complement C1q tumor necrosis factor-related protein 1 OS=Homo sapiens OX=9606 GN=C1QTNF1 PE=1 SV=1	6.9	31.7
P16152	Carbonyl reductase [NADPH] 1 OS=Homo sapiens OX=9606 GN=CBR1 PE=1 SV=3	8.32	30.4
Q01518	Adenylyl cyclase-associated protein 1 OS=Homo sapiens OX=9606 GN=CAP1 PE=1 SV=5	8.06	51.9
P14550	Aldo-keto reductase family 1 member A1 OS=Homo sapiens OX=9606 GN=AKR1A1 PE=1 SV=3	6.79	36.6
O60603	Toll-like receptor 2 OS=Homo sapiens OX=9606 GN=TLR2 PE=1 SV=1	6.61	89.8

P01042	Kininogen-1 OS=Homo sapiens OX=9606 GN=KNG1 PE=1 SV=2	6.81	71.9
P09958	Furin OS=Homo sapiens OX=9606 GN=FURIN PE=1 SV=2	6.47	86.6
P07384	Calpain-1 catalytic subunit OS=Homo sapiens OX=9606 GN=CAPN1 PE=1 SV=1	5.67	81.8
P14780	Matrix metalloproteinase-9 OS=Homo sapiens OX=9606 GN=MMP9 PE=1 SV=3	6.06	78.4
Q8IWU5	Extracellular sulfatase Sulf-2 OS=Homo sapiens OX=9606 GN=SULF2 PE=1 SV=1	9.17	100.4
F5GXS0	C4a anaphylatoxin OS=Homo sapiens OX=9606 GN=C4B PE=1 SV=1	7.33	187.6
A0A6Q8PFJ0	Prelamin-A/C OS=Homo sapiens OX=9606 GN=LMNA PE=4 SV=1	8.27	80.9
P61158	Actin-related protein 3 OS=Homo sapiens OX=9606 GN=ACTR3 PE=1 SV=3	5.88	47.3
O75083	WD repeat-containing protein 1 OS=Homo sapiens OX=9606 GN=WDR1 PE=1 SV=4	6.65	66.2
A0A2R8Y7R2	Hemoglobin subunit beta OS=Homo sapiens OX=9606 GN=HBB PE=1 SV=1	6.05	12.2
Q99497	Parkinson disease protein 7 OS=Homo sapiens OX=9606 GN=PARK7 PE=1 SV=2	6.79	19.9
A0A075B6K0	Immunoglobulin lambda variable 3-16 OS=Homo sapiens OX=9606 GN=IGLV3-16 PE=3 SV=2	4.65	12.5
P50591	Tumor necrosis factor ligand superfamily member 10 OS=Homo sapiens OX=9606 GN=TNFSF10 PE=1 SV=1	7.42	32.5
Q8NFL0	UDP-GlcNAc:betaGal beta-1,3-N-acetylglucosaminyltransferase 7 OS=Homo sapiens OX=9606 GN=B3GNT7 PE=1 SV=1	9.03	46
Q5T750	Skin-specific protein 32 OS=Homo sapiens OX=9606 GN=XP32 PE=1 SV=1	7.97	26.2
P19652	Alpha-1-acid glycoprotein 2 OS=Homo sapiens OX=9606 GN=ORM2 PE=1 SV=2	5.11	23.6
P22735	Protein-glutamine gamma-glutamyltransferase K OS=Homo sapiens OX=9606 GN=TGM1 PE=1 SV=4	6.04	89.7
A0A5F9ZHM4	L-lactate dehydrogenase OS=Homo sapiens OX=9606 GN=LDHB PE=1 SV=1	6.25	37.4
P17405	Sphingomyelin phosphodiesterase OS=Homo sapiens OX=9606 GN=SMPD1 PE=1 SV=5	7.28	69.9
Q14435	Polypeptide N-acetylgalactosaminyltransferase 3 OS=Homo sapiens OX=9606 GN=GALNT3 PE=1 SV=2	7.99	72.6
P08238	Heat shock protein HSP 90-beta OS=Homo sapiens OX=9606 GN=HSP90AB1 PE=1 SV=4	5.03	83.2
Q9Y5Z4	Heme-binding protein 2 OS=Homo sapiens OX=9606 GN=HEBP2 PE=1 SV=1	4.63	22.9
P21980	Protein-glutamine gamma-glutamyltransferase 2 OS=Homo sapiens OX=9606 GN=TGM2 PE=1 SV=2	5.22	77.3
J3KPJ0	Glutathione hydrolase 6 OS=Homo sapiens OX=9606 GN=GGT6 PE=1 SV=1	6.14	51.1
Q6NUJ1	Proactivator polypeptide-like 1 OS=Homo sapiens OX=9606 GN=PSAPL1 PE=2 SV=2	7.27	56.6
U3KPS2	Myeloblastin OS=Homo sapiens OX=9606 GN=PRTN3 PE=1 SV=1	8.16	23.6
P42357	Histidine ammonia-lyase OS=Homo sapiens OX=9606 GN=HAL PE=1 SV=1	6.95	72.7
P27797	Calreticulin OS=Homo sapiens OX=9606 GN=CALR PE=1 SV=1	4.44	48.1

A2IBA6	Lipase member H OS=Homo sapiens OX=9606 GN=LIPH PE=1 SV=1	8.15	47.2
P18510	Interleukin-1 receptor antagonist protein OS=Homo sapiens OX=9606 GN=IL1RN PE=1 SV=1	6.19	20
Q9HBR0	Putative sodium-coupled neutral amino acid transporter 10 OS=Homo sapiens OX=9606 GN=SLC38A10 PE=1 SV=2	5.73	119.7
P01817	Immunoglobulin heavy variable 2-5 OS=Homo sapiens OX=9606 GN=IGHV2-5 PE=1 SV=2	8.29	13.2
P08311	Cathepsin G OS=Homo sapiens OX=9606 GN=CTSG PE=1 SV=2	11.19	28.8
P46940	Ras GTPase-activating-like protein IQGAP1 OS=Homo sapiens OX=9606 GN=IQGAP1 PE=1 SV=1	6.48	189.1
P41218	Myeloid cell nuclear differentiation antigen OS=Homo sapiens OX=9606 GN=MNDA PE=1 SV=1	9.76	45.8
P01019	Angiotensinogen OS=Homo sapiens OX=9606 GN=AGT PE=1 SV=1	6.32	53.1
Q09666	Neuroblast differentiation-associated protein AHNAK OS=Homo sapiens OX=9606 GN=AHNAK PE=1 SV=2	6.15	628.7
E7EWC6	DNA-directed RNA polymerase II subunit RPB11-b2 (Fragment) OS=Homo sapiens OX=9606 GN=POLR2J3 PE=4 SV=1	8.47	30.4
Q9NS98	Semaphorin-3G OS=Homo sapiens OX=9606 GN=SEMA3G PE=1 SV=1	7.78	86.6
O14745	Na(+)/H(+) exchange regulatory cofactor NHE-RF1 OS=Homo sapiens OX=9606 GN=SLC9A3R1 PE=1 SV=4	5.77	38.8
P49913	Cathelicidin antimicrobial peptide OS=Homo sapiens OX=9606 GN=CAMP PE=1 SV=1	9.41	19.3
A0A0D9SF54	Spectrin alpha chain, non-erythrocytic 1 OS=Homo sapiens OX=9606 GN=SPTAN1 PE=1 SV=1	5.34	282.7
A0A140T953	HLA class I histocompatibility antigen, B alpha chain (Fragment) OS=Homo sapiens OX=9606 GN=HLA-B PE=1 SV=1	6.44	27.7
Q9Y2A9	N-acetyllactosaminide beta-1,3-N-acetylglucosaminyltransferase 3 OS=Homo sapiens OX=9606 GN=B3GNT3 PE=1 SV=2	8.68	42.5
A0A087X1Z3	Proteasome activator complex subunit 2 OS=Homo sapiens OX=9606 GN=PSME2 PE=1 SV=1	6.71	29.1
O95793	Double-stranded RNA-binding protein Staufen homolog 1 OS=Homo sapiens OX=9606 GN=STAU1 PE=1 SV=2	9.44	63.1
O95994	Anterior gradient protein 2 homolog OS=Homo sapiens OX=9606 GN=AGR2 PE=1 SV=1	9	20
Q9UHD0	Interleukin-19 OS=Homo sapiens OX=9606 GN=IL19 PE=1 SV=2	7.69	20.4
P54802	Alpha-N-acetylglucosaminidase OS=Homo sapiens OX=9606 GN=NAGLU PE=1 SV=2	6.65	82.2
A0A1R3UHJ7	Kallikrein M OS=Homo sapiens OX=9606 GN=KLK14 PE=1 SV=1	8.98	27.4
P09467	Fructose-1,6-bisphosphatase 1 OS=Homo sapiens OX=9606 GN=FBP1 PE=1 SV=5	6.99	36.8
A0A0B4J1U3	Immunoglobulin lambda variable 1-36 OS=Homo sapiens OX=9606 GN=IGLV1-36 PE=1 SV=5	4.78	12.5
H7C0V9	ABPP (Fragment) OS=Homo sapiens OX=9606 GN=APP PE=1 SV=1	4.82	55.1
P11413	Glucose-6-phosphate 1-dehydrogenase OS=Homo sapiens OX=9606 GN=G6PD PE=1 SV=4	6.84	59.2
Q9NZP8	Complement C1r subcomponent-like protein OS=Homo sapiens OX=9606 GN=C1RL PE=1 SV=2	7.2	53.5
P02765	Alpha-2-HS-glycoprotein OS=Homo sapiens OX=9606 GN=AHSG PE=1 SV=2	5.72	39.3
P00734	Prothrombin OS=Homo sapiens OX=9606 GN=F2 PE=1 SV=2	5.9	70

F8VWW8	Acid sphingomyelinase-like phosphodiesterase 3b OS=Homo sapiens OX=9606 GN=SMPDL3B PE=1 SV=1	5.34	45.3
A0A087WVQ6	Clathrin heavy chain OS=Homo sapiens OX=9606 GN=CLTC PE=1 SV=1	5.69	191.9
Q16270	Insulin-like growth factor-binding protein 7 OS=Homo sapiens OX=9606 GN=IGFBP7 PE=1 SV=1	7.9	29.1
K7EKI0	Envoplakin OS=Homo sapiens OX=9606 GN=EVPL PE=1 SV=1	7.25	233.7
Q15365	Poly(rC)-binding protein 1 OS=Homo sapiens OX=9606 GN=PCBP1 PE=1 SV=2	7.09	37.5
C9JU00	Fibrinogen gamma chain (Fragment) OS=Homo sapiens OX=9606 GN=FGG PE=1 SV=1	7.2	14
A0A140T9H5	HLA class I histocompatibility antigen, C alpha chain OS=Homo sapiens OX=9606 GN=HLA-C PE=1 SV=1	6	41.3
P61224	Ras-related protein Rap-1b OS=Homo sapiens OX=9606 GN=RAP1B PE=1 SV=1	5.78	20.8
P52566	Rho GDP-dissociation inhibitor 2 OS=Homo sapiens OX=9606 GN=ARHGDIB PE=1 SV=3	5.21	23
P21246	Pleiotrophin OS=Homo sapiens OX=9606 GN=PTN PE=1 SV=1	9.6	18.9
P00747	Plasminogen OS=Homo sapiens OX=9606 GN=PLG PE=1 SV=2	7.24	90.5
O75629	Protein CREG1 OS=Homo sapiens OX=9606 GN=CREG1 PE=1 SV=1	7.59	24.1
A0A0J9YX89	Selenoprotein F (Fragment) OS=Homo sapiens OX=9606 GN=SELENOF PE=1 SV=1	5	10.2
E7EPV7	Alpha-synuclein OS=Homo sapiens OX=9606 GN=SNCA PE=1 SV=1	9.23	11.8
P0DP25	Calmodulin-3 OS=Homo sapiens OX=9606 GN=CALM3 PE=1 SV=1	4.22	16.8
Q5JR08	Rho-related GTP-binding protein RhoC (Fragment) OS=Homo sapiens OX=9606 GN=RHOC PE=1 SV=8	6.58	21.5
P04080	Cystatin-B OS=Homo sapiens OX=9606 GN=CSTB PE=1 SV=2	7.56	11.1
Q9BTY2	Plasma alpha-L-fucosidase OS=Homo sapiens OX=9606 GN=FUCA2 PE=1 SV=2	6.25	54
O75355	Ectonucleoside triphosphate diphosphohydrolase 3 OS=Homo sapiens OX=9606 GN=ENTPD3 PE=1 SV=2	6.42	59.1
Q9HCY8	Protein S100-A14 OS=Homo sapiens OX=9606 GN=S100A14 PE=1 SV=1	5.24	11.7
B1AK87	F-actin-capping protein subunit beta OS=Homo sapiens OX=9606 GN=CAPZB PE=1 SV=2	5.55	27.4
P51993	4-galactosyl-N-acetylglucosaminide 3-alpha-L-fucosyltransferase FUT6 OS=Homo sapiens OX=9606 GN=FUT6 PE=1 SV=1	8.68	41.8
D6RC73	C-C motif chemokine 28 OS=Homo sapiens OX=9606 GN=CCL28 PE=1 SV=1	10.49	9.3
A0A0C4DH36	Probable non-functional immunoglobulin heavy variable 3-38 OS=Homo sapiens OX=9606 GN=IGHV3-38 PE=1 SV=1	9.25	12.8
F8WE86	Transcobalamin-2 OS=Homo sapiens OX=9606 GN=TCN2 PE=1 SV=2	7.25	44.8
P33908	Mannosyl-oligosaccharide 1,2-alpha-mannosidase IA OS=Homo sapiens OX=9606 GN=MAN1A1 PE=1 SV=3	6.47	72.9
P55072	Transitional endoplasmic reticulum ATPase OS=Homo sapiens OX=9606 GN=VCP PE=1 SV=4	5.26	89.3
P10253	Lysosomal alpha-glucosidase OS=Homo sapiens OX=9606 GN=GAA PE=1 SV=4	6	105.3
Q13183	Solute carrier family 13 member 2 OS=Homo sapiens OX=9606 GN=SLC13A2 PE=1 SV=1	7.01	64.4

Q16769	Glutaminyl-peptide cyclotransferase OS=Homo sapiens OX=9606 GN=QPCT PE=1 SV=1	6.61	40.9
O95969	Secretoglobin family 1D member 2 OS=Homo sapiens OX=9606 GN=SCGB1D2 PE=2 SV=1	8.25	9.9
P21964	Catechol O-methyltransferase OS=Homo sapiens OX=9606 GN=COMT PE=1 SV=2	5.47	30
P14174	Macrophage migration inhibitory factor OS=Homo sapiens OX=9606 GN=MIF PE=1 SV=4	7.88	12.5
P37837	Transaldolase OS=Homo sapiens OX=9606 GN=TALDO1 PE=1 SV=2	6.81	37.5
P26572	Alpha-1,3-mannosyl-glycoprotein 2-beta-N-acetylglucosaminyltransferase OS=Homo sapiens OX=9606 GN=MGAT1 PE=1 SV=2	9.16	50.8
Q14050	Collagen alpha-3(IX) chain OS=Homo sapiens OX=9606 GN=COL9A3 PE=1 SV=2	7.68	63.6
P0DTE1	Probable non-functional immunoglobulin heavy variable 3-38-3 OS=Homo sapiens OX=9606 GN=IGHV3-38-3 PE=1 SV=1	9.25	12.7
P25815	Protein S100-P OS=Homo sapiens OX=9606 GN=S100P PE=1 SV=2	4.88	10.4
O15144	Actin-related protein 2/3 complex subunit 2 OS=Homo sapiens OX=9606 GN=ARPC2 PE=1 SV=1	7.36	34.3
O60784	Target of Myb protein 1 OS=Homo sapiens OX=9606 GN=TOM1 PE=1 SV=2	4.7	53.8
P62834	Ras-related protein Rap-1A OS=Homo sapiens OX=9606 GN=RAP1A PE=1 SV=1	6.67	21
D6RHX1	Mucin-7 (Fragment) OS=Homo sapiens OX=9606 GN=MUC7 PE=1 SV=1	10.07	15.5
P02649	Apolipoprotein E OS=Homo sapiens OX=9606 GN=APOE PE=1 SV=1	5.73	36.1
A0A075B6J1	Immunoglobulin lambda variable 5-37 OS=Homo sapiens OX=9606 GN=IGLV5-37 PE=3 SV=1	4.94	13.3
P30085	UMP-CMP kinase OS=Homo sapiens OX=9606 GN=CMPK1 PE=1 SV=3	5.57	22.2
P09960	Leukotriene A-4 hydrolase OS=Homo sapiens OX=9606 GN=LTA4H PE=1 SV=2	6.18	69.2
P19971	Thymidine phosphorylase OS=Homo sapiens OX=9606 GN=TYMP PE=1 SV=2	5.53	49.9
P21741	Midkine OS=Homo sapiens OX=9606 GN=MDK PE=1 SV=1	9.79	15.6
Q9ULZ3	Apoptosis-associated speck-like protein containing a CARD OS=Homo sapiens OX=9606 GN=PYCARD PE=1 SV=2	6.34	21.6
Q92820	Gamma-glutamyl hydrolase OS=Homo sapiens OX=9606 GN=GGH PE=1 SV=2	7.11	35.9
P59998	Actin-related protein 2/3 complex subunit 4 OS=Homo sapiens OX=9606 GN=ARPC4 PE=1 SV=3	8.43	19.7
O95084	Serine protease 23 OS=Homo sapiens OX=9606 GN=PRSS23 PE=1 SV=1	9.42	43
Q96MK3	Pseudokinase FAM20A OS=Homo sapiens OX=9606 GN=FAM20A PE=1 SV=4	7.99	61.4
Q07654	Trefoil factor 3 OS=Homo sapiens OX=9606 GN=TFF3 PE=1 SV=2	6.92	10.2
A0A075B6S9	Probable non-functional immunoglobulinn kappa variable 1-37 OS=Homo sapiens OX=9606 GN=IGKV1-37 PE=1 SV=7	9.25	12.7
P01040	Cystatin-A OS=Homo sapiens OX=9606 GN=CSTA PE=1 SV=1	5.5	11
P05091	Aldehyde dehydrogenase, mitochondrial OS=Homo sapiens OX=9606 GN=ALDH2 PE=1 SV=2	7.05	56.3
P00491	Purine nucleoside phosphorylase OS=Homo sapiens OX=9606 GN=PNP PE=1 SV=2	6.95	32.1

P04196	Histidine-rich glycoprotein OS=Homo sapiens OX=9606 GN=HRG PE=1 SV=1	7.5	59.5
P08246	Neutrophil elastase OS=Homo sapiens OX=9606 GN=ELANE PE=1 SV=1	9.35	28.5
Q9UI42	Carboxypeptidase A4 OS=Homo sapiens OX=9606 GN=CPA4 PE=1 SV=2	6.7	47.3
P15531	Nucleoside diphosphate kinase A OS=Homo sapiens OX=9606 GN=NME1 PE=1 SV=1	6.19	17.1
Q96FQ6	Protein S100-A16 OS=Homo sapiens OX=9606 GN=S100A16 PE=1 SV=1	6.79	11.8
O60664	Perilipin-3 OS=Homo sapiens OX=9606 GN=PLIN3 PE=1 SV=3	5.44	47
Q8NI22	Multiple coagulation factor deficiency protein 2 OS=Homo sapiens OX=9606 GN=MCFD2 PE=1 SV=1	4.63	16.4
P35475	Alpha-L-iduronidase OS=Homo sapiens OX=9606 GN=IDUA PE=1 SV=2	9.14	72.6
P11279	Lysosome-associated membrane glycoprotein 1 OS=Homo sapiens OX=9606 GN=LAMP1 PE=1 SV=3	8.75	44.9
F6RFD5	Actin-depolymerizing factor OS=Homo sapiens OX=9606 GN=DSTN PE=1 SV=1	8.59	15.4
E9PRJ8	Tetraspanin (Fragment) OS=Homo sapiens OX=9606 GN=CD81 PE=1 SV=1	6.15	22.5
Q9UN76	Sodium- and chloride-dependent neutral and basic amino acid transporter B(0+) OS=Homo sapiens OX=9606 GN=SLC6A14 PE=2 SV=1	8.19	72.1
P09237	Matrilysin OS=Homo sapiens OX=9606 GN=MMP7 PE=1 SV=1	7.91	29.7
F8W6I7	Helix-destabilizing protein OS=Homo sapiens OX=9606 GN=HNRNPA1 PE=1 SV=2	9.13	33.1
A0A3B3IUC4	Alpha-galactosidase OS=Homo sapiens OX=9606 GN=GLA PE=1 SV=1	5.86	53.2
A8K2U0	Alpha-2-macroglobulin-like protein 1 OS=Homo sapiens OX=9606 GN=A2ML1 PE=1 SV=3	5.73	161
Q8NBS9	Thioredoxin domain-containing protein 5 OS=Homo sapiens OX=9606 GN=TXNDC5 PE=1 SV=2	5.97	47.6
P13473	Lysosome-associated membrane glycoprotein 2 OS=Homo sapiens OX=9606 GN=LAMP2 PE=1 SV=2	5.63	44.9
Q9HC84	Mucin-5B OS=Homo sapiens OX=9606 GN=MUC5B PE=1 SV=3	6.64	596
P17655	Calpain-2 catalytic subunit OS=Homo sapiens OX=9606 GN=CAPN2 PE=1 SV=6	4.98	79.9
Q5SGD2	Protein phosphatase 1L OS=Homo sapiens OX=9606 GN=PPM1L PE=1 SV=1	5.87	41
Q12841	Follistatin-related protein 1 OS=Homo sapiens OX=9606 GN=FSTL1 PE=1 SV=1	5.52	35
A0A0U1RQV3	EGF-containing fibulin-like extracellular matrix protein 1 (Fragment) OS=Homo sapiens OX=9606 GN=EFEMP1 PE=1 SV=1	6.62	31.7
P20933	N(4)-(beta-N-acetylglucosaminyl)-L-asparaginase OS=Homo sapiens OX=9606 GN=AGA PE=1 SV=2	6.28	37.2
A0A075B6H9	Immunoglobulin lambda variable 4-69 OS=Homo sapiens OX=9606 GN=IGLV4-69 PE=1 SV=1	6.51	12.8
P31146	Coronin-1A OS=Homo sapiens OX=9606 GN=CORO1A PE=1 SV=4	6.68	51
O15143	Actin-related protein 2/3 complex subunit 1B OS=Homo sapiens OX=9606 GN=ARPC1B PE=1 SV=3	8.35	40.9
Q9UHL4	Dipeptidyl peptidase 2 OS=Homo sapiens OX=9606 GN=DPP7 PE=1 SV=3	6.32	54.3
P43490	Nicotinamide phosphoribosyltransferase OS=Homo sapiens OX=9606 GN=NAMPT PE=1 SV=1	7.15	55.5
O14657	Torsin-1B OS=Homo sapiens OX=9606 GN=TOR1B PE=1 SV=2	8.54	38
Q14574	Desmocollin-3 OS=Homo sapiens OX=9606 GN=DSC3 PE=1 SV=3	6.1	99.9

Q00115	Deoxyribonuclease-2-alpha OS=Homo sapiens OX=9606 GN=DNASE2 PE=1 SV=2	8.05	39.6
P61204	ADP-ribosylation factor 3 OS=Homo sapiens OX=9606 GN=ARF3 PE=1 SV=2	7.43	20.6
P47895	Aldehyde dehydrogenase family 1 member A3 OS=Homo sapiens OX=9606 GN=ALDH1A3 PE=1 SV=2	7.25	56.1
A0A0G2JLB3	Glucosylceramidase OS=Homo sapiens OX=9606 GN=GBA PE=1 SV=1	7.42	59.6
P19823	Inter-alpha-trypsin inhibitor heavy chain H2 OS=Homo sapiens OX=9606 GN=ITIH2 PE=1 SV=2	6.86	106. 4
P62879	Guanine nucleotide-binding protein G(I)/G(S)/G(T) subunit beta-2 OS=Homo sapiens OX=9606 GN=GNB2 PE=1 SV=3	6	37.3
I3L0K2	Thioredoxin domain-containing protein 17 OS=Homo sapiens OX=9606 GN=TXNDC17 PE=1 SV=1	5.76	8.4
Q06323	Proteasome activator complex subunit 1 OS=Homo sapiens OX=9606 GN=PSME1 PE=1 SV=1	6.02	28.7
A0A087WY61	Nuclear mitotic apparatus protein 1 OS=Homo sapiens OX=9606 GN=NUMA1 PE=1 SV=1	5.92	236
O14950	Myosin regulatory light chain 12B OS=Homo sapiens OX=9606 GN=MYL12B PE=1 SV=2	4.84	19.8
Q92928	Putative Ras-related protein Rab-1C OS=Homo sapiens OX=9606 GN=RAB1C PE=5 SV=2	5.43	22
P52790	Hexokinase-3 OS=Homo sapiens OX=9606 GN=HK3 PE=1 SV=2	5.4	99
Q15084	Protein disulfide-isomerase A6 OS=Homo sapiens OX=9606 GN=PDIA6 PE=1 SV=1	5.08	48.1
Q96TA1	Protein Niban 2 OS=Homo sapiens OX=9606 GN=NIBAN2 PE=1 SV=3	6.19	84.1
Q16665	Hypoxia-inducible factor 1-alpha OS=Homo sapiens OX=9606 GN=HIF1A PE=1 SV=1	5.33	92.6
P08865	40S ribosomal protein SA OS=Homo sapiens OX=9606 GN=RPSA PE=1 SV=4	4.87	32.8
Q9HB40	Retinoid-inducible serine carboxypeptidase OS=Homo sapiens OX=9606 GN=SCPEP1 PE=1 SV=1	5.81	50.8
K7EQM7	Urea transporter 1 OS=Homo sapiens OX=9606 GN=SLC14A1 PE=1 SV=2	5.63	23.3
Q96KP4	Cytosolic non-specific dipeptidase OS=Homo sapiens OX=9606 GN=CNDP2 PE=1 SV=2	5.97	52.8
Q13332	Receptor-type tyrosine-protein phosphatase S OS=Homo sapiens OX=9606 GN=PTPRS PE=1 SV=3	6.46	216. 9
Q53RT3	Retroviral-like aspartic protease 1 OS=Homo sapiens OX=9606 GN=ASPRV1 PE=1 SV=1	5.44	37
P16402	Histone H1.3 OS=Homo sapiens OX=9606 GN=H1-3 PE=1 SV=2	11.0 2	22.3
P58107	Epiplakin OS=Homo sapiens OX=9606 GN=EPPK1 PE=1 SV=3	5.62	555. 3
J3KPA1	Cysteine-rich secretory protein 3 OS=Homo sapiens OX=9606 GN=CRISP3 PE=1 SV=1	7.61	31
A0A087WY35	Integrin beta-like protein 1 OS=Homo sapiens OX=9606 GN=ITGBL1 PE=1 SV=1	5.4	48.5
Q8N695	Sodium-coupled monocarboxylate transporter 1 OS=Homo sapiens OX=9606 GN=SLC5A8 PE=1 SV=2	7.75	66.5
P19827	Inter-alpha-trypsin inhibitor heavy chain H1 OS=Homo sapiens OX=9606 GN=ITIH1 PE=1 SV=3	6.79	101. 3
Q9Y337	Kallikrein-5 OS=Homo sapiens OX=9606 GN=KLK5 PE=1 SV=3	8.27	32

P60842	Eukaryotic initiation factor 4A-I OS=Homo sapiens OX=9606 GN=EIF4A1 PE=1 SV=1	5.48	46.1
O14672	Disintegrin and metalloproteinase domain-containing protein 10 OS=Homo sapiens OX=9606 GN=ADAM10 PE=1 SV=1	7.77	84.1
P10619	Lysosomal protective protein OS=Homo sapiens OX=9606 GN=CTSA PE=1 SV=2	6.61	54.4
J3KN67	Tropomyosin alpha-3 chain OS=Homo sapiens OX=9606 GN=TPM3 PE=1 SV=1	4.77	33.2
Q8N4A0	Polypeptide N-acetylgalactosaminyltransferase 4 OS=Homo sapiens OX=9606 GN=GALNT4 PE=1 SV=2	7.61	66.6
Q86VR7	V-set and immunoglobulin domain-containing protein 10-like OS=Homo sapiens OX=9606 GN=VSIG10L PE=2 SV=2	7.78	91.6
B7ZKJ8	ITIH4 protein OS=Homo sapiens OX=9606 GN=ITIH4 PE=1 SV=1	6.89	103.8
F8VZX2	Poly(rC)-binding protein 2 OS=Homo sapiens OX=9606 GN=PCBP2 PE=1 SV=1	8.24	33.8
A0A087WWU8	Tropomyosin alpha-3 chain OS=Homo sapiens OX=9606 GN=TPM3 PE=1 SV=1	4.78	26.4
P22626	Heterogeneous nuclear ribonucleoproteins A2/B1 OS=Homo sapiens OX=9606 GN=HNRNPA2B1 PE=1 SV=2	8.95	37.4
G8JLG2	Corneodesmosin OS=Homo sapiens OX=9606 GN=CDSN PE=1 SV=1	8.44	51.6
A0A2R8Y5V9	Tropomyosin alpha-4 chain OS=Homo sapiens OX=9606 GN=TPM4 PE=1 SV=1	4.7	28.6
H0YHX9	Nascent polypeptide-associated complex subunit alpha OS=Homo sapiens OX=9606 GN=NACA PE=1 SV=2	4.55	25.3
H0YJT9	N-myc downstream-regulated gene 2 protein (Fragment) OS=Homo sapiens OX=9606 GN=NDRG2 PE=1 SV=1	7.08	31.3
P61812	Transforming growth factor beta-2 proprotein OS=Homo sapiens OX=9606 GN=TGFB2 PE=1 SV=1	8.53	47.7
Q13228	Methanethiol oxidase OS=Homo sapiens OX=9606 GN=SELENBP1 PE=1 SV=2	6.37	52.4
E7EQ64	Trypsin-1 OS=Homo sapiens OX=9606 GN=PRSS1 PE=1 SV=1	7.25	28.1
P49720	Proteasome subunit beta type-3 OS=Homo sapiens OX=9606 GN=PSMB3 PE=1 SV=2	6.55	22.9
F8VR50	Actin-related protein 2/3 complex subunit 3 (Fragment) OS=Homo sapiens OX=9606 GN=ARPC3 PE=1 SV=1	7.93	9.7
P05120	Plasminogen activator inhibitor 2 OS=Homo sapiens OX=9606 GN=SERPINB2 PE=1 SV=2	5.63	46.6
Q5VVC8	60S ribosomal protein L11 OS=Homo sapiens OX=9606 GN=RPL11 PE=1 SV=2	9.8	19
Q14974	Importin subunit beta-1 OS=Homo sapiens OX=9606 GN=KPNB1 PE=1 SV=2	4.78	97.1
P32320	Cytidine deaminase OS=Homo sapiens OX=9606 GN=CDA PE=1 SV=2	6.92	16.2
P10586	Receptor-type tyrosine-protein phosphatase F OS=Homo sapiens OX=9606 GN=PTPRF PE=1 SV=2	6.3	212.7
O75841	Uroplakin-1b OS=Homo sapiens OX=9606 GN=UPK1B PE=1 SV=5	5.35	29.6
P61970	Nuclear transport factor 2 OS=Homo sapiens OX=9606 GN=NUTF2 PE=1 SV=1	5.38	14.5
D6RH31	Nephronectin (Fragment) OS=Homo sapiens OX=9606 GN=NPNT PE=1 SV=1	8.4	66.9

P02751	Fibronectin OS=Homo sapiens OX=9606 GN=FN1 PE=1 SV=5	5.5	272.2
E9PR14	Serum amyloid A protein OS=Homo sapiens OX=9606 GN=SAA2 PE=3 SV=1	6.02	8.9
P08493	Matrix Gla protein OS=Homo sapiens OX=9606 GN=MGP PE=1 SV=2	9.67	12.3
P26641	Elongation factor 1-gamma OS=Homo sapiens OX=9606 GN=EEF1G PE=1 SV=3	6.67	50.1
B4DPQ0	Complement subcomponent C1r OS=Homo sapiens OX=9606 GN=C1R PE=1 SV=1	6.37	81.8
Q5XXA6	Anoctamin-1 OS=Homo sapiens OX=9606 GN=ANO1 PE=1 SV=1	8.54	114
P63241	Eukaryotic translation initiation factor 5A-1 OS=Homo sapiens OX=9606 GN=EIF5A PE=1 SV=2	5.24	16.8
Q9BS26	Endoplasmic reticulum resident protein 44 OS=Homo sapiens OX=9606 GN=ERP44 PE=1 SV=1	5.26	46.9
Q9UBS4	DnaJ homolog subfamily B member 11 OS=Homo sapiens OX=9606 GN=DNAJB11 PE=1 SV=1	6.18	40.5
P17858	ATP-dependent 6-phosphofructokinase, liver type OS=Homo sapiens OX=9606 GN=PFKL PE=1 SV=6	7.5	85
O75594	Peptidoglycan recognition protein 1 OS=Homo sapiens OX=9606 GN=PGLYRP1 PE=1 SV=1	8.59	21.7
Q99536	Synaptic vesicle membrane protein VAT-1 homolog OS=Homo sapiens OX=9606 GN=VAT1 PE=1 SV=2	6.29	41.9
A0A2R8YDT1	Glutamine synthetase OS=Homo sapiens OX=9606 GN=GLUL PE=1 SV=1	8.37	57.1
E9PS12	Alpha-crystallin B chain (Fragment) OS=Homo sapiens OX=9606 GN=CRYAB PE=1 SV=1	6.95	9.2
P09758	Tumor-associated calcium signal transducer 2 OS=Homo sapiens OX=9606 GN=TACSTD2 PE=1 SV=3	8.87	35.7
P01133	Pro-epidermal growth factor OS=Homo sapiens OX=9606 GN=EGF PE=1 SV=2	5.85	133.9
A0A075B6K6	Immunoglobulin lambda variable 4-3 OS=Homo sapiens OX=9606 GN=IGLV4-3 PE=3 SV=1	5.71	13.3
P05387	60S acidic ribosomal protein P2 OS=Homo sapiens OX=9606 GN=RPLP2 PE=1 SV=1	4.54	11.7
P05386	60S acidic ribosomal protein P1 OS=Homo sapiens OX=9606 GN=RPLP1 PE=1 SV=1	4.32	11.5
Q15847	Adipogenesis regulatory factor OS=Homo sapiens OX=9606 GN=ADIRF PE=1 SV=1	5.31	7.9
P62491	Ras-related protein Rab-11A OS=Homo sapiens OX=9606 GN=RAB11A PE=1 SV=3	6.57	24.4
Q9P1F3	Costars family protein ABRACL OS=Homo sapiens OX=9606 GN=ABRACL PE=1 SV=1	6.29	9.1
O43653	Prostate stem cell antigen OS=Homo sapiens OX=9606 GN=PSCA PE=1 SV=2	4.94	12
P40926	Malate dehydrogenase, mitochondrial OS=Homo sapiens OX=9606 GN=MDH2 PE=1 SV=3	8.68	35.5
Q6P4A8	Phospholipase B-like 1 OS=Homo sapiens OX=9606 GN=PLBD1 PE=1 SV=2	9.06	63.2
P55259	Pancreatic secretory granule membrane major glycoprotein GP2 OS=Homo sapiens OX=9606 GN=GP2 PE=1 SV=3	5.24	59.4
Q9UM07	Protein-arginine deiminase type-4 OS=Homo sapiens OX=9606 GN=PADI4 PE=1 SV=2	6.58	74

Q11206	CMP-N-acetylneuraminate-beta-galactosamide-alpha-2,3-sialyltransferase 4 OS=Homo sapiens OX=9606 GN=ST3GAL4 PE=1 SV=1	9.41	38
O00754	Lysosomal alpha-mannosidase OS=Homo sapiens OX=9606 GN=MAN2B1 PE=1 SV=3	7.28	113.7
P15153	Ras-related C3 botulinum toxin substrate 2 OS=Homo sapiens OX=9606 GN=RAC2 PE=1 SV=1	7.61	21.4
P52565	Rho GDP-dissociation inhibitor 1 OS=Homo sapiens OX=9606 GN=ARHGDIA PE=1 SV=3	5.11	23.2
P08133	Annexin A6 OS=Homo sapiens OX=9606 GN=ANXA6 PE=1 SV=3	5.6	75.8
V9GYS1	Apolipoprotein A-II OS=Homo sapiens OX=9606 GN=APOA2 PE=1 SV=1	5.01	9.2
P11047	Laminin subunit gamma-1 OS=Homo sapiens OX=9606 GN=LAMC1 PE=1 SV=3	5.12	177.5
Q16853	Membrane primary amine oxidase OS=Homo sapiens OX=9606 GN=AOC3 PE=1 SV=3	6.52	84.6
H0Y586	Proteasome subunit alpha type-7 (Fragment) OS=Homo sapiens OX=9606 GN=PSMA7 PE=1 SV=1	8.87	21.2
P06727	Apolipoprotein A-IV OS=Homo sapiens OX=9606 GN=APOA4 PE=1 SV=4	5.38	45.3
P26583	High mobility group protein B2 OS=Homo sapiens OX=9606 GN=HMGB2 PE=1 SV=2	7.81	24
G3V3U4	Proteasome subunit alpha type OS=Homo sapiens OX=9606 GN=PSMA6 PE=1 SV=1	9.1	11.6
Q687X5	Metalloreductase STEAP4 OS=Homo sapiens OX=9606 GN=STEAP4 PE=1 SV=1	9.29	51.9
P04899	Guanine nucleotide-binding protein G(i) subunit alpha-2 OS=Homo sapiens OX=9606 GN=GNAI2 PE=1 SV=3	5.54	40.4
P55957	BH3-interacting domain death agonist OS=Homo sapiens OX=9606 GN=BID PE=1 SV=1	5.44	22
P02760	Protein AMBP OS=Homo sapiens OX=9606 GN=AMBP PE=1 SV=1	6.25	39
Q9H4A4	Aminopeptidase B OS=Homo sapiens OX=9606 GN=RNPEP PE=1 SV=2	5.74	72.5
Q13444	Disintegrin and metalloproteinase domain-containing protein 15 OS=Homo sapiens OX=9606 GN=ADAM15 PE=1 SV=4	6.73	92.9
P01880	Immunoglobulin heavy constant delta OS=Homo sapiens OX=9606 GN=IGHD PE=1 SV=3	8.12	42.3
Q5T123	SH3 domain-binding glutamic acid-rich-like protein 3 OS=Homo sapiens OX=9606 GN=SH3BGRL3 PE=1 SV=1	9.36	9.4
A0A590UJJ0	Guanine nucleotide-binding protein G(s) subunit alpha isoforms short (Fragment) OS=Homo sapiens OX=9606 GN=GNAS PE=1 SV=1	4.94	23.9
H0YF32	40S ribosomal protein S3 (Fragment) OS=Homo sapiens OX=9606 GN=RPS3 PE=1 SV=1	8	13.5
P00918	Carbonic anhydrase 2 OS=Homo sapiens OX=9606 GN=CA2 PE=1 SV=2	7.4	29.2
P62241	40S ribosomal protein S8 OS=Homo sapiens OX=9606 GN=RPS8 PE=1 SV=2	10.32	24.2
P28838	Cytosol aminopeptidase OS=Homo sapiens OX=9606 GN=LAP3 PE=1 SV=3	7.93	56.1
P49327	Fatty acid synthase OS=Homo sapiens OX=9606 GN=FASN PE=1 SV=3	6.44	273.3
B1AKG0	Complement factor H-related protein 1 OS=Homo sapiens OX=9606 GN=CFHR1 PE=1 SV=1	7.81	30.8
H0Y8G5	Heterogeneous nuclear ribonucleoprotein D0 (Fragment) OS=Homo sapiens OX=9606 GN=HNRNPD PE=1 SV=8	9.16	29.6

P17213	Bactericidal permeability-increasing protein OS=Homo sapiens OX=9606 GN=BPI PE=1 SV=4	9.38	53.9
E9PLK3	Aminopeptidase OS=Homo sapiens OX=9606 GN=NPEPPS PE=1 SV=1	5.6	102.9
O15041	Semaphorin-3E OS=Homo sapiens OX=9606 GN=SEMA3E PE=1 SV=1	7.47	89.2
Q86UX7	Fermitin family homolog 3 OS=Homo sapiens OX=9606 GN=FERMT3 PE=1 SV=1	6.98	75.9
P02778	C-X-C motif chemokine 10 OS=Homo sapiens OX=9606 GN=CXCL10 PE=1 SV=2	9.86	10.9
P18206	Vinculin OS=Homo sapiens OX=9606 GN=VCL PE=1 SV=4	5.66	123.7
A0A0C4DH39	Immunoglobulin heavy variable 1-58 OS=Homo sapiens OX=9606 GN=IGHV1-58 PE=3 SV=1	9.25	13
E7EMG9	Lymphocyte-specific protein 1 (Fragment) OS=Homo sapiens OX=9606 GN=LSP1 PE=1 SV=1	4.59	30.6
P02656	Apolipoprotein C-III OS=Homo sapiens OX=9606 GN=APOC3 PE=1 SV=1	5.41	10.8
Q86X91	Inactive tyrosine-protein kinase 7 OS=Homo sapiens OX=9606 GN=PTK7 PE=1 SV=1	6.65	50.4
F5H157	Ras-related protein Rab-35 (Fragment) OS=Homo sapiens OX=9606 GN=RAB35 PE=1 SV=1	8.31	21.2
D6R9Z1	Receptor of-activated protein C kinase 1 (Fragment) OS=Homo sapiens OX=9606 GN=RACK1 PE=1 SV=8	8.84	26.3
Q15828	Cystatin-M OS=Homo sapiens OX=9606 GN=CST6 PE=1 SV=1	8.09	16.5
P14138	Endothelin-3 OS=Homo sapiens OX=9606 GN=EDN3 PE=1 SV=1	6.7	25.4
A0A087WUC4	Alcohol dehydrogenase 1C (Fragment) OS=Homo sapiens OX=9606 GN=ADH1C PE=1 SV=1	8.06	17.5
A0A0J9YWB6	RNA polymerase II subunit A C-terminal domain phosphatase (Fragment) OS=Homo sapiens OX=9606 GN=CTDP1 PE=1 SV=1	5.72	87.1
P55795	Heterogeneous nuclear ribonucleoprotein H2 OS=Homo sapiens OX=9606 GN=HNRNPH2 PE=1 SV=1	6.3	49.2
Q13296	Mammaglobin-A OS=Homo sapiens OX=9606 GN=SCGB2A2 PE=1 SV=1	4.3	10.5
P17050	Alpha-N-acetylgalactosaminidase OS=Homo sapiens OX=9606 GN=NAGA PE=1 SV=2	5.19	46.5
P43026	Growth/differentiation factor 5 OS=Homo sapiens OX=9606 GN=GDF5 PE=1 SV=3	9.79	55.4
Q6UXB3	Ly6/PLAUR domain-containing protein 2 OS=Homo sapiens OX=9606 GN=LYPD2 PE=1 SV=1	5.96	13.1
Q2I0M4	Leucine-rich repeat-containing protein 26 OS=Homo sapiens OX=9606 GN=LRRC26 PE=1 SV=2	9.17	34.8
Q5T7C4	High mobility group protein B1 OS=Homo sapiens OX=9606 GN=HMGB1 PE=1 SV=1	9.7	18.3
Q5W0H4	Translationally-controlled tumor protein OS=Homo sapiens OX=9606 GN=TPT1 PE=1 SV=1	5.49	21.5
Q9UBQ7	Glyoxylate reductase/hydroxypyruvate reductase OS=Homo sapiens OX=9606 GN=GRHPR PE=1 SV=1	7.39	35.6
O76027	Annexin A9 OS=Homo sapiens OX=9606 GN=ANXA9 PE=1 SV=3	5.77	38.3
P29692	Elongation factor 1-delta OS=Homo sapiens OX=9606 GN=EEF1D PE=1 SV=5	5.01	31.1
A0A3B3ITK7	Phosphoglucomutase-1 OS=Homo sapiens OX=9606 GN=PGM1 PE=1 SV=1	6.76	63.9

Q92882	Osteoclast-stimulating factor 1 OS=Homo sapiens OX=9606 GN=OSTF1 PE=1 SV=2	5.68	23.8
X6RFL8	Ras-related protein Rab-14 (Fragment) OS=Homo sapiens OX=9606 GN=RAB14 PE=1 SV=1	6.33	20.4
Q9H444	Charged multivesicular body protein 4b OS=Homo sapiens OX=9606 GN=CHMP4B PE=1 SV=1	4.82	24.9
P58546	Myotrophin OS=Homo sapiens OX=9606 GN=MTPN PE=1 SV=2	5.52	12.9
P50995	Annexin A11 OS=Homo sapiens OX=9606 GN=ANXA11 PE=1 SV=1	7.65	54.4
B4DUR8	T-complex protein 1 subunit gamma OS=Homo sapiens OX=9606 GN=CCT3 PE=1 SV=1	5.64	55.6
O95274	Ly6/PLAUR domain-containing protein 3 OS=Homo sapiens OX=9606 GN=LYPD3 PE=1 SV=2	7.75	35.9
A0A1X7SBU6	Adhesion G-protein-coupled receptor V1 (Fragment) OS=Homo sapiens OX=9606 GN=ADGRV1 PE=1 SV=1	4.78	288.2
P43034	Platelet-activating factor acetylhydrolase IB subunit beta OS=Homo sapiens OX=9606 GN=PAFAH1B1 PE=1 SV=2	7.37	46.6
A0A0U1RQV5	Uncharacterized protein (Fragment) OS=Homo sapiens OX=9606 PE=1 SV=1	4.88	11.8
H7C125	Ras-related protein Rab-2A (Fragment) OS=Homo sapiens OX=9606 GN=RAB2A PE=1 SV=1	5.08	12.1
P06576	ATP synthase subunit beta, mitochondrial OS=Homo sapiens OX=9606 GN=ATP5F1B PE=1 SV=3	5.4	56.5
E9PCY7	Heterogeneous nuclear ribonucleoprotein H OS=Homo sapiens OX=9606 GN=HNRNPH1 PE=1 SV=1	6.34	47.1
Q13630	GDP-L-fucose synthase OS=Homo sapiens OX=9606 GN=GFUS PE=1 SV=1	6.6	35.9
P08240	Signal recognition particle receptor subunit alpha OS=Homo sapiens OX=9606 GN=SRPRA PE=1 SV=2	8.95	69.8
E9PHK0	Tetranectin OS=Homo sapiens OX=9606 GN=CLEC3B PE=1 SV=1	5.05	17.8
Q9NQ79	Cartilage acidic protein 1 OS=Homo sapiens OX=9606 GN=CRTAC1 PE=1 SV=2	5.12	71.4
P05156	Complement factor I OS=Homo sapiens OX=9606 GN=CFI PE=1 SV=2	7.5	65.7
P62701	40S ribosomal protein S4, X isoform OS=Homo sapiens OX=9606 GN=RPS4X PE=1 SV=2	10.15	29.6
Q9BYD5	Cornifelin OS=Homo sapiens OX=9606 GN=CNFN PE=1 SV=2	6.1	12.4
P02747	Complement C1q subcomponent subunit C OS=Homo sapiens OX=9606 GN=C1QC PE=1 SV=3	8.41	25.8
P49189	4-trimethylaminobutyraldehyde dehydrogenase OS=Homo sapiens OX=9606 GN=ALDH9A1 PE=1 SV=3	5.87	53.8
O95833	Chloride intracellular channel protein 3 OS=Homo sapiens OX=9606 GN=CLIC3 PE=1 SV=2	6.43	26.6
P05546	Heparin cofactor 2 OS=Homo sapiens OX=9606 GN=SERPIND1 PE=1 SV=3	6.9	57
Q96QA5	Gasdermin-A OS=Homo sapiens OX=9606 GN=GSDMA PE=1 SV=4	5.29	49.3
P08754	Guanine nucleotide-binding protein G(i) subunit alpha OS=Homo sapiens OX=9606 GN=GNAI3 PE=1 SV=3	5.69	40.5
F8W6H5	Ectonucleotide pyrophosphatase/phosphodiesterase family member 3 OS=Homo sapiens OX=9606 GN=ENPP3 PE=1 SV=1	7.03	76
P98095	Fibulin-2 OS=Homo sapiens OX=9606 GN=FBLN2 PE=1 SV=2	4.82	126.5

P52907	F-actin-capping protein subunit alpha-1 OS=Homo sapiens OX=9606 GN=CAPZA1 PE=1 SV=3	5.69	32.9
P21333	Filamin-A OS=Homo sapiens OX=9606 GN=FLNA PE=1 SV=4	6.06	280.6
A0A286YFM8	D-3-phosphoglycerate dehydrogenase (Fragment) OS=Homo sapiens OX=9606 GN=PHGDH PE=1 SV=1	7.58	25.6
P04114	Apolipoprotein B-100 OS=Homo sapiens OX=9606 GN=APOB PE=1 SV=2	7.05	515.3
Q9NUQ9	CYFIP-related Rac1 interactor B OS=Homo sapiens OX=9606 GN=CYRIB PE=1 SV=1	6.06	36.7
P22894	Neutrophil collagenase OS=Homo sapiens OX=9606 GN=MMP8 PE=1 SV=1	6.87	53.4
P25705	ATP synthase subunit alpha, mitochondrial OS=Homo sapiens OX=9606 GN=ATP5F1A PE=1 SV=1	9.13	59.7
P28676	Grancalcin OS=Homo sapiens OX=9606 GN=GCA PE=1 SV=2	5.21	24
Q9Y2E5	Epididymis-specific alpha-mannosidase OS=Homo sapiens OX=9606 GN=MAN2B2 PE=1 SV=4	7.24	113.9
P69905	Hemoglobin subunit alpha OS=Homo sapiens OX=9606 GN=HBA1 PE=1 SV=2	8.68	15.2
Q9HD89	Resistin OS=Homo sapiens OX=9606 GN=RETN PE=1 SV=1	6.86	11.4
Q9UGL9	Cysteine-rich C-terminal protein 1 OS=Homo sapiens OX=9606 GN=CRCT1 PE=1 SV=1	8.66	9.7
O00571	ATP-dependent RNA helicase DDX3X OS=Homo sapiens OX=9606 GN=DDX3X PE=1 SV=3	7.18	73.2
B7Z8V9	Cysteine-rich secretory protein LCCL domain-containing 1 OS=Homo sapiens OX=9606 GN=CRISPLD1 PE=1 SV=1	8.65	35
A0A1X7SBS1	Heterogeneous nuclear ribonucleoprotein U OS=Homo sapiens OX=9606 GN=HNRNPU PE=1 SV=1	5.83	81.7
O00151	PDZ and LIM domain protein 1 OS=Homo sapiens OX=9606 GN=PDLIM1 PE=1 SV=4	7.02	36
P61978	Heterogeneous nuclear ribonucleoprotein K OS=Homo sapiens OX=9606 GN=HNRNPK PE=1 SV=1	5.54	50.9
P04003	C4b-binding protein alpha chain OS=Homo sapiens OX=9606 GN=C4BPA PE=1 SV=2	7.3	67
P07476	Involucrin OS=Homo sapiens OX=9606 GN=IVL PE=1 SV=2	4.61	68.4
P80723	Brain acid soluble protein 1 OS=Homo sapiens OX=9606 GN=BASP1 PE=1 SV=2	4.63	22.7
Q9Y624	Junctional adhesion molecule A OS=Homo sapiens OX=9606 GN=F11R PE=1 SV=1	7.9	32.6
P25787	Proteasome subunit alpha type-2 OS=Homo sapiens OX=9606 GN=PSMA2 PE=1 SV=2	7.43	25.9
P09525	Annexin A4 OS=Homo sapiens OX=9606 GN=ANXA4 PE=1 SV=4	6.13	35.9
F6S2S5	LIM and SH3 domain protein 1 OS=Homo sapiens OX=9606 GN=LASP1 PE=1 SV=1	9.17	10.6
Q07065	Cytoskeleton-associated protein 4 OS=Homo sapiens OX=9606 GN=CKAP4 PE=1 SV=2	5.92	66
E9PBF6	Lamin-B1 OS=Homo sapiens OX=9606 GN=LMNB1 PE=1 SV=1	5.05	44.6
H0YKU1	Tropomodulin-3 (Fragment) OS=Homo sapiens OX=9606 GN=TMOD3 PE=1 SV=1	7.43	20.9
P51991	Heterogeneous nuclear ribonucleoprotein A3 OS=Homo sapiens OX=9606 GN=HNRNPA3 PE=1 SV=2	9.01	39.6

Q6ZMJ4	Interleukin-34 OS=Homo sapiens OX=9606 GN=IL34 PE=1 SV=1	7.21	27.5
A0A6Q8PGE6	Ras-related protein Rab-7a OS=Homo sapiens OX=9606 GN=RAB7A PE=4 SV=1	5.15	21
O43240	Kallikrein-10 OS=Homo sapiens OX=9606 GN=KLK10 PE=1 SV=3	8.59	30.2
P55000	Secreted Ly-6/uPAR-related protein 1 OS=Homo sapiens OX=9606 GN=SLURP1 PE=1 SV=2	5.33	11.2
C9JDR0	Sterol-4-alpha-carboxylate 3-dehydrogenase, decarboxylating (Fragment) OS=Homo sapiens OX=9606 GN=NSDHL PE=1 SV=1	6.54	28.1
P30050	60S ribosomal protein L12 OS=Homo sapiens OX=9606 GN=RPL12 PE=1 SV=1	9.42	17.8
Q9UJU6	Drebrin-like protein OS=Homo sapiens OX=9606 GN=DBNL PE=1 SV=1	5.05	48.2
P19957	Elafin OS=Homo sapiens OX=9606 GN=PI3 PE=1 SV=3	8.82	12.3
P08185	Corticosteroid-binding globulin OS=Homo sapiens OX=9606 GN=SERPINA6 PE=1 SV=1	6.04	45.1
C9JPM3	Disintegrin and metalloproteinase domain-containing protein 9 OS=Homo sapiens OX=9606 GN=ADAM9 PE=1 SV=1	8.95	16.8
Q16610	Extracellular matrix protein 1 OS=Homo sapiens OX=9606 GN=ECM1 PE=1 SV=2	6.71	60.6
P43652	Afamin OS=Homo sapiens OX=9606 GN=AFM PE=1 SV=1	5.9	69
A0A0C4DH30	Probable non-functional immunoglobulin heavy variable 3-16 OS=Homo sapiens OX=9606 GN=IGHV3-16 PE=1 SV=1	9.73	13.1
P62857	40S ribosomal protein S28 OS=Homo sapiens OX=9606 GN=RPS28 PE=1 SV=1	10.7	7.8
P30043	Flavin reductase (NADPH) OS=Homo sapiens OX=9606 GN=BLVRB PE=1 SV=3	7.65	22.1
E7EMS7	Dermokine OS=Homo sapiens OX=9606 GN=DMKN PE=1 SV=1	7.3	13.3
P62714	Serine/threonine-protein phosphatase 2A catalytic subunit beta isoform OS=Homo sapiens OX=9606 GN=PPP2CB PE=1 SV=1	5.43	35.6
H3BT58	Coactosin-like protein OS=Homo sapiens OX=9606 GN=COTL1 PE=1 SV=1	8.41	8.2
P04843	Dolichyl-diphosphooligosaccharide--protein glycosyltransferase subunit 1 OS=Homo sapiens OX=9606 GN=RPN1 PE=1 SV=1	6.38	68.5
P12532	Creatine kinase U-type, mitochondrial OS=Homo sapiens OX=9606 GN=CKMT1A PE=1 SV=1	8.34	47
A0A075B6I1	Immunoglobulin lambda variable 4-60 OS=Homo sapiens OX=9606 GN=IGLV4-60 PE=3 SV=1	6.25	13
P00966	Argininosuccinate synthase OS=Homo sapiens OX=9606 GN=ASS1 PE=1 SV=2	8.02	46.5
P35754	Glutaredoxin-1 OS=Homo sapiens OX=9606 GN=GLRX PE=1 SV=2	8.09	11.8
P07741	Adenine phosphoribosyltransferase OS=Homo sapiens OX=9606 GN=APRT PE=1 SV=2	6.02	19.6
Q13315	Serine-protein kinase ATM OS=Homo sapiens OX=9606 GN=ATM PE=1 SV=4	6.81	350.5
P28066	Proteasome subunit alpha type-5 OS=Homo sapiens OX=9606 GN=PSMA5 PE=1 SV=3	4.79	26.4
P20160	Azurocidin OS=Homo sapiens OX=9606 GN=AZU1 PE=1 SV=3	9.5	26.9
P39687	Acidic leucine-rich nuclear phosphoprotein 32 family member A OS=Homo sapiens OX=9606 GN=ANP32A PE=1 SV=1	4.09	28.6
A0A2R8Y849	40S ribosomal protein S24 OS=Homo sapiens OX=9606 GN=RPS24 PE=1 SV=1	10.9	15.2

P84095	Rho-related GTP-binding protein RhoG OS=Homo sapiens OX=9606 GN=RHOG PE=1 SV=1	8.12	21.3
G3V0G6	Elastin OS=Homo sapiens OX=9606 GN=ELN PE=1 SV=1	10.42	60.7
Q9BQ50	Three prime repair exonuclease 2 OS=Homo sapiens OX=9606 GN=TREX2 PE=1 SV=2	6.06	25.9
Q00796	Sorbitol dehydrogenase OS=Homo sapiens OX=9606 GN=SORD PE=1 SV=4	7.97	38.3
Q6X4U4	Sclerostin domain-containing protein 1 OS=Homo sapiens OX=9606 GN=SOSTDC1 PE=1 SV=2	9.74	23.3
P38606	V-type proton ATPase catalytic subunit A OS=Homo sapiens OX=9606 GN=ATP6V1A PE=1 SV=2	5.52	68.3
P51688	N-sulphoglucosamine sulphohydrolase OS=Homo sapiens OX=9606 GN=SGSH PE=1 SV=1	6.95	56.7
Q9NZV1	Cysteine-rich motor neuron 1 protein OS=Homo sapiens OX=9606 GN=CRIM1 PE=1 SV=1	5.21	113.7
P0DTE2	Probable non-functional immunoglobulin heavy variable 8-51-1 OS=Homo sapiens OX=9606 GN=IGHV8-51-1 PE=1 SV=1	7.3	13.1
P20618	Proteasome subunit beta type-1 OS=Homo sapiens OX=9606 GN=PSMB1 PE=1 SV=2	8.13	26.5
Q14134	Tripartite motif-containing protein 29 OS=Homo sapiens OX=9606 GN=TRIM29 PE=1 SV=2	7.15	65.8
A0A024R571	EH domain-containing protein 1 OS=Homo sapiens OX=9606 GN=EHD1 PE=1 SV=1	6.71	61.9
C9JX20	Krueppel-like factor 7 (Fragment) OS=Homo sapiens OX=9606 GN=KLF7 PE=1 SV=1	5.15	18.5
C9JD84	Latent-transforming growth factor beta-binding protein 1 OS=Homo sapiens OX=9606 GN=LTBP1 PE=1 SV=1	4.91	146.9
A0A0A0MSV6	Complement C1q subcomponent subunit B (Fragment) OS=Homo sapiens OX=9606 GN=C1QB PE=1 SV=6	9.16	24
M0R261	6-phosphogluconolactonase (Fragment) OS=Homo sapiens OX=9606 GN=PGLS PE=1 SV=1	5.86	23
P12724	Eosinophil cationic protein OS=Homo sapiens OX=9606 GN=RNASE3 PE=1 SV=2	10.02	18.4
Q6ZN66	Guanylate-binding protein 6 OS=Homo sapiens OX=9606 GN=GBP6 PE=2 SV=1	6.37	72.4
Q96C19	EF-hand domain-containing protein D2 OS=Homo sapiens OX=9606 GN=EFHD2 PE=1 SV=1	5.2	26.7
F8W156	Coatomer subunit zeta OS=Homo sapiens OX=9606 GN=COPZ1 PE=1 SV=1	5.06	12
Q99685	Monoglyceride lipase OS=Homo sapiens OX=9606 GN=MGLL PE=1 SV=2	6.99	33.2
Q15149	Plectin OS=Homo sapiens OX=9606 GN=PLEC PE=1 SV=3	5.96	531.5
P61009	Signal peptidase complex subunit 3 OS=Homo sapiens OX=9606 GN=SPCS3 PE=1 SV=1	8.62	20.3
P50552	Vasodilator-stimulated phosphoprotein OS=Homo sapiens OX=9606 GN=VASP PE=1 SV=3	8.94	39.8
P11215	Integrin alpha-M OS=Homo sapiens OX=9606 GN=ITGAM PE=1 SV=2	7.23	127.1
P23490	Loricrin OS=Homo sapiens OX=9606 GN=LORICRIN PE=1 SV=2	8.09	25.7
Q13938	Calcyphosin OS=Homo sapiens OX=9606 GN=CAPS PE=1 SV=2	6.04	30.2

Q02878	60S ribosomal protein L6 OS=Homo sapiens OX=9606 GN=RPL6 PE=1 SV=3	10.58	32.7
C9J155	Neutrophil cytosol factor 1 (Fragment) OS=Homo sapiens OX=9606 GN=NCF1 PE=1 SV=2	9.23	19.4
P25786	Proteasome subunit alpha type-1 OS=Homo sapiens OX=9606 GN=PSMA1 PE=1 SV=1	6.61	29.5
Q9Y446	Plakophilin-3 OS=Homo sapiens OX=9606 GN=PKP3 PE=1 SV=1	9.32	87
A2ABF4	Valine--tRNA ligase (Fragment) OS=Homo sapiens OX=9606 GN=VARS1 PE=1 SV=1	6.77	18.1
M0R0R2	40S ribosomal protein S5 OS=Homo sapiens OX=9606 GN=RPS5 PE=1 SV=1	9.76	25.3
Q10471	Polypeptide N-acetylgalactosaminyltransferase 2 OS=Homo sapiens OX=9606 GN=GALNT2 PE=1 SV=1	8.35	64.7
A0A494C1U9	Neurogenic locus notch homolog protein 2 OS=Homo sapiens OX=9606 GN=NOTCH2 PE=4 SV=1	7.12	29.7
A0A0B4J288	Cadherin-16 OS=Homo sapiens OX=9606 GN=CDH16 PE=1 SV=1	5.11	19.2
Q92520	Protein FAM3C OS=Homo sapiens OX=9606 GN=FAM3C PE=1 SV=1	8.29	24.7
F8WD41	Serum paraoxonase/lactonase 3 OS=Homo sapiens OX=9606 GN=PON3 PE=1 SV=1	5.11	30.7
E9PKZ0	60S ribosomal protein L8 (Fragment) OS=Homo sapiens OX=9606 GN=RPL8 PE=1 SV=1	10.76	22.4
A0A087WUZ2	IgGFc-binding protein OS=Homo sapiens OX=9606 GN=FCGBP PE=1 SV=1	5.12	174
P53004	Biliverdin reductase A OS=Homo sapiens OX=9606 GN=BLVRA PE=1 SV=2	6.44	33.4
O00468	Agrin OS=Homo sapiens OX=9606 GN=AGRN PE=1 SV=6	6.39	217.2
P39060	Collagen alpha-1(XVIII) chain OS=Homo sapiens OX=9606 GN=COL18A1 PE=1 SV=5	6.01	178.1
A0A087WZC4	Protein-glutamine gamma-glutamyltransferase 5 OS=Homo sapiens OX=9606 GN=TGM5 PE=1 SV=1	6.35	71.7
P02748	Complement component C9 OS=Homo sapiens OX=9606 GN=C9 PE=1 SV=2	5.59	63.1
Q8WU39	Marginal zone B- and B1-cell-specific protein OS=Homo sapiens OX=9606 GN=MZB1 PE=1 SV=1	5.57	20.7
A6NLN1	Polypyrimidine tract-binding protein 1 OS=Homo sapiens OX=9606 GN=PTBP1 PE=1 SV=4	9.38	56.5
Q9BVC6	Transmembrane protein 109 OS=Homo sapiens OX=9606 GN=TMEM109 PE=1 SV=1	10.48	26.2
Q9BYE2	Transmembrane protease serine 13 OS=Homo sapiens OX=9606 GN=TMPRSS13 PE=2 SV=5	8.63	63.1
F8WE71	Serine/threonine-protein phosphatase PP1-beta catalytic subunit OS=Homo sapiens OX=9606 GN=PPP1CB PE=1 SV=1	5.25	6.9
A0A0G2JMI3	Immunoglobulin heavy variable 1-69-2 OS=Homo sapiens OX=9606 GN=IGHV1-69-2 PE=3 SV=2	4.84	12.9
Q9BR76	Coronin-1B OS=Homo sapiens OX=9606 GN=CORO1B PE=1 SV=1	5.88	54.2
Q5VVQ6	Ubiquitin thioesterase OTU1 OS=Homo sapiens OX=9606 GN=YOD1 PE=1 SV=1	6.11	38.3
P25398	40S ribosomal protein S12 OS=Homo sapiens OX=9606 GN=RPS12 PE=1 SV=3	7.21	14.5

O95867	Lymphocyte antigen 6 complex locus protein G6c OS=Homo sapiens OX=9606 GN=LY6G6C PE=1 SV=1	8.15	13.8
P16401	Histone H1.5 OS=Homo sapiens OX=9606 GN=H1-5 PE=1 SV=3	10.92	22.6
O60234	Glia maturation factor gamma OS=Homo sapiens OX=9606 GN=GMFG PE=1 SV=1	5.26	16.8
Q8IX05	CD302 antigen OS=Homo sapiens OX=9606 GN=CD302 PE=1 SV=1	4.61	26.2
P49908	Selenoprotein P OS=Homo sapiens OX=9606 GN=SELENOP PE=1 SV=3	7.87	43.2
P28070	Proteasome subunit beta type-4 OS=Homo sapiens OX=9606 GN=PSMB4 PE=1 SV=4	5.97	29.2
G3V1M7	Very long-chain-specific acyl-CoA dehydrogenase, mitochondrial (Fragment) OS=Homo sapiens OX=9606 GN=ACADVL PE=1 SV=2	8.75	30.7
P29350	Tyrosine-protein phosphatase non-receptor type 6 OS=Homo sapiens OX=9606 GN=PTPN6 PE=1 SV=1	7.78	67.5
P08138	Tumor necrosis factor receptor superfamily member 16 OS=Homo sapiens OX=9606 GN=NGFR PE=1 SV=1	4.7	45.2
K7ES69	Calponin-2 OS=Homo sapiens OX=9606 GN=CNN2 PE=1 SV=1	9.04	16.5
A0A075B6I4	Immunoglobulin lambda variable 10-54 OS=Homo sapiens OX=9606 GN=IGLV10-54 PE=3 SV=1	8.03	12.4
P47989	Xanthine dehydrogenase/oxidase OS=Homo sapiens OX=9606 GN=XDH PE=1 SV=4	7.66	146.3
O75342	Arachidonate 12-lipoxygenase, 12R-type OS=Homo sapiens OX=9606 GN=ALOX12B PE=1 SV=1	7.64	80.3
E7ET17	Peroxisomal multifunctional enzyme type 2 OS=Homo sapiens OX=9606 GN=HSD17B4 PE=1 SV=1	8.79	64.9
Q96HC4	PDZ and LIM domain protein 5 OS=Homo sapiens OX=9606 GN=PDLIM5 PE=1 SV=5	8.21	63.9
P13866	Sodium/glucose cotransporter 1 OS=Homo sapiens OX=9606 GN=SLC5A1 PE=1 SV=1	7.68	73.4
P30153	Serine/threonine-protein phosphatase 2A 65 kDa regulatory subunit A alpha isoform OS=Homo sapiens OX=9606 GN=PPP2R1A PE=1 SV=4	5.11	65.3
P17174	Aspartate aminotransferase, cytoplasmic OS=Homo sapiens OX=9606 GN=GOT1 PE=1 SV=3	7.01	46.2
Q7L5L3	Lysophospholipase D GDPD3 OS=Homo sapiens OX=9606 GN=GDPD3 PE=1 SV=3	7.97	36.6
F8VVM2	Phosphate carrier protein, mitochondrial OS=Homo sapiens OX=9606 GN=SLC25A3 PE=1 SV=1	9.26	36.1
D6RD67	Methylcrotonoyl-CoA carboxylase beta chain, mitochondrial (Fragment) OS=Homo sapiens OX=9606 GN=MCCC2 PE=1 SV=2	8.29	31.6
P48595	Serpin B10 OS=Homo sapiens OX=9606 GN=SERPINB10 PE=1 SV=1	6.16	45.4

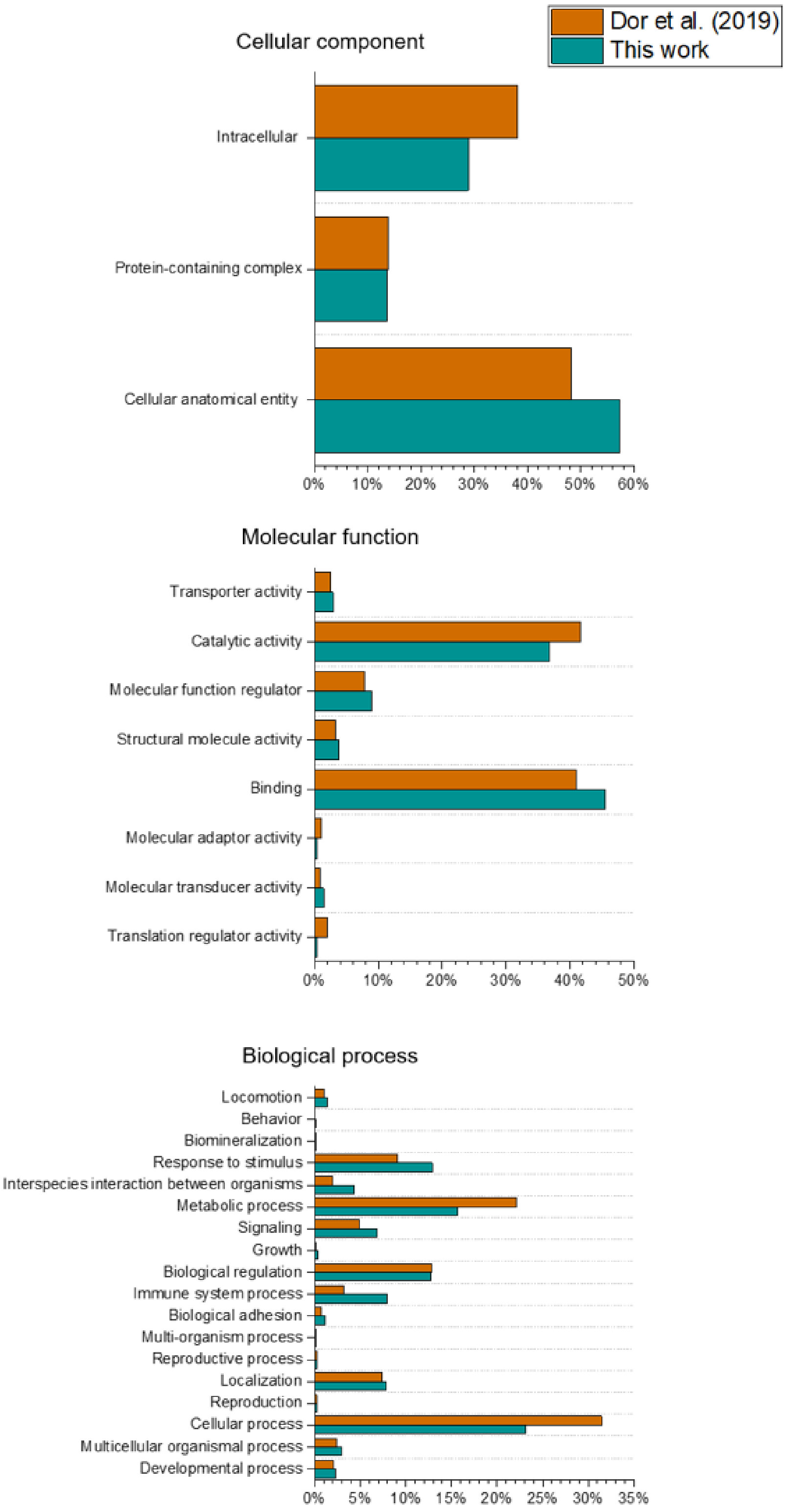

Supporting information S4. Bar charts of the molecular functions, cellular components, and biological processes obtained with the 890 proteins identified in this work, using the PANTHER program. The results are compared with the protein list published by Dor and coworkers.

Supporting information S5: Lysosome pathway. Pink boxes highlight the proteins identified in this work.

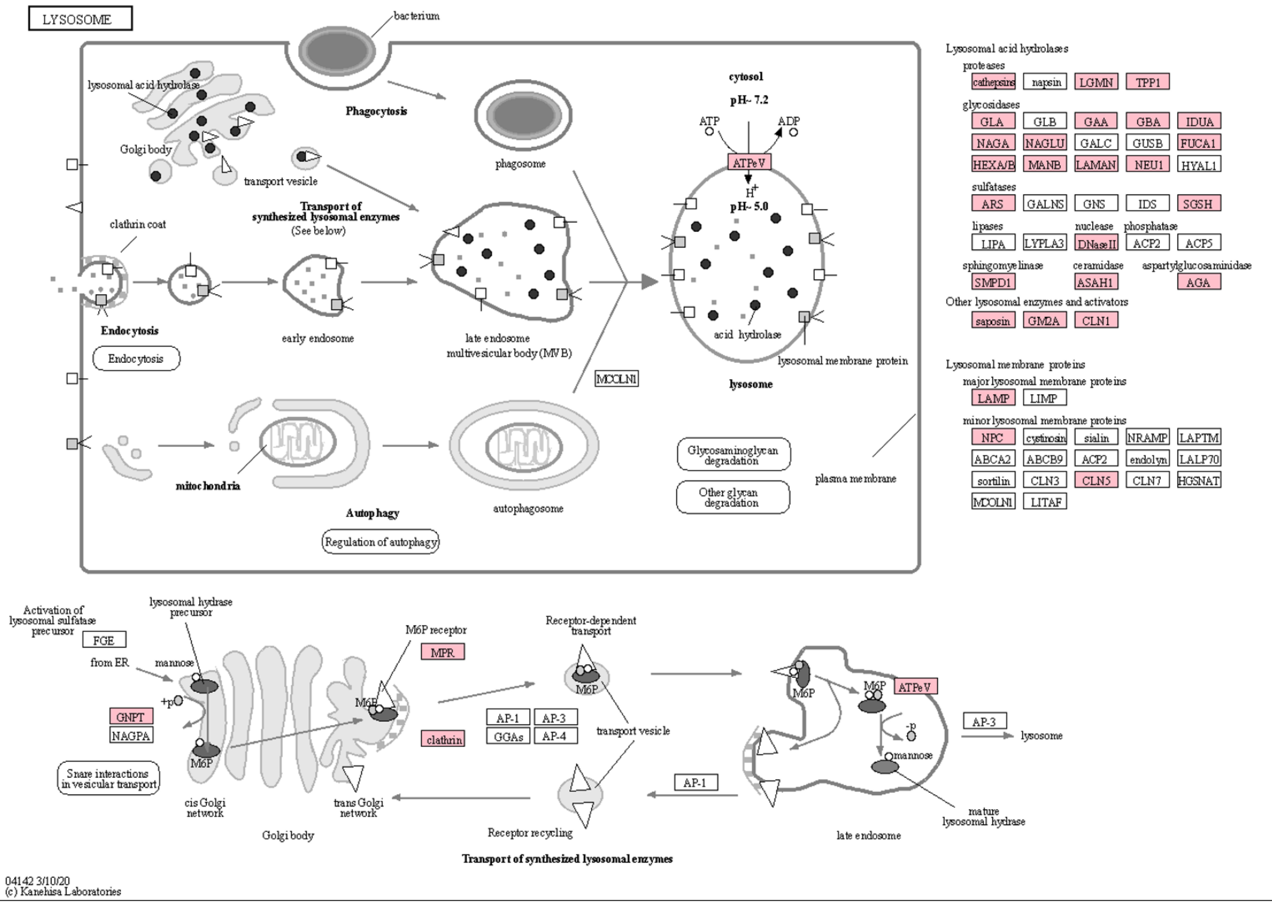

Supporting information S6: Complement and coagulation cascades pathway. Pink boxes highlight the proteins identified in this work.

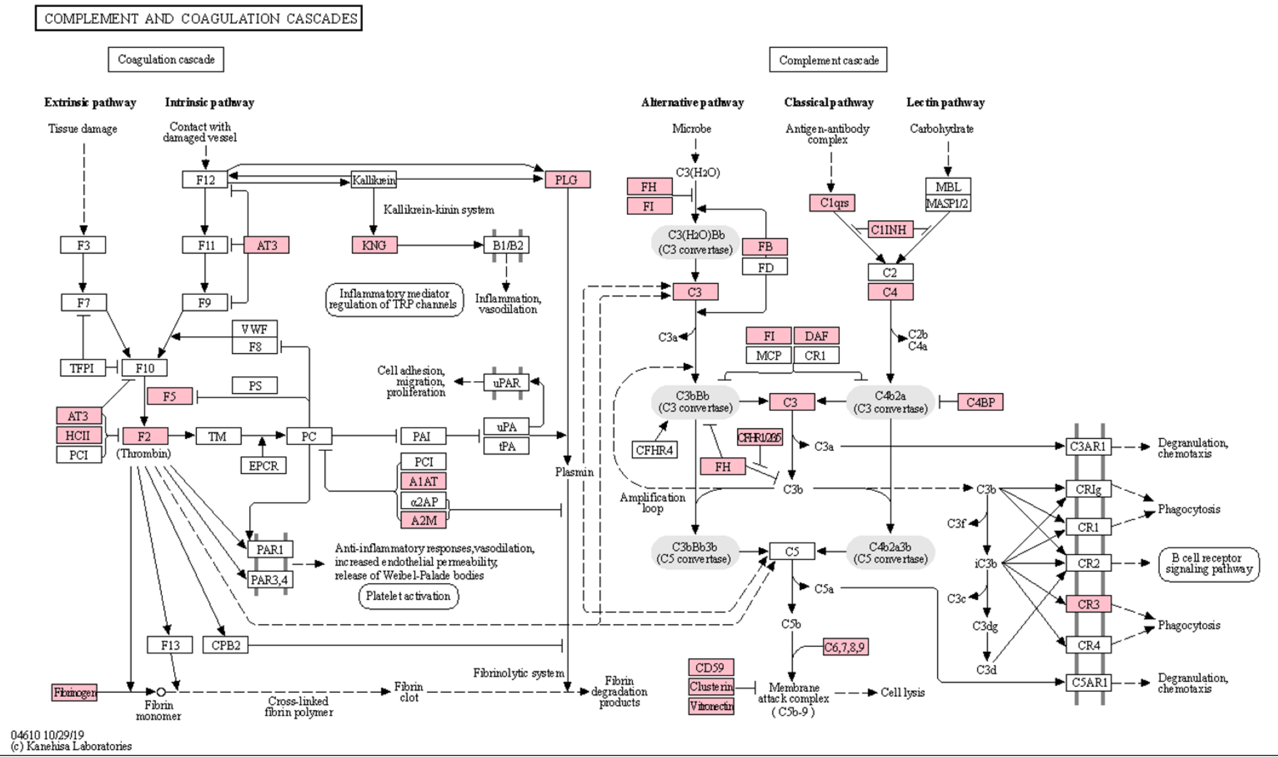

Supporting information S8. List of differentially expressed proteins in Male *vs.* Female subjects.

Accession	Reference	F Ratio	Prob >F	Gene Name	DAVE_MaleVsFemale	DCI
A0A075B6H9	Immunoglobulin lambda variable 4-69	4.33	4.98E-02	IGLV4-69	-1.11	-8.E+08
A0A087X0S5	Collagen alpha-1(VI) chain	16.80	5.13E-04	COL6A1	1.26	2.E+10
A0A0J9YXZ5	Ras GTPase-activating-like protein IQGAP1	5.88	2.45E-02	IQGAP1	0.72	5.E+08
A0A5F9UP49	45 kDa calcium-binding protein	8.61	7.93E-03	SDF4	0.32	2.E+10
C9JPM3	Disintegrin and metalloproteinase domain-containing protein 9	4.58	4.43E-02	ADAM9	0.40	7.E+07
E9PK54	Heat shock cognate 71 kDa protein (Fragment)	10.95	3.33E-03	HSPA8	-2.00	-6.E+08
F8VWW8	Acid sphingomyelinase-like phosphodiesterase 3b	6.61	1.78E-02	SMPDL3B	-1.20	-9.E+08
F8W6I7	Helix-destabilizing protein	5.00	3.63E-02	HNRNPA1	2.00	1.E+09
H0YI00	Extracellular glycoprotein lacritin (Fragment)	10.38	4.09E-03	LACRT	0.74	3.E+12
O14745	Na(+)/H(+) exchange regulatory cofactor NHE-RF1	6.23	2.09E-02	SLC9A3R1	0.89	2.E+09

O43707	Alpha-actinin-4	5.55	2.83E-02	ACTN4	0.84	8.E+09
O75083	WD repeat-containing protein 1	7.64	1.16E-02	WDR1	-1.10	-1.E+10
O75976	Carboxypeptidase D	4.71	4.17E-02	CPD	-0.43	-4.E+08
O95867	Lymphocyte antigen 6 complex locus protein G6c	4.87	3.86E-02	LY6G6C	2.00	5.E+08
P00751	Complement factor B	4.35	4.94E-02	CFB	0.44	2.E+09
P01718	Immunoglobulin lambda variable 3-27	4.73	4.11E-02	IGLV3-27	0.95	2.E+10
P01763	Immunoglobulin heavy variable 3-48	5.08	3.51E-02	IGHV3-48	-1.54	-8.E+09
P01768	Immunoglobulin heavy variable 3-30	6.16	2.16E-02	IGHV3-30	-2.00	-9.E+09
P03973	Antileukoproteinase	7.56	1.20E-02	SLPI	0.60	6.E+12
P05090	Apolipoprotein D	7.89	1.05E-02	APOD	0.63	1.E+11
P07476	Involucrin	6.21	2.11E-02	IVL	1.23	2.E+08
P21980	Protein-glutamine gamma-glutamyltransferase 2	4.64	4.30E-02	TGM2	-2.00	-2.E+09
P22352	Glutathione peroxidase 3	10.32	4.19E-03	GPX3	0.33	6.E+10
P25311	Zinc-alpha-2-glycoprotein	10.46	3.97E-03	AZGP1	0.49	1.E+15
P29350	Tyrosine-protein phosphatase non-receptor type 6	13.92	1.24E-03	PTPN6	2.00	6.E+08
P31025	Lipocalin-1	8.79	7.39E-03	LCN1	-0.29	-6.E+17
P55064	Aquaporin-5	6.19	2.13E-02	AQP5	1.07	2.E+10
P68104	Elongation factor 1-alpha 1	7.45	1.26E-02	EEF1A1	1.08	1.E+11
P81605	Dermcidin	4.40	4.82E-02	DCD	0.79	7.E+11
Q3ZCM7	Tubulin beta-8 chain	8.34	8.81E-03	TUBB8	-0.45	-1.E+09
Q6UXB2	C-X-C motif chemokine 17	7.34	1.31E-02	CXCL17	-0.40	-4.E+10
Q86UD1	Out at first protein homolog	8.03	9.96E-03	OAF	-0.41	-9.E+09

Q8NBJ4	Golgi membrane protein 1	8.31	8.91E-03	GOLM1	**0.48**	**8.E+10**
Q8NBJ7	Inactive C-alpha-formylglycine-generating enzyme 2	7.26	1.36E-02	SUMF2	**0.47**	**3.E+09**
Q8NES3	Beta-1,3-N-acetylglucosaminyltransferase lunatic fringe	5.38	3.06E-02	LFNG	**0.29**	**2.E+09**
Q8NI22	Multiple coagulation factor deficiency protein 2	8.90	7.09E-03	MCFD2	**1.57**	**4.E+09**
Q96S96	Phosphatidylethanolamine-binding protein 4	5.00	3.64E-02	PEBP4	**0.46**	**1.E+11**
Q9P1F3	Costars family protein ABRACL	5.22	3.28E-02	ABRACL	**0.45**	**2.E+09**
Q9UBR2	Cathepsin Z	5.18	3.34E-02	CTSZ	**-0.57**	**-4.E+09**
Q9UN76	Sodium- and chloride-dependent neutral and basic amino acid transporter B(0+)	5.41	3.02E-02	SLC6A14	**0.44**	**3.E+08**

Supporting information S8. List of differentially expressed proteins in Morning *vs.* Afternoon samples, collected from Subject 09 and 13.

					Morning *vs.* Afternoon		Morning *vs.* Afternoon (Subj. 13)		Morning *vs.* Afternoon (Subj. 09)	
**Accession**	**F Ratio**	**Prob>F**	**Reference**	**Gene name**	**DAVE**	**DCI**	**DAVE**	**DCI**	**DAVE**	**DCI**
**A0A075B6I9**	4.97	3.64E-02	Immunoglobulin lambda variable 7-46 OS=Homo sapiens OX=9606 GN=IGLV7-46 PE=3 SV=4	IGLV7-46	0.45	6.97E+12	0.20	2.21E+12	0.68	1.59E+13
**A0A075B6R9**	4.36	4.85E-02	Probable non-functional immunoglobulin kappa variable 2D-24 OS=Homo sapiens OX=9606 GN=IGKV2D-24 PE=1 SV=1	IGKV2D-24	0.42	6.04E+11	0.71	2.74E+10	-1.46	-2.15E+11
**A0A075B6S5**	9.36	5.75E-03	Immunoglobulin kappa variable 1-27 OS=Homo sapiens OX=9606 GN=IGKV1-27 PE=3 SV=1	IGKV1-27	0.49	5.66E+11	0.55	5.55E+11	0.51	6.18E+11
**A0A087WW49**	5.72	2.57E-02	Ig-like domain-containing protein (Fragment) OS=Homo sapiens OX=9606 PE=4 SV=1	A0A087WW49	0.41	1.30E+12	0.43	8.97E+11	0.94	7.76E+12
A0A0B4J1V2	4.70	4.12E-02	Immunoglobulin heavy variable 2-26 OS=Homo sapiens OX=9606 GN=IGHV2-26 PE=3 SV=1	IGHV2-26	1.10	8.02E+10	0.71	8.15E+10	0.00	0.00E+00
**A0A0C4DH38**	9.35	5.76E-03	Immunoglobulin heavy variable 5-51 OS=Homo	IGHV5-51	0.66	3.19E+11	0.44	2.02E+11	0.71	3.52E+11

			sapiens OX=9606 GN=IGHV5-51 PE=3 SV=1							
**A0A0J9YY99**	7.02	1.47E-02	Ig-like domain-containing protein (Fragment) OS=Homo sapiens OX=9606 PE=1 SV=1	A0A0J9YY99	0.53	1.55E+13	0.30	7.19E+12	0.81	2.64E+13
**A0A3B3IRN5**	4.92	3.72E-02	Fibromodulin OS=Homo sapiens OX=9606 GN=FMOD PE=1 SV=1	FMOD	0.85	6.64E+09	1.49	2.18E+10	-1.14	-1.54E+09
**A0A3B3IRX2**	6.07	2.20E-02	Phospholipase A2 OS=Homo sapiens OX=9606 GN=PLA2G2A PE=1 SV=1	PLA2G2A	0.31	5.92E+14	0.51	1.35E+15	0.31	5.06E+14
**A0A5H1ZRS9**	4.55	4.42E-02	Immunoglobulin kappa variable 2D-29 OS=Homo sapiens OX=9606 GN=IGKV2D-29 PE=1 SV=1	IGKV2D-29	0.17	3.01E+12	0.21	3.23E+12	0.87	4.80E+13
**P01599**	4.63	4.26E-02	Immunoglobulin kappa variable 1-17 OS=Homo sapiens OX=9606 GN=IGKV1-17 PE=1 SV=2	IGKV1-17	0.40	4.63E+10	0.39	2.45E+10	0.36	5.17E+10
**P01619**	7.03	1.46E-02	Immunoglobulin kappa variable 3-20 OS=Homo sapiens OX=9606 GN=IGKV3-20 PE=1 SV=2	IGKV3-20	0.49	3.85E+13	0.30	1.92E+13	0.87	1.15E+14
**P01834**	4.51	4.51E-02	Immunoglobulin kappa constant OS=Homo sapiens OX=9606 GN=IGKC PE=1 SV=2	IGKC	0.41	5.97E+15	0.27	3.62E+15	0.74	1.70E+16
**P04430**	4.93	3.69E-02	Immunoglobulin kappa variable 1-16 OS=Homo	IGKV1-16	0.59	9.14E+10	0.32	4.13E+10	0.72	1.17E+11

			sapiens OX=9606 GN=IGKV1-16 PE=1 SV=2							
**P04432**	5.38	3.00E-02	Immunoglobulin kappa variable 1D-39 OS=Homo sapiens OX=9606 GN=IGKV1D-39 PE=3 SV=2	IGKV1D-39	0.53	3.43E+12	0.44	2.34E+12	0.68	5.96E+12
**P06312**	5.46	2.90E-02	Immunoglobulin kappa variable 4-1 OS=Homo sapiens OX=9606 GN=IGKV4-1 PE=1 SV=1	IGKV4-1	0.20	5.95E+11	0.21	4.45E+11	0.95	8.69E+12
**P08571**	10.11	4.34E-03	Monocyte differentiation antigen CD14 OS=Homo sapiens OX=9606 GN=CD14 PE=1 SV=2	CD14	0.46	4.11E+10	0.45	4.21E+10	0.44	3.87E+10
P21246	6.01	2.27E-02	Pleiotrophin OS=Homo sapiens OX=9606 GN=PTN PE=1 SV=1	PTN	1.28	2.48E+09	0.85	2.54E+09	2.00	4.78E+08
P45877	7.28	1.32E-02	Peptidyl-prolyl cis-trans isomerase C OS=Homo sapiens OX=9606 GN=PPIC PE=1 SV=1	PPIC	0.31	6.05E+09	0.07	1.04E+09	0.76	2.30E+10
**P80188**	4.95	3.67E-02	Neutrophil gelatinase-associated lipocalin OS=Homo sapiens OX=9606 GN=LCN2 PE=1 SV=2	LCN2	0.32	2.57E+12	0.29	1.87E+12	0.55	7.91E+12
P98160	4.88	3.79E-02	Basement membrane-specific heparan sulfate proteoglycan core protein OS=Homo sapiens OX=9606 GN=HSPG2 PE=1 SV=4	HSPG2	0.26	1.09E+14	-0.07	-2.70E+13	0.43	1.80E+14

Q11201	5.11	3.40E-02	CMP-N-acetylneuraminate-beta-galactosamide-alpha-2,3-sialyltransferase 1 OS=Homo sapiens OX=9606 GN=ST3GAL1 PE=2 SV=1	ST3GAL1	-2.00	-1.28E+08	0.00	0.00E+00	-2.00	-1.36E+09
**Q15782**	4.49	4.57E-02	Chitinase-3-like protein 2 OS=Homo sapiens OX=9606 GN=CHI3L2 PE=1 SV=1	CHI3L2	0.47	1.95E+11	0.38	2.50E+11	1.82	1.80E+11
**Q99574**	5.32	3.08E-02	Neuroserpin OS=Homo sapiens OX=9606 GN=SERPINI1 PE=1 SV=1	SERPINI1	0.28	4.51E+10	0.38	7.18E+10	0.24	4.21E+10
Q9GZZ8	5.59	2.73E-02	Extracellular glycoprotein lacritin OS=Homo sapiens OX=9606 GN=LACRT PE=1 SV=1	LACRT	-0.89	-7.31E+17	-0.19	-2.51E+16	-0.94	-2.42E+18
**Q9NZ08**	5.37	3.02E-02	Endoplasmic reticulum aminopeptidase 1 OS=Homo sapiens OX=9606 GN=ERAP1 PE=1 SV=3	ERAP1	0.37	4.84E+09	0.60	1.49E+10	0.59	2.61E+09
**Q9UBT3**	4.41	4.74E-02	Dickkopf-related protein 4 OS=Homo sapiens OX=9606 GN=DKK4 PE=1 SV=1	DKK4	-0.36	-8.52E+09	-0.98	-7.76E+10	-0.59	-2.72E+09
